# Supplementary material for: Cas12a-assisted precise targeted cloning using in vivo Cre-lox recombination
Source: Nat Commun. 2021 Feb 19;12:1171. doi: 10.1038/s41467-021-21275-4 (PMC7896053; doi:10.1038/s41467-021-21275-4)
Supplement: Supplementary file 1 — Supplementary Information [file 41467_2021_21275_MOESM1_ESM.docx]

Supplementary Information for

Cas12a assisted precise targeted cloning using *in vivo* Cre-*lox* recombination

**This document includes:**

Supplementary Figures 2

Supplementary Tables 113

Supplementary References 124

**Other Supplementary Data for this manuscript includes the following:**

Supplementary Data 1. Oligonucleotides used in this study (Excel file)

Source Data zip file including a Source Data excel file and cloned BGCs sequences

Supplementary Figures


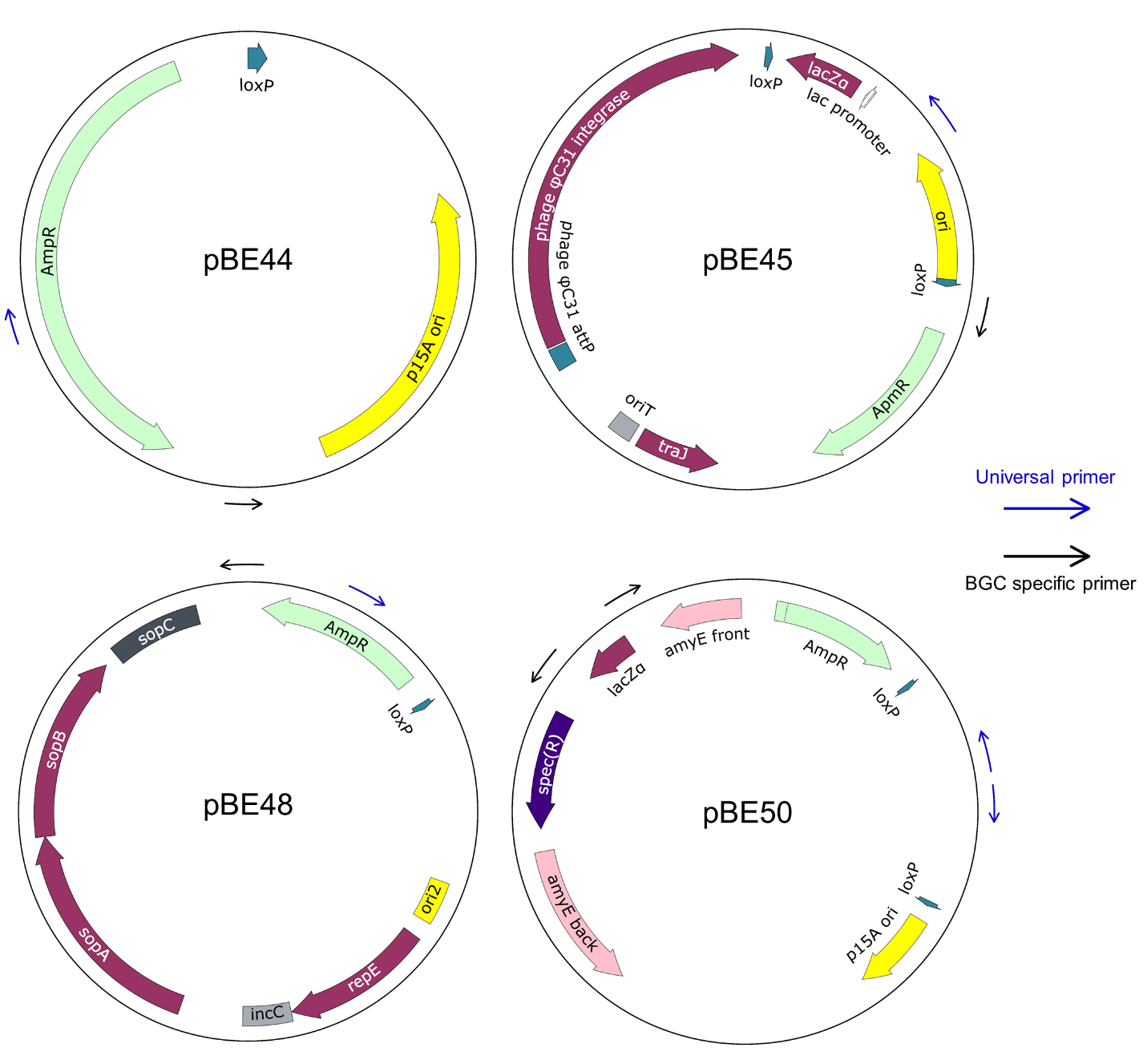


**Supplementary Fig. 1.** DNA maps for designed universal receiver plasmids for *Streptomyces* and *Bacillus subtilis* heterologous hosts. pBE44: *Streptomyces* receiver plasmid designed for amplification of the 15A origin of replication. pBE45: *Streptomyces* receiver plasmid designed for amplification of the apramycin resistance marker and elements required for *Streptomyces* heterologous expression including conjugal transfer and heterologous host integration. pBE48: *Streptomyces* receiver plasmid designed for amplification of the BAC origin of replication. pBE50: *B. subtilis* receiver plasmid designed for amplification of two separate linear DNA receivers: 1) a DNA receiver carrying the ampicillin resistance marker and *amyE* homology arm for *B. subtilis* integration, and 2) a DNA receiver carrying the 15A origin of replication, *B. subtilis* spectinomycin resistance marker and the other homology arm for integration. The linear DNA receivers are amplified using two primers: a universal primer same for all BGCs, and a BGC specific primer designed based on the BGC of interest.

**
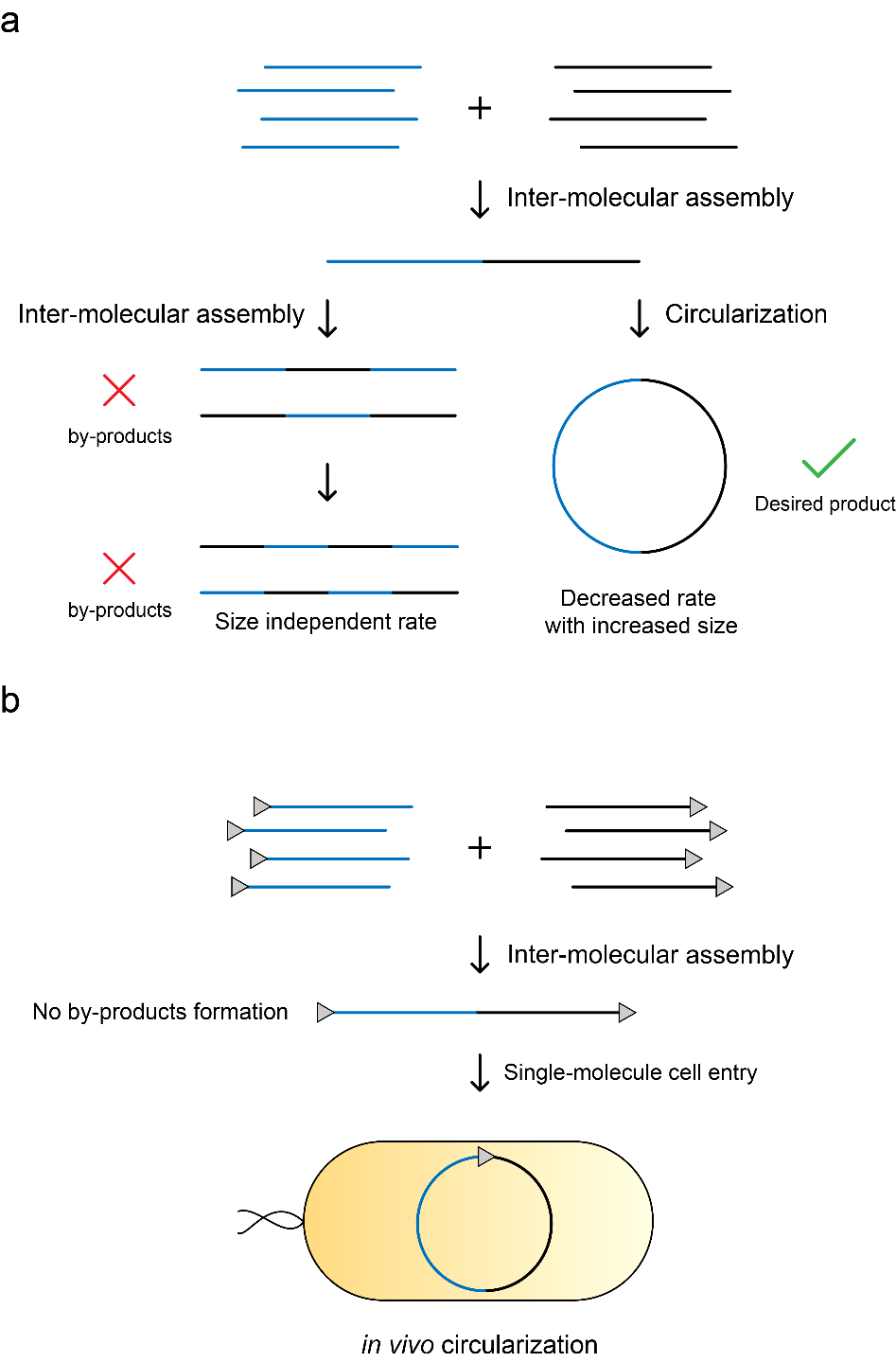
**

**Supplementary Fig. 2.** Comparison of *in vitro* and *in vivo* circularization methods. **a)** *In vitro* assembly comprises two steps: 1) Inter-molecular assembly of two DNA molecules to form a longer linear DNA product; 2) Intra-molecular assembly of the linear product from step 1 to form the desired circular product (*i.e.* circularization). A side reaction after step 1 is creation of by-products such as DNA concatemers. As DNA size increases, rate of the second step or DNA circularization drops significantly^1^. Formation of by-products on the other hand is not directly dependent on DNA size. As a result, as the DNA size increases, the frequency of creating the desired circular product drops significantly. **b)** Assembly of two linear DNA molecules by separating inter-molecular assembly and circularization steps. After inter-molecular assembly of two linear DNA molecules to form a longer linear DNA product, the product is directly transformed into *E. coli* cells and circularizes *in vivo*. By eliminating formation of DNA by-products, the probability of DNA circularization increases.


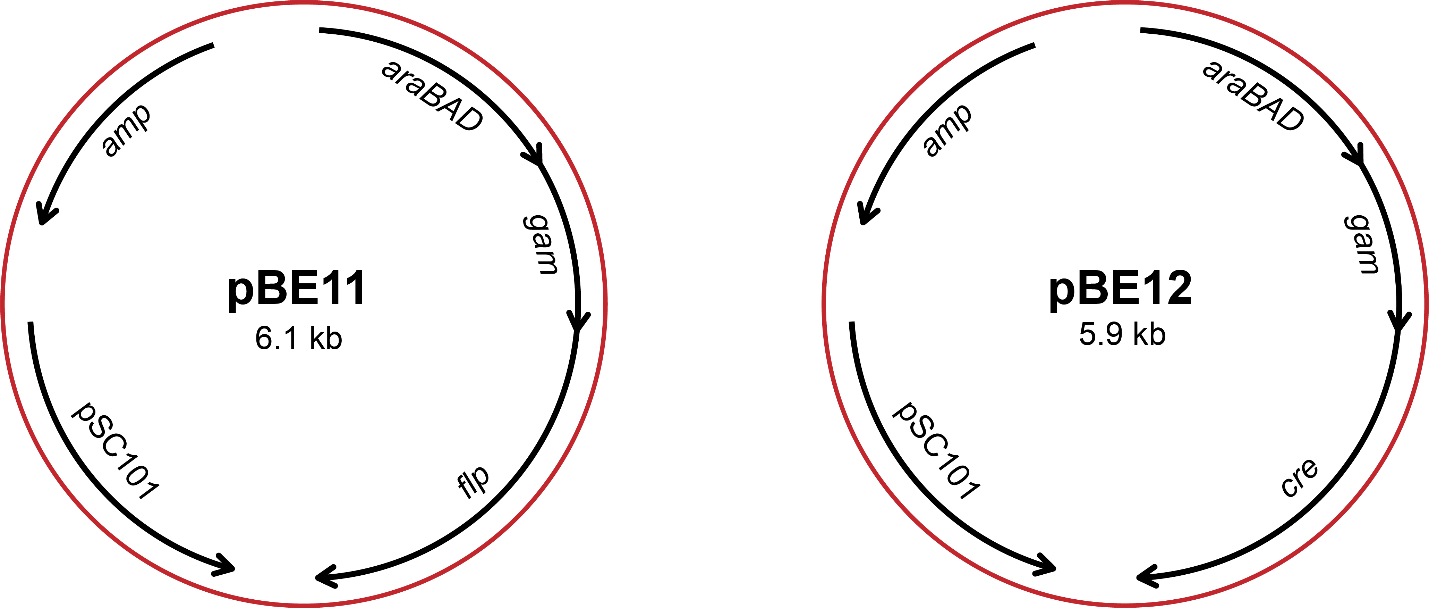


**Supplementary Fig. 3.** DNA maps for Flp (pBE11) and Cre (pBE12) helper plasmids. *araBAD*: L-arabinose inducible promoter and its regulator. *amp*: ampicillin resistance marker.


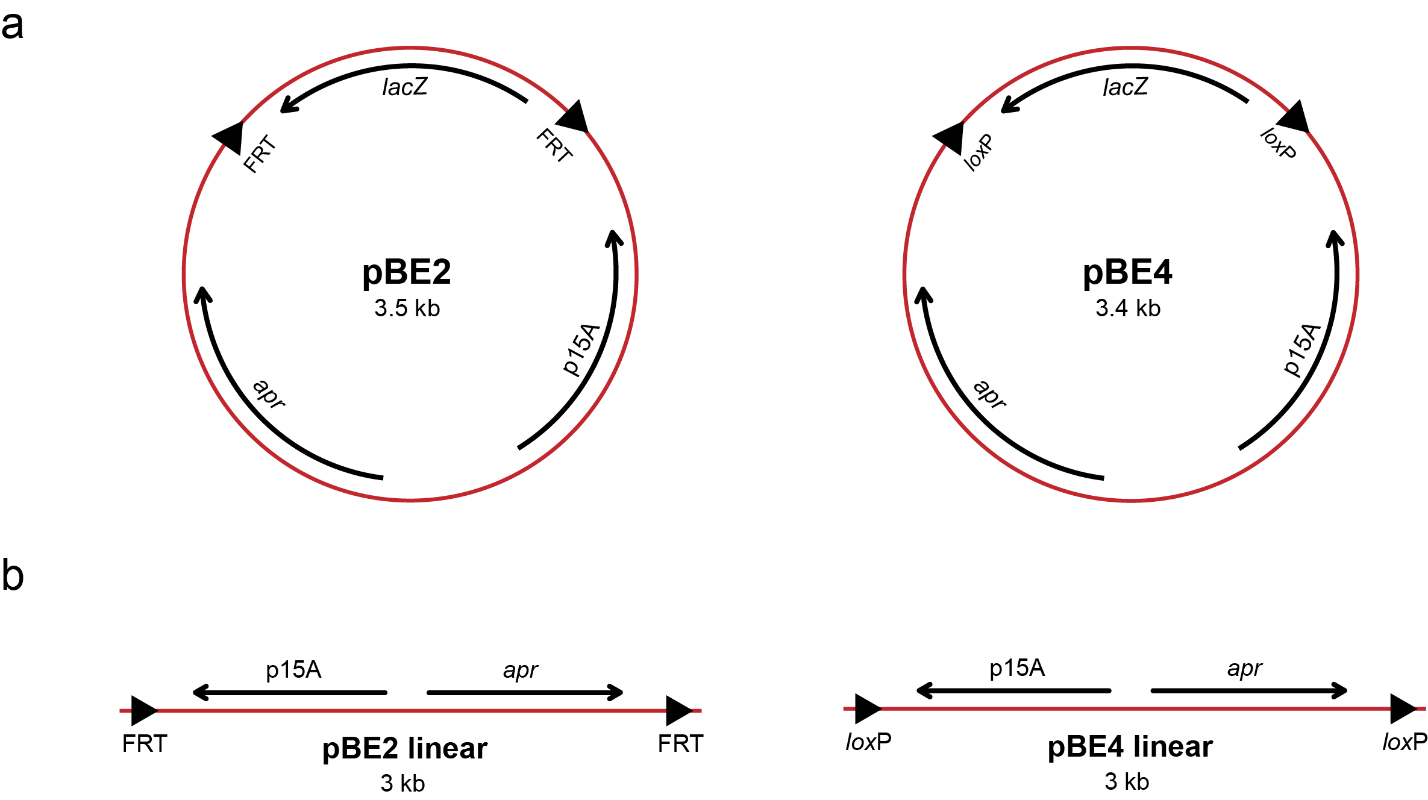


**Supplementary Fig. 4. a)** DNA maps for test plasmids used in site-specific recombinase activity experiments. **b)** DNA maps for linear PCR products used in *in vivo* DNA circularization experiments. p15A: 15A origin of replication. *apr*: apramycin resistance marker.

**Supplementary Fig. 5.** Transformation efficiency for ~3 kb linear and circular test DNA (pBE4) transformed into *E. coli* cells harboring pBE12 helper plasmid. Each experiment was performed in three biological replicates and data are presented as mean values +/− standard deviation (SD). Source data are provided as a Source Data file.

**
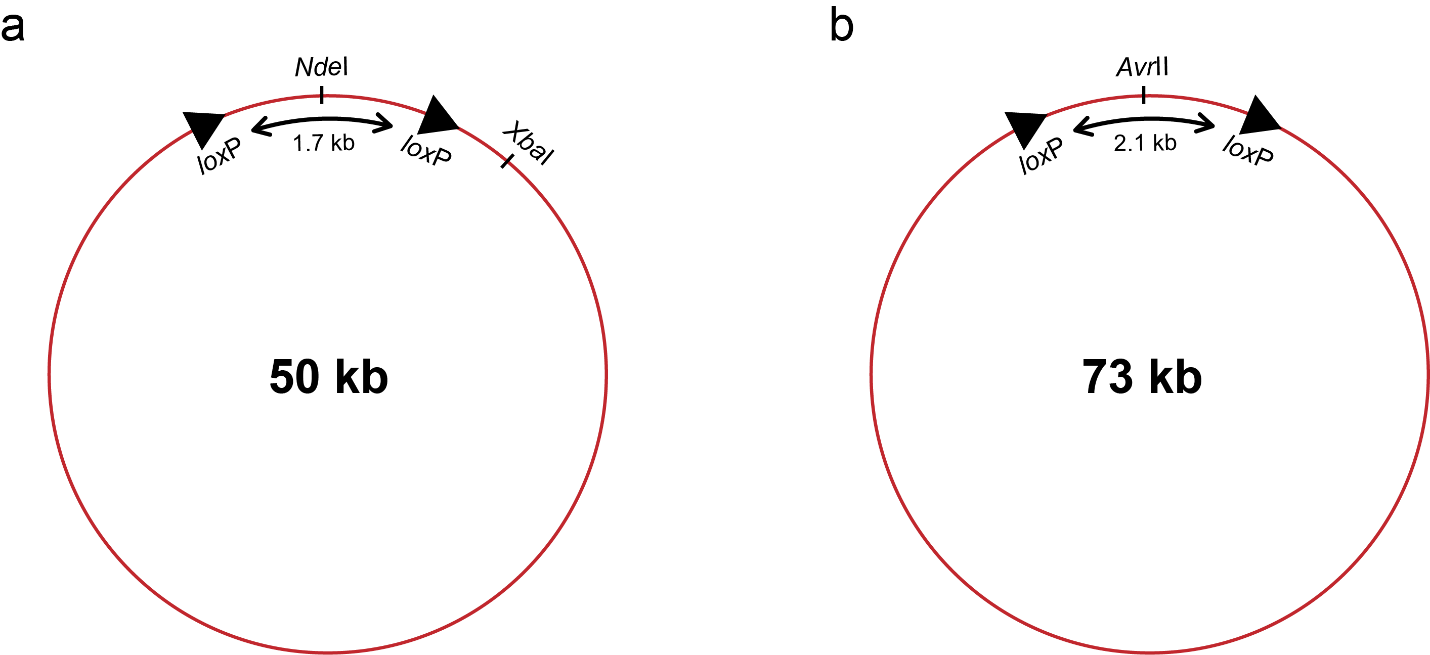
**

**Supplementary Fig. 6.** DNA maps for long DNA molecules used for comparison of the *in vitro* and *in vivo* DNA circularization methods. **a)** 50 kb test plasmid. For *in vitro* test, the plasmid was linearized using *Xba*I restriction enzyme and ligated to itself using T4 DNA ligase. For *in vivo* test, the plasmid was linearized using *Nde*I restriction enzyme and transformed into *E. coli* cells harboring pBE14 helper plasmid. **b)** 73 kb test plasmid. The plasmid was linearized using *Avr*II restriction enzyme for both *in vitro* and *in vivo* experiments.

**
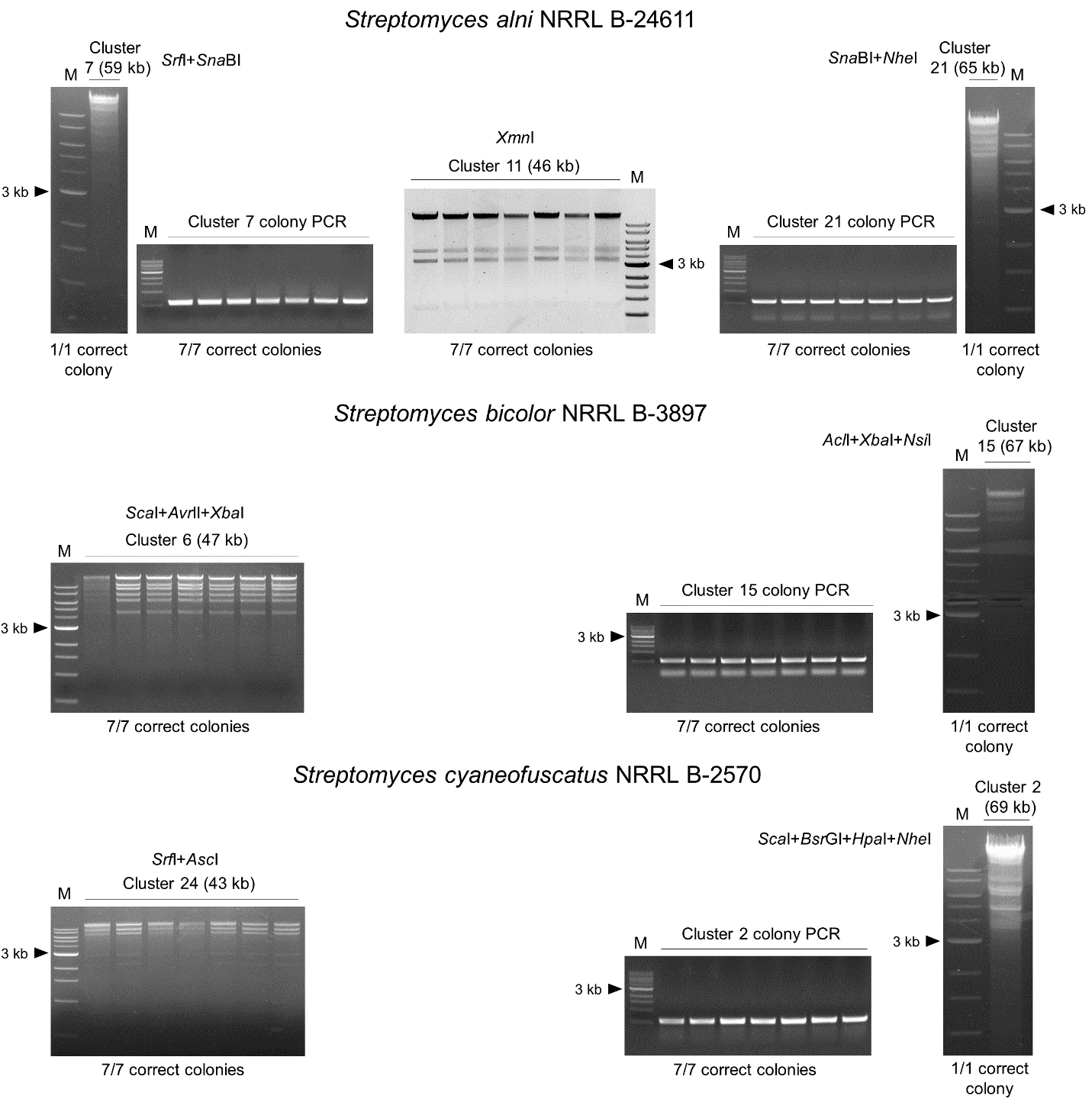
**

**Supplementary Fig. 7.** Restriction digestion analysis of the cloned BGCs from *Streptomyces alni* NRRL B-24611, *Streptomyces bicolor* NRRL B-3897, and *Streptomyces cyaneofuscatus* NRRL B-2570. For BGCs cloned using the BAC origin of replication receiver, the colonies were first checked by colony PCR and at least one colony was then randomly picked and checked by restriction digestion. Restriction enzymes used for each analysis are shown next to the gel image. Each cloning experiment was performed one time. M: 1 kb DNA ladder.

**
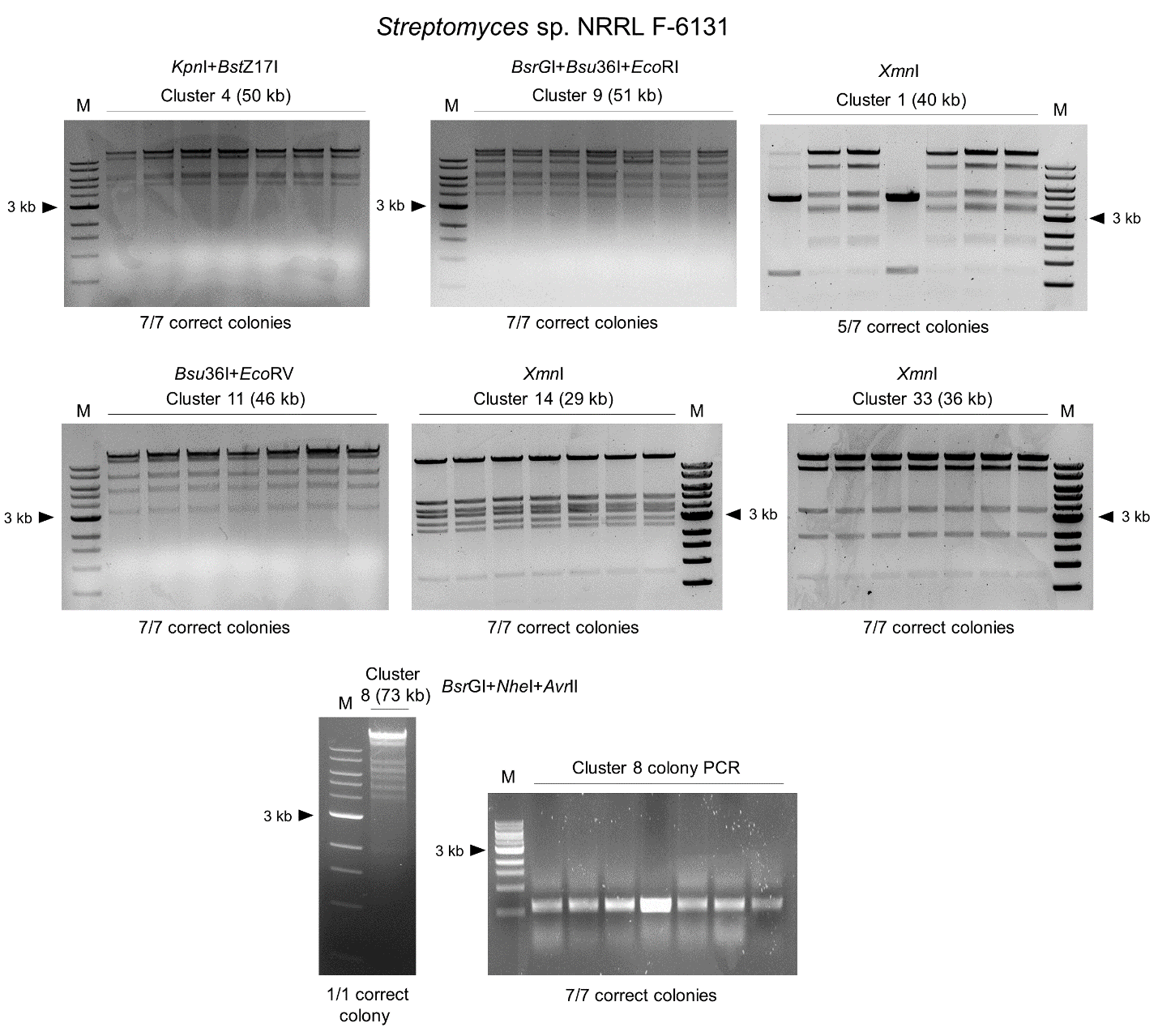
**

**Supplementary Fig. 8.** Restriction digestion analysis of the cloned BGCs from *Streptomyces* sp. NRRL F-6131. For BGCs cloned using the BAC origin of replication receiver, the colonies were first checked by colony PCR and at least one colony was then randomly picked and checked by restriction digestion. Restriction enzymes used for each analysis are shown next to the gel image. Each cloning experiment was performed one time. M: 1 kb DNA ladder.

**
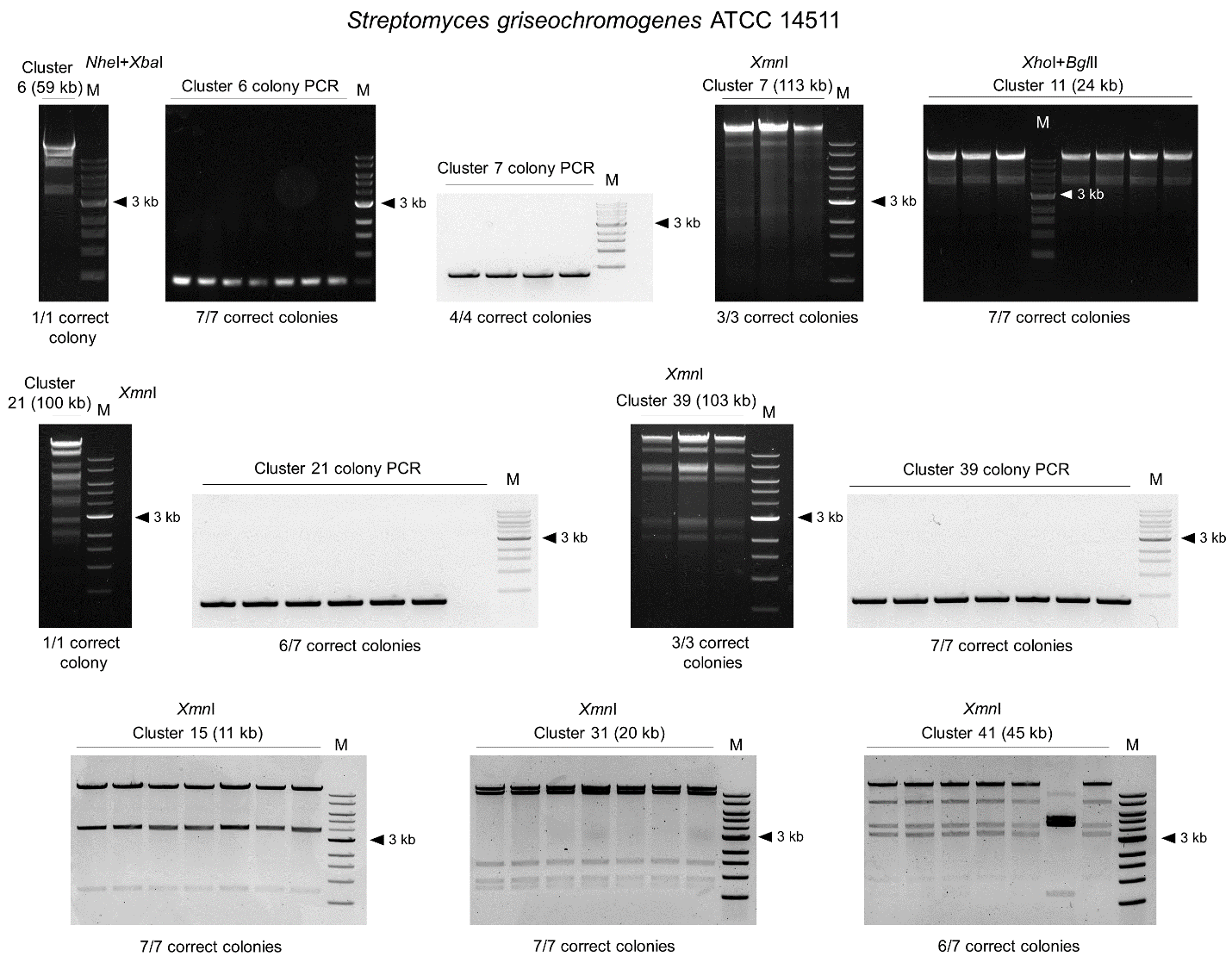
**

**Supplementary Fig. 9.** Restriction digestion analysis of the cloned BGCs from *Streptomyces griseochromogenes* ATCC 14511. For BGCs cloned using the BAC origin of replication receiver, the colonies were first checked by colony PCR and at least one colony was then randomly picked and checked by restriction digestion. Restriction enzymes used for each analysis are shown next to the gel image. For BGCs #7, #21, and #39, the cloning experiments were repeated at least one time with similar results. For the remaining BGCs, each experiment was performed one time. M: 1 kb DNA ladder.

**
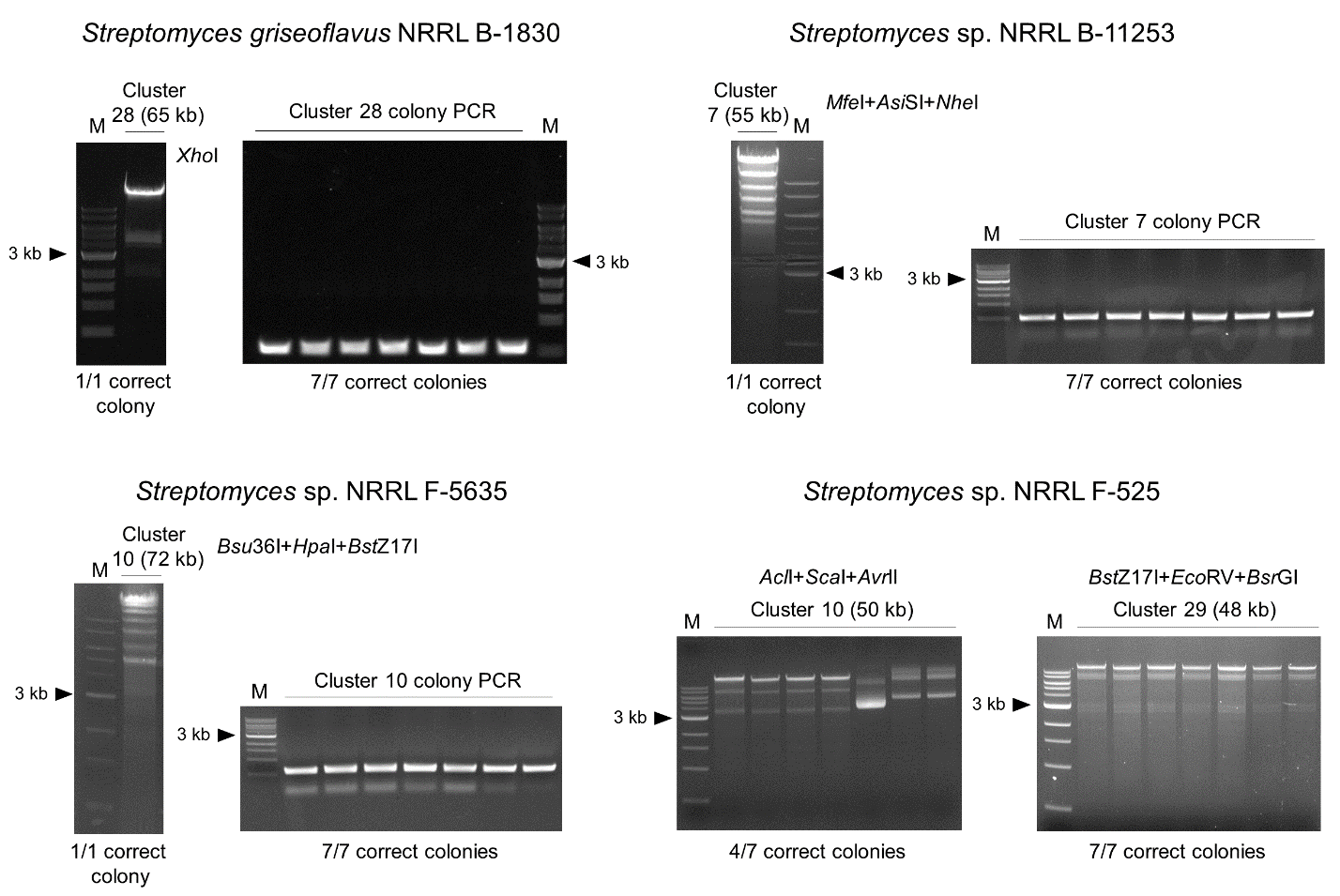
**

**Supplementary Fig. 10.** Restriction digestion analysis of the cloned BGCs from *Streptomyces griseoflavus* NRRL B-24963, *Streptomyces* sp. NRRL B-11253, *Streptomyces* sp. NRRL F-5635, and *Streptomyces* sp. NRRL F-525. For BGCs cloned using the BAC origin of replication receiver, the colonies were first checked by colony PCR and at least one colony was then randomly picked and checked by restriction digestion. Restriction enzymes used for each analysis are shown next to the gel image. Each cloning experiment was performed one time. M: 1 kb DNA ladder.

**
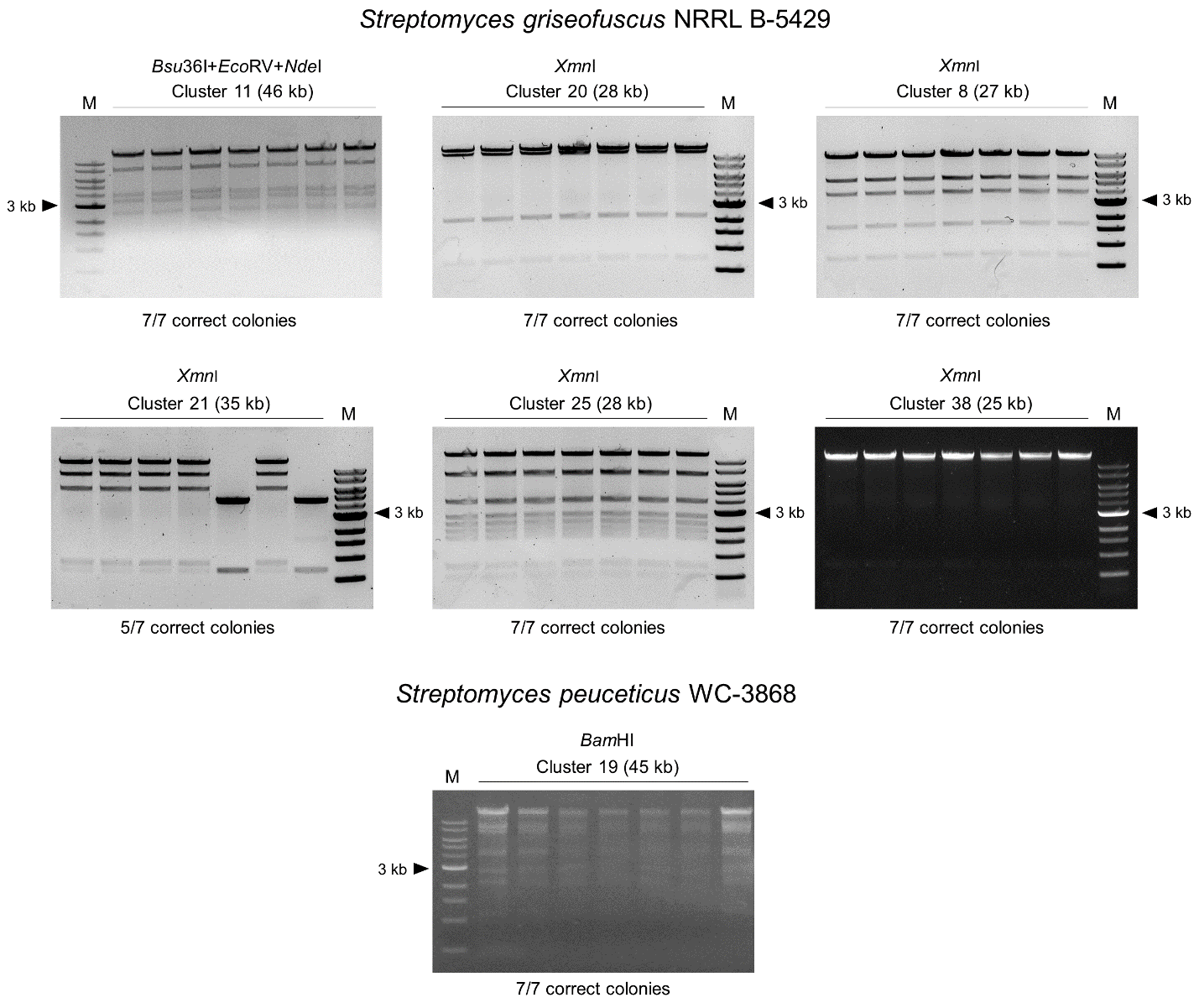
**

**Supplementary Fig. 11.** Restriction digestion analysis of the cloned BGCs from *Streptomyces* *griseofuscus* NRRL B-5429 and *Streptomyces peuceticus* WC-3868. Restriction enzymes used for each analysis are shown next to the gel image. Each cloning experiment was performed one time. M: 1 kb DNA ladder.

**
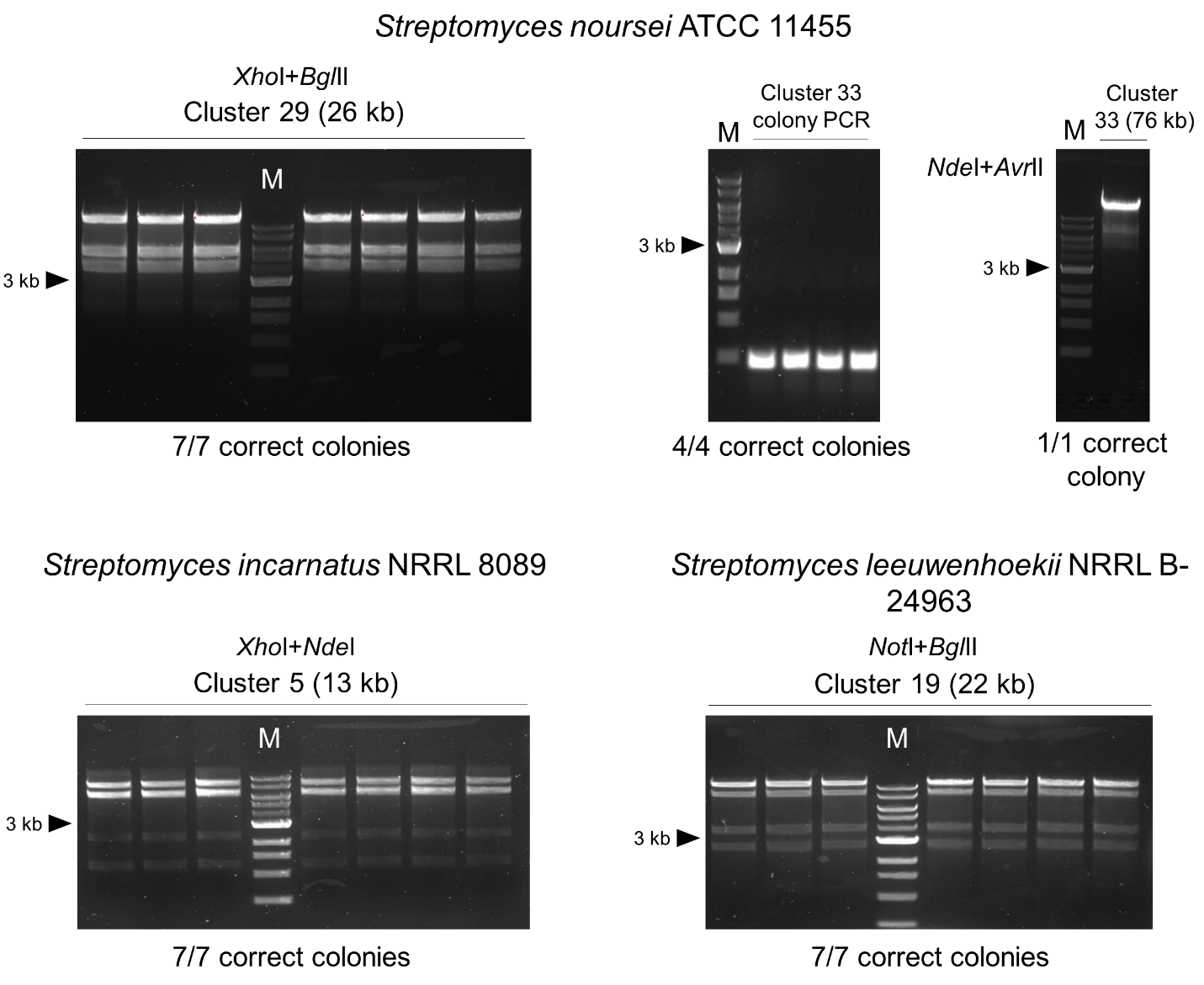
**

**Supplementary Fig. 12.** Restriction digestion analysis of the cloned BGCs from *Streptomyces noursei* ATCC 11455, *Streptomyces incarnatus* NRRL 8089, and *Streptomyces leeuwenhoekii* NRRL B-24963. Restriction enzymes used for each analysis are shown next to the gel image. Each cloning experiment was performed one time. M: 1 kb DNA ladder.


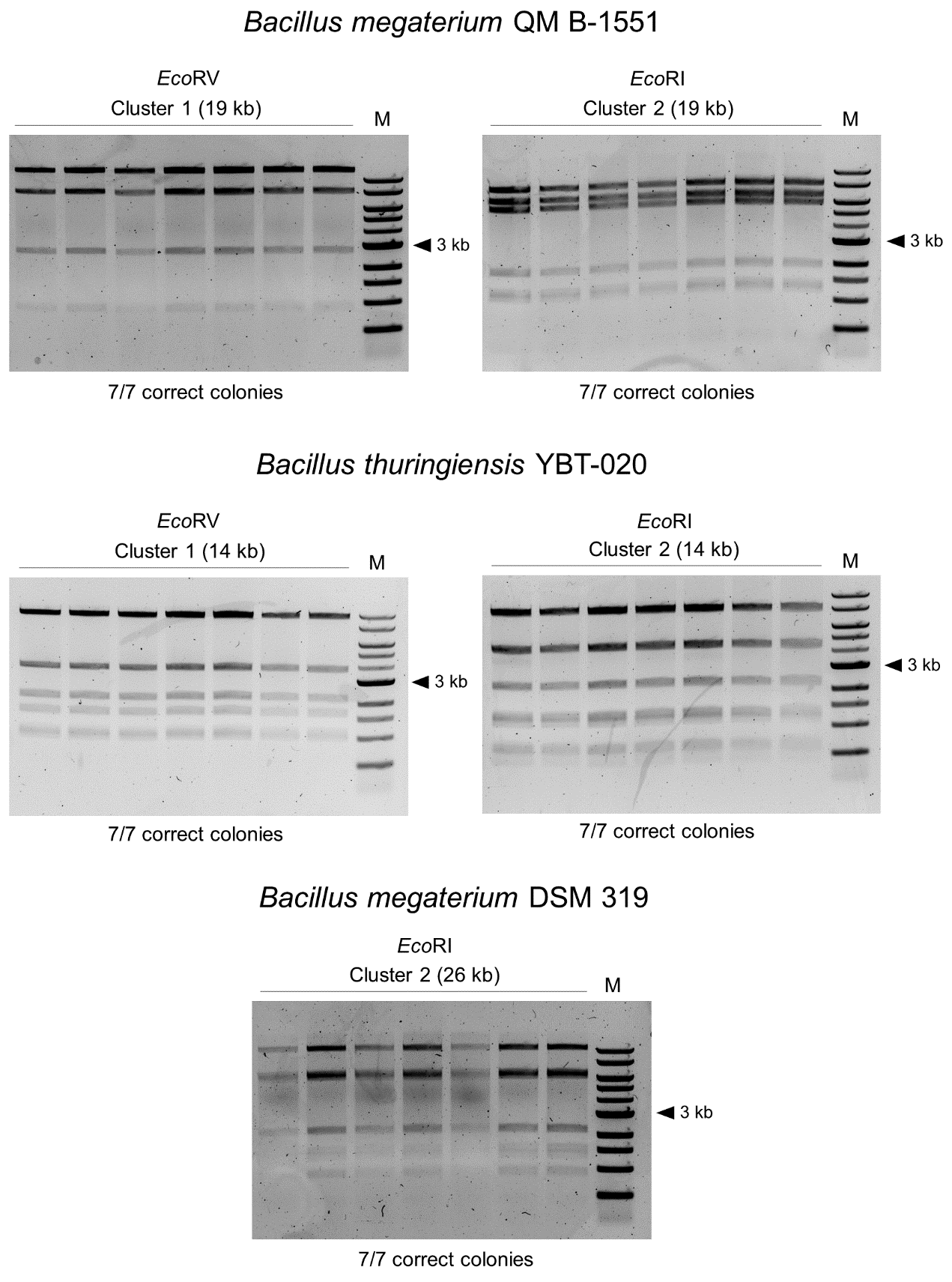


**Supplementary Fig. 13.** Restriction digestion analysis of the cloned BGCs from *Bacillus megaterium* QM B-1551, *Bacillus thuringiensis* YBT-020, and *Bacillus megaterium* DSM 319. Restriction enzymes used for each analysis are shown next to the gel image. Each cloning experiment was performed one time. M: 1 kb DNA ladder.

**
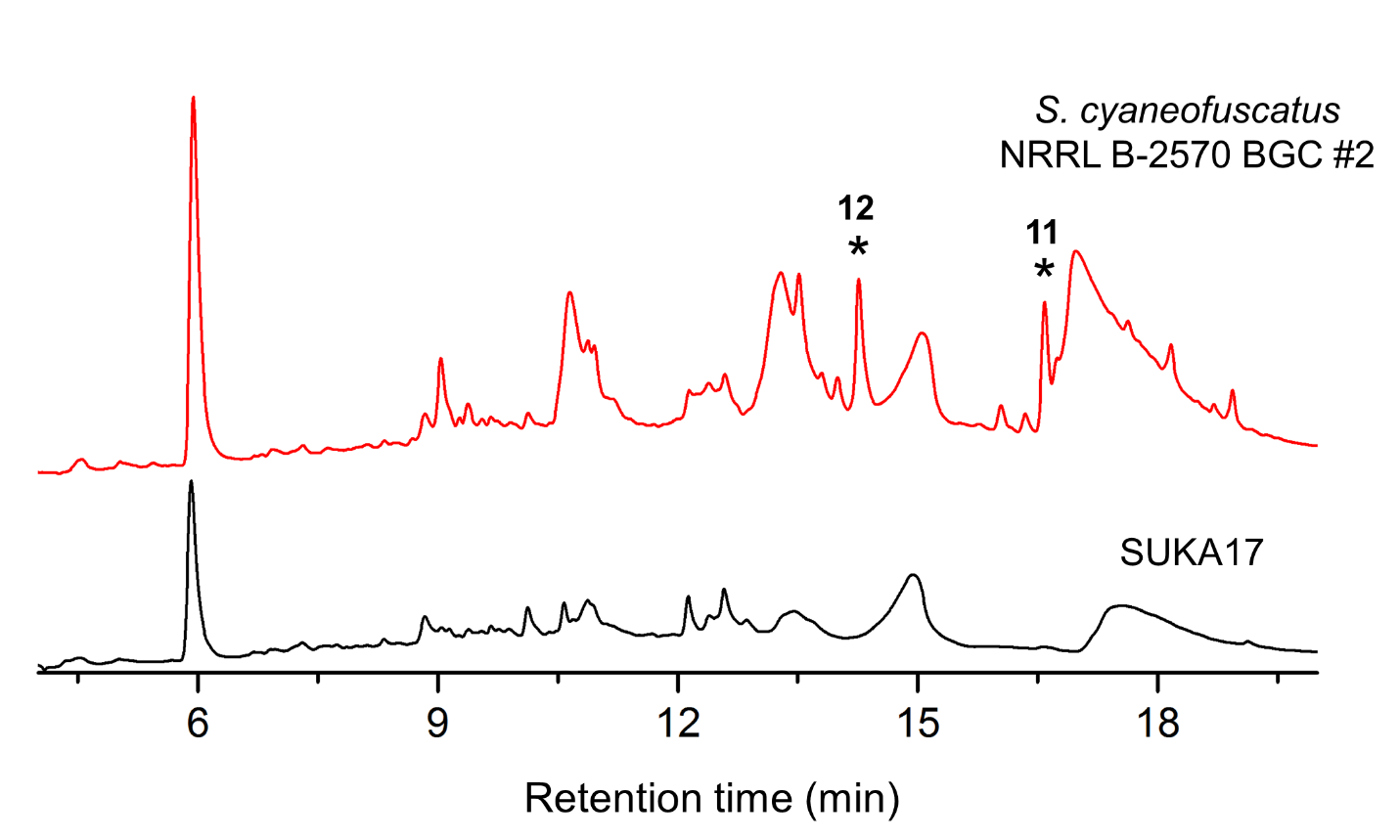
**

**Supplementary Fig. 14.** HPLC analysis of the crude extract from heterologous expression of BGC #2 from *S. cyaneofuscatus* NRRL B-2570 in *S. avermitilis* SUKA17. Corresponding new peaks are labeled with an asterisk and numbers. **11**: *N*-acetylcysteinmansoquinone. **12**: *N-*acetylcysteingriseusin.


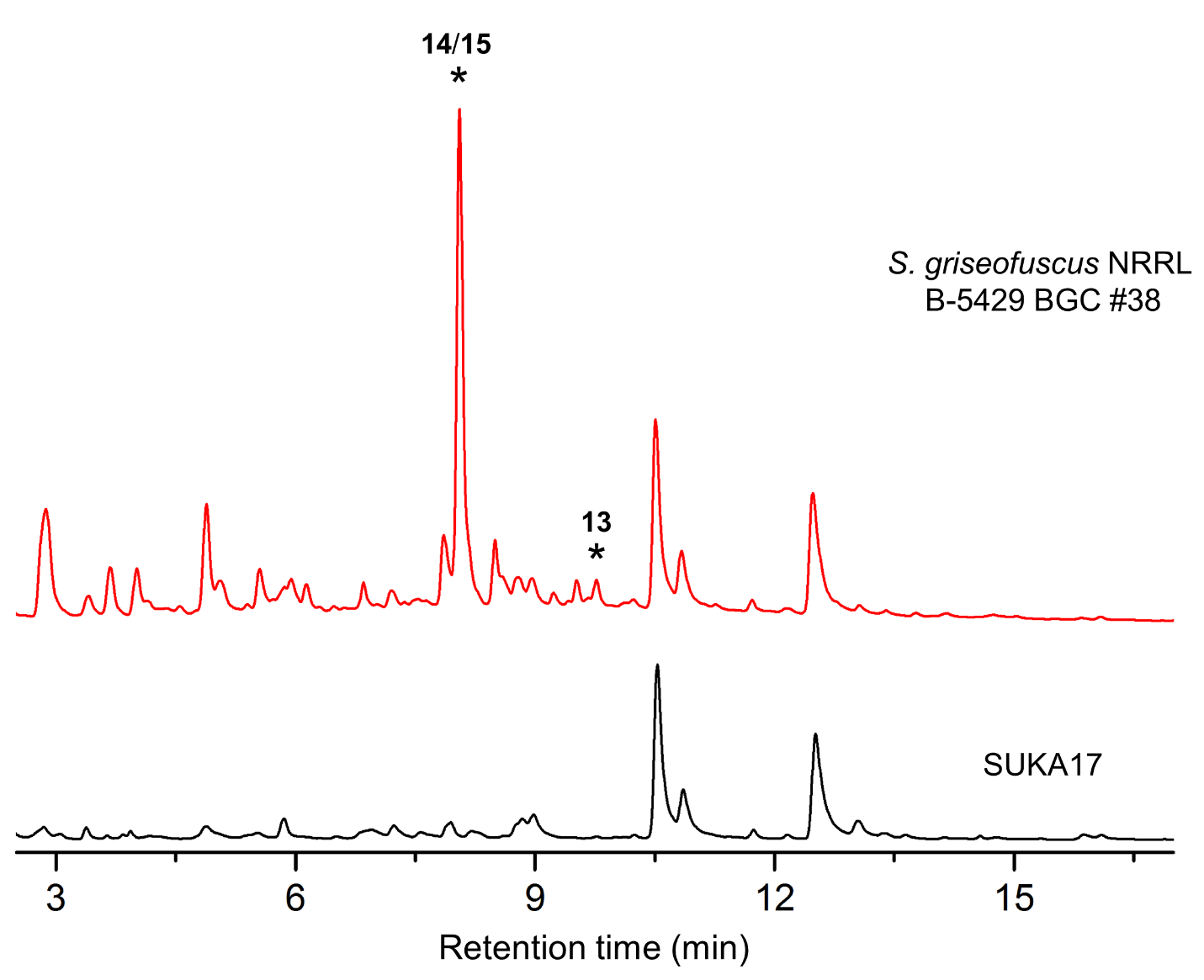


**Supplementary Fig. 15.** HPLC analysis of the crude extract from heterologous expression of BGC #38 from *S. griseofuscus* NRRL B-5429 in *S. avermitilis* SUKA17. Corresponding new peaks and their associated compounds are labeled with asterisks and numbers. **14**: citreodiol. **15**: *epi*-citreodiol. **13**: oxidative derivative of citreodiol.


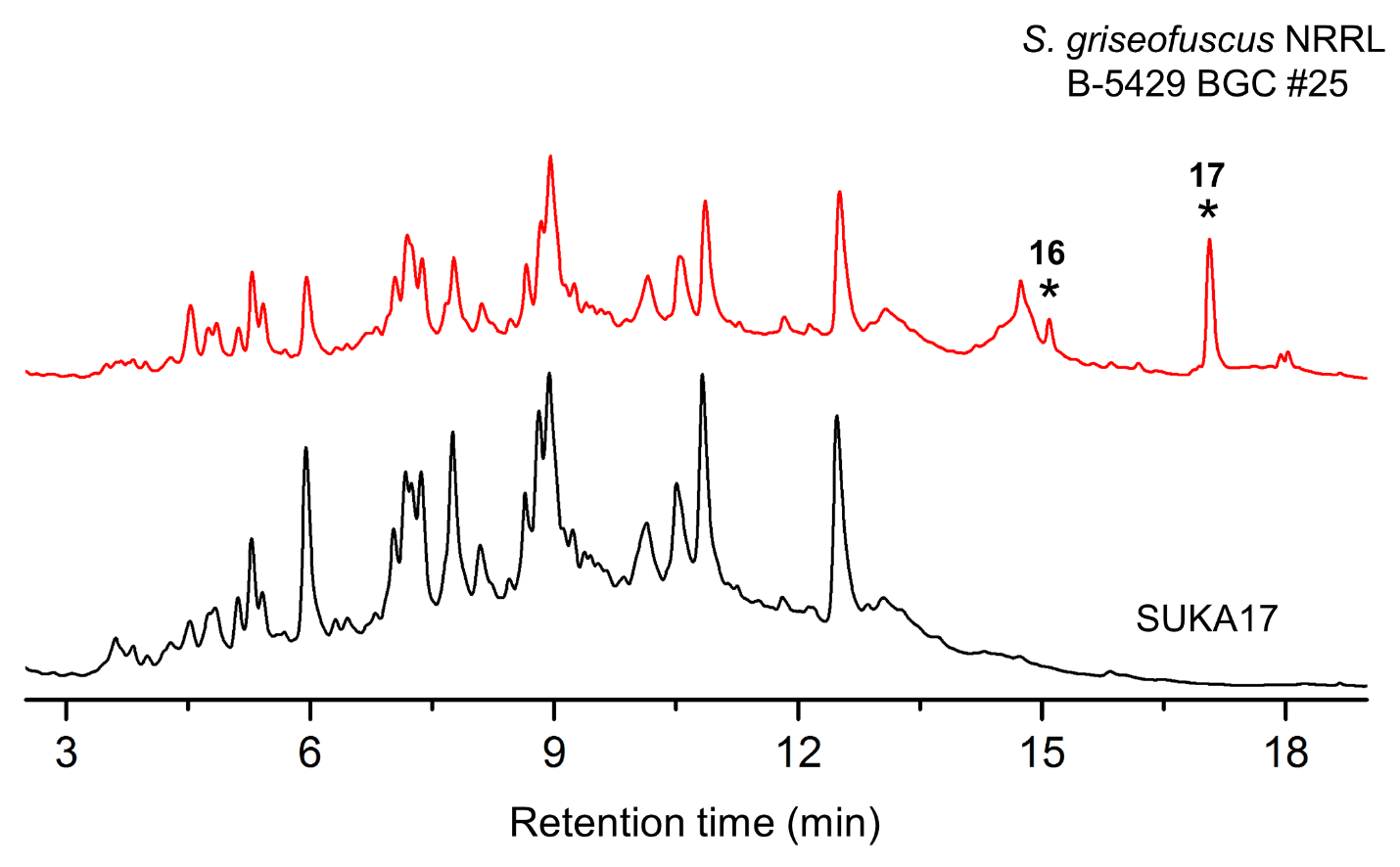


**Supplementary Fig. 16.** HPLC analysis of the crude extract from heterologous expression of BGC #25 from *S. griseofuscus* NRRL B-5429 in *S. avermitilis* SUKA17. Corresponding new peaks and their associated compounds are labeled with asterisks and numbers. **16**: allenomycin A. **17**: allenomycin B.


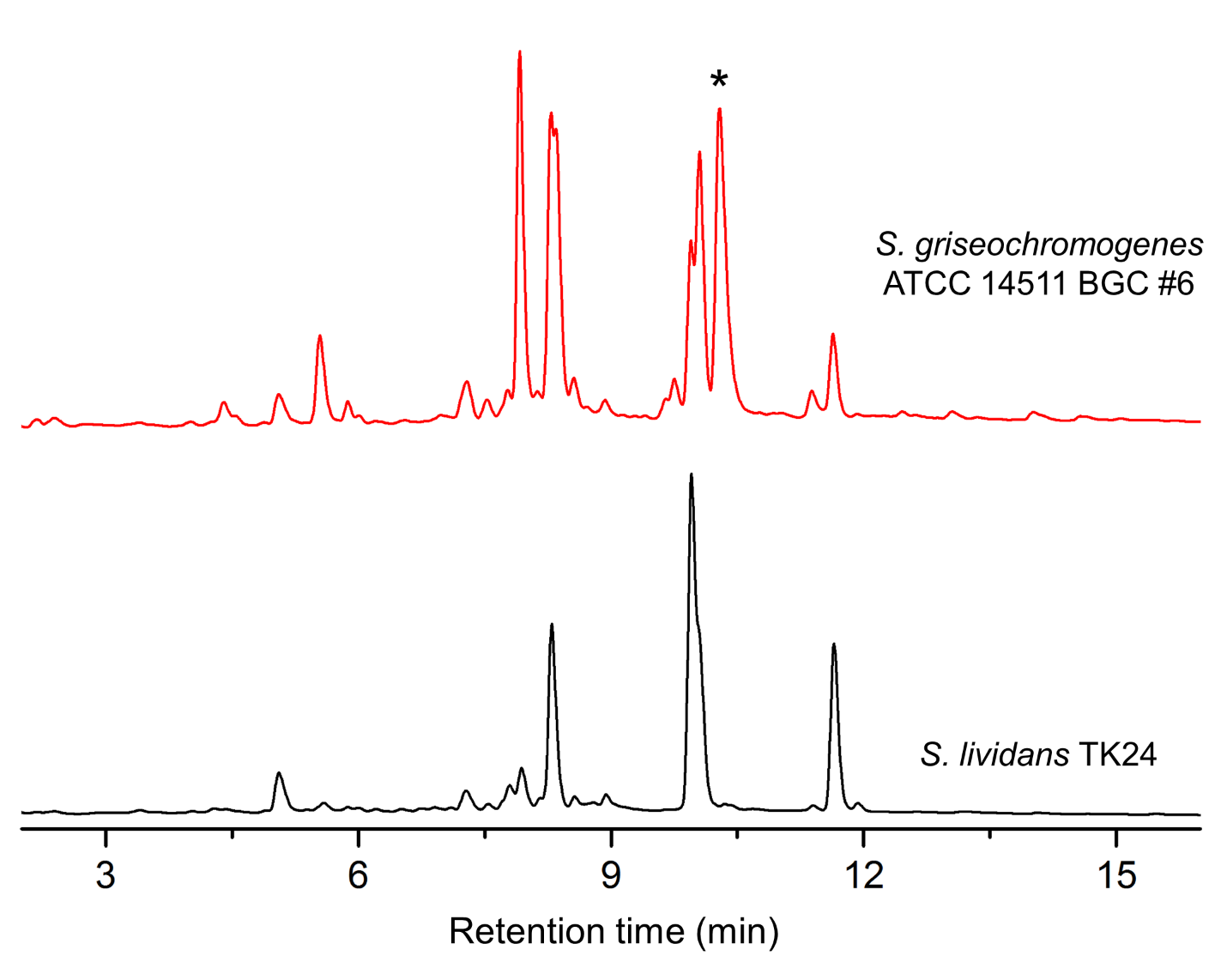


**Supplementary Fig. 17.** HPLC analysis of the crude extract from heterologous expression of BGC #6 from *S. griseochromogenes* ATCC 14511 in *S. lividans* TK24. Corresponding new peak is labeled with an asterisk.


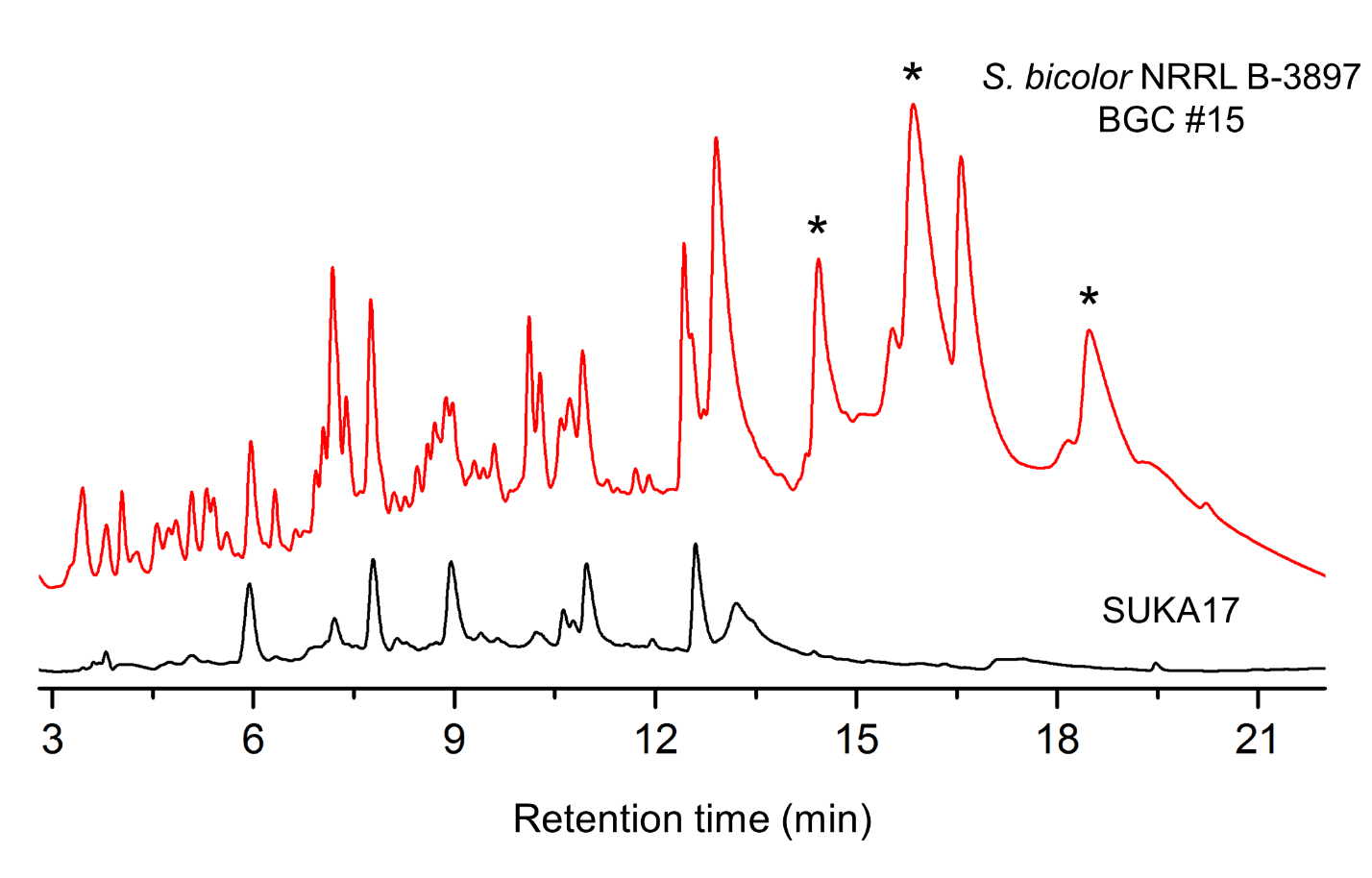


**Supplementary Fig. 18.** HPLC analysis of the crude extract from heterologous expression of BGC #15 from *S. bicolor* NRRL B-3897 in *S. avermitilis* SUKA17. Corresponding new peaks are labeled with an asterisk.


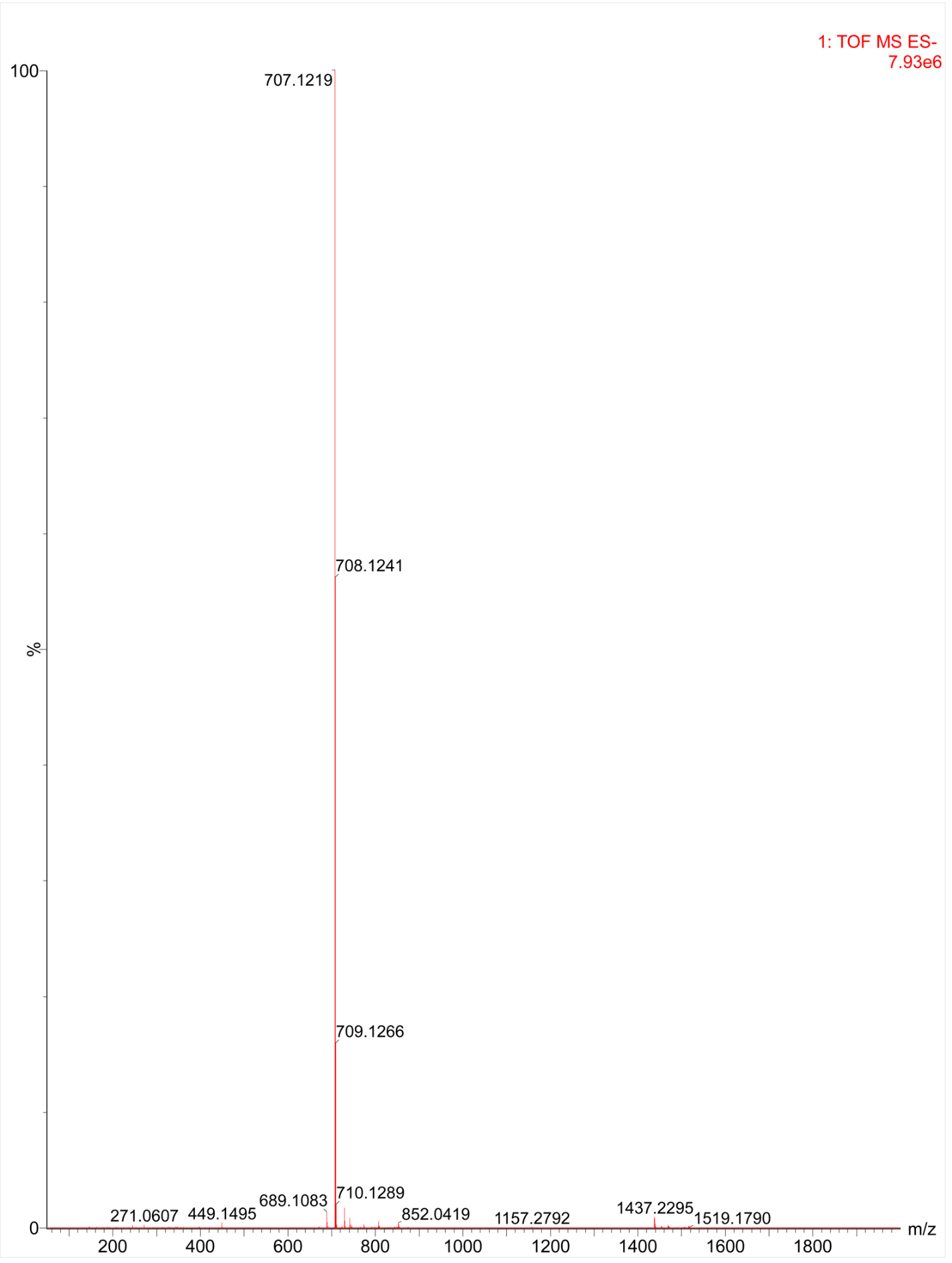


**Supplementary Fig. 19.** HRESIMS spectrum of bipentaromycin A (**1**).


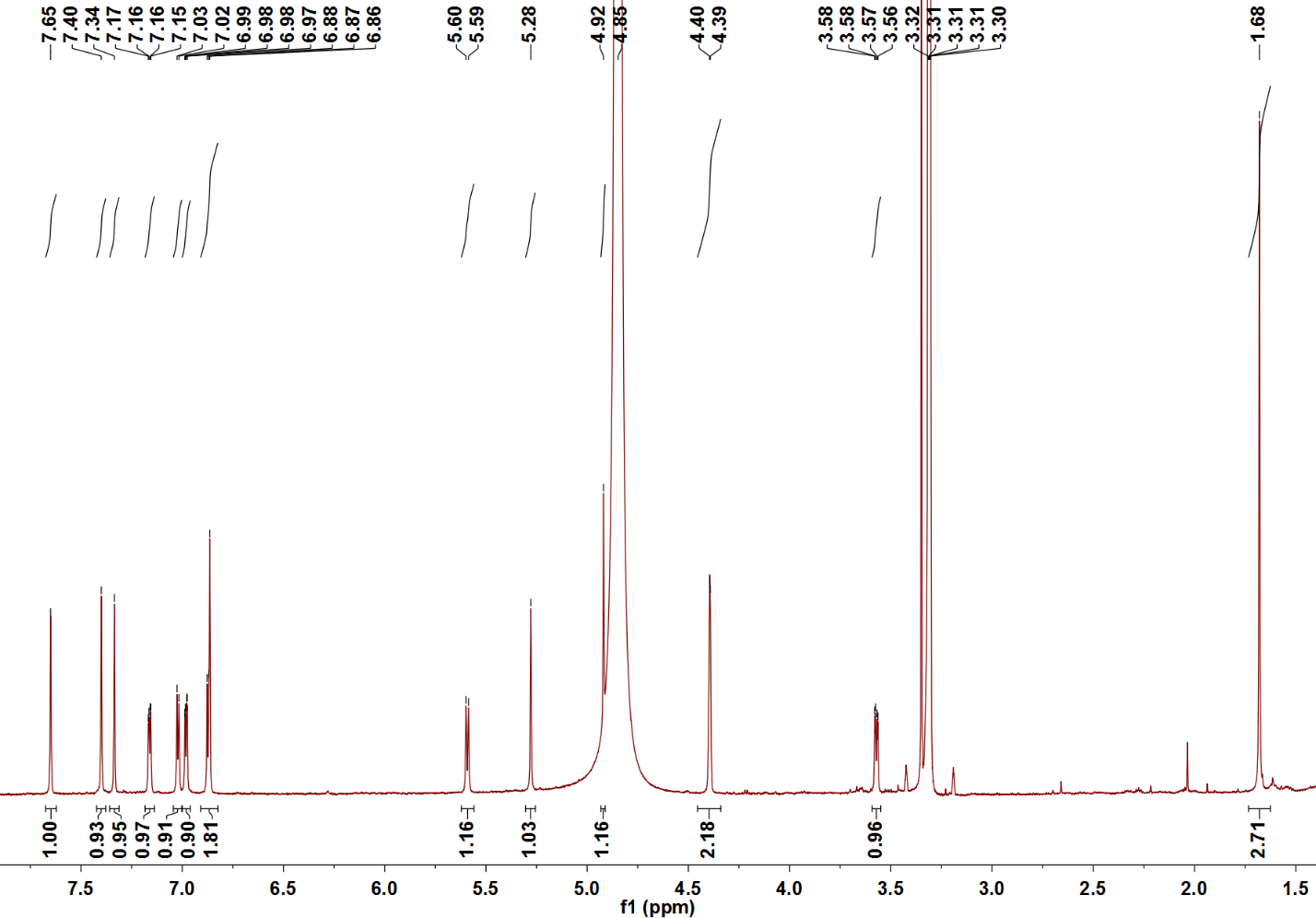


**Supplementary Fig. 20.** ^1^H NMR spectrum of bipentaromycin A (**1**) in CD_3_OD.


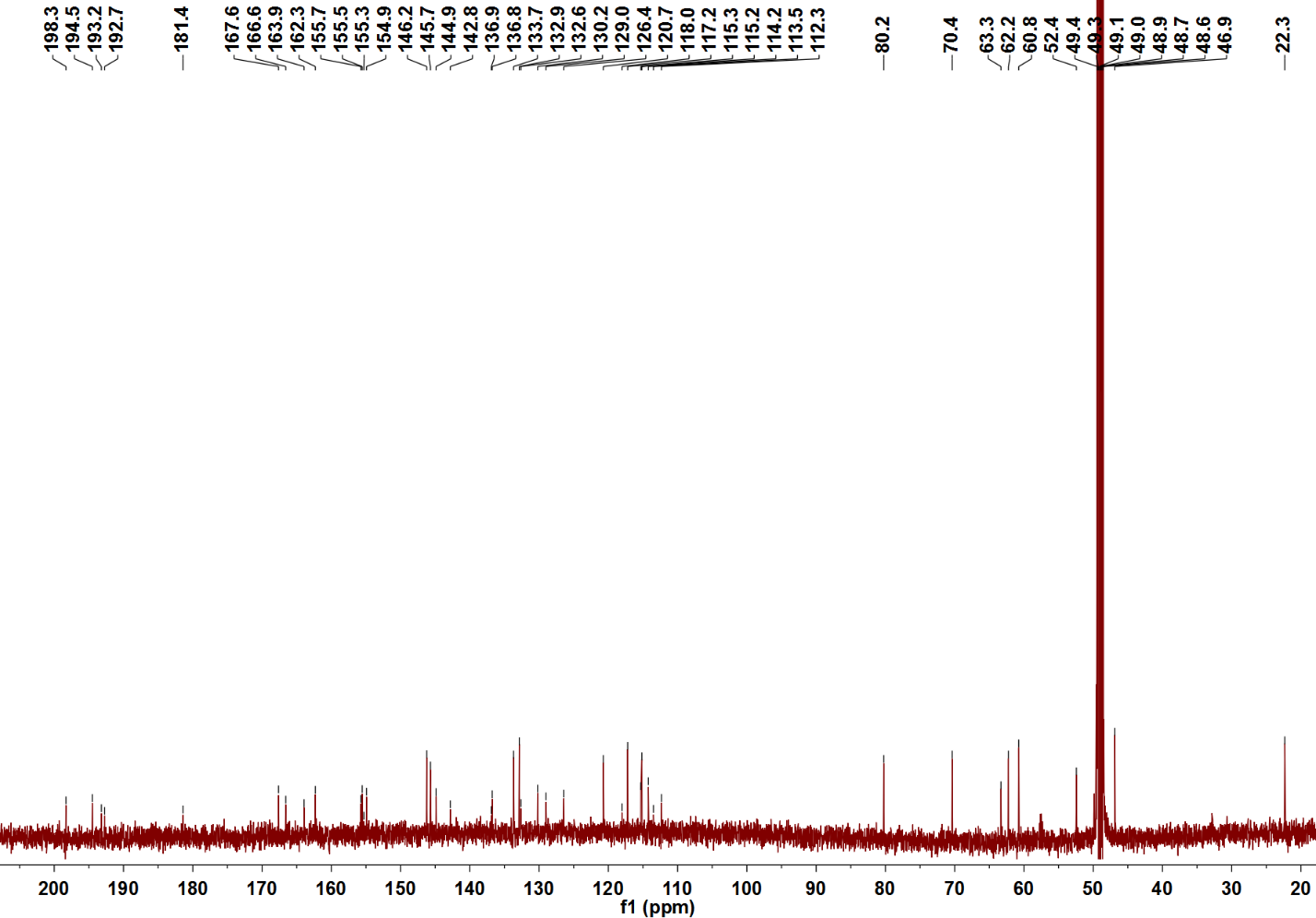


**Supplementary Fig. 21.** ^13^C NMR spectrum of bipentaromycin A (**1**) in CD_3_OD.


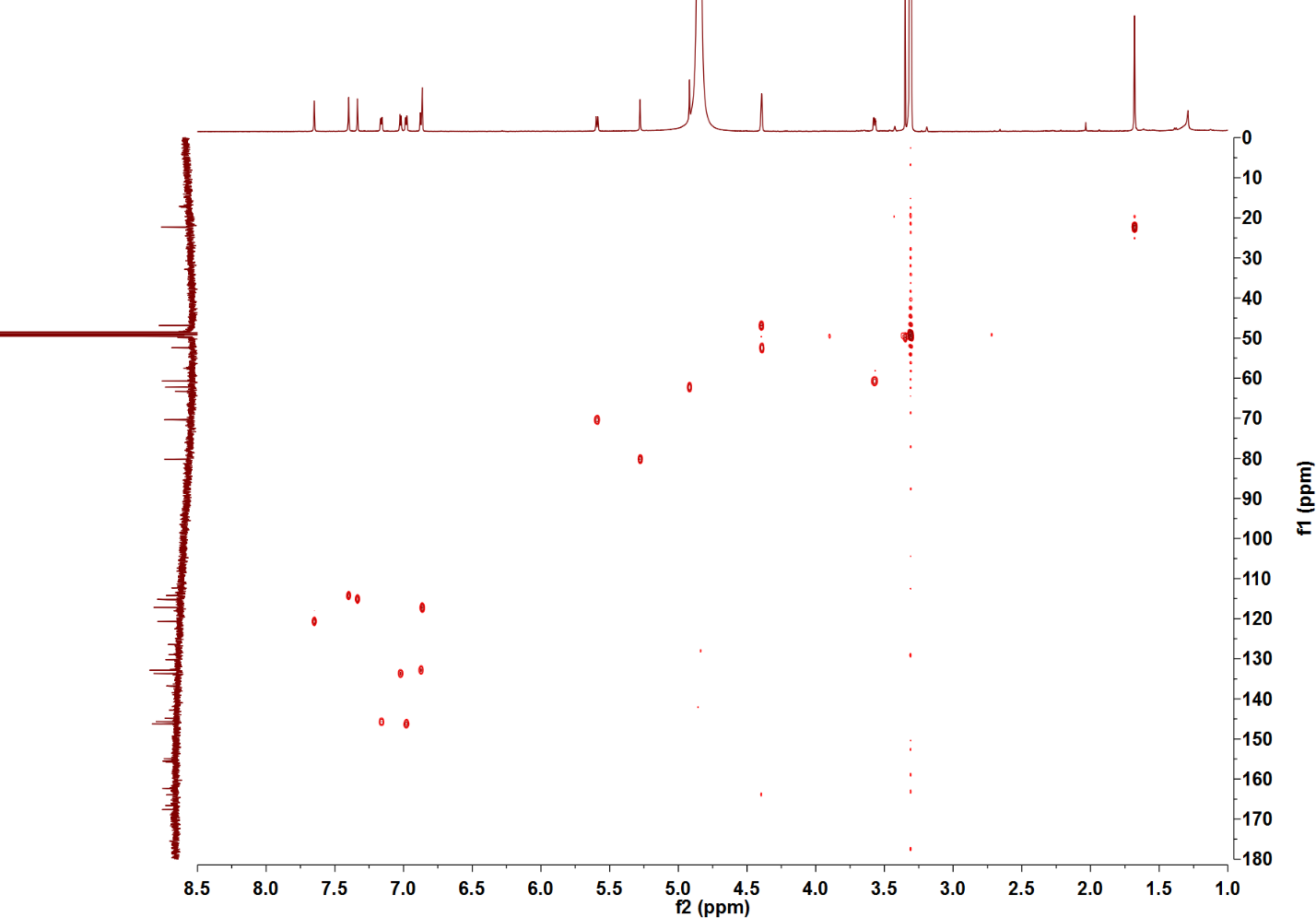


**Supplementary Fig. 22.** HSQC spectrum of bipentaromycin A (**1**) in CD_3_OD.


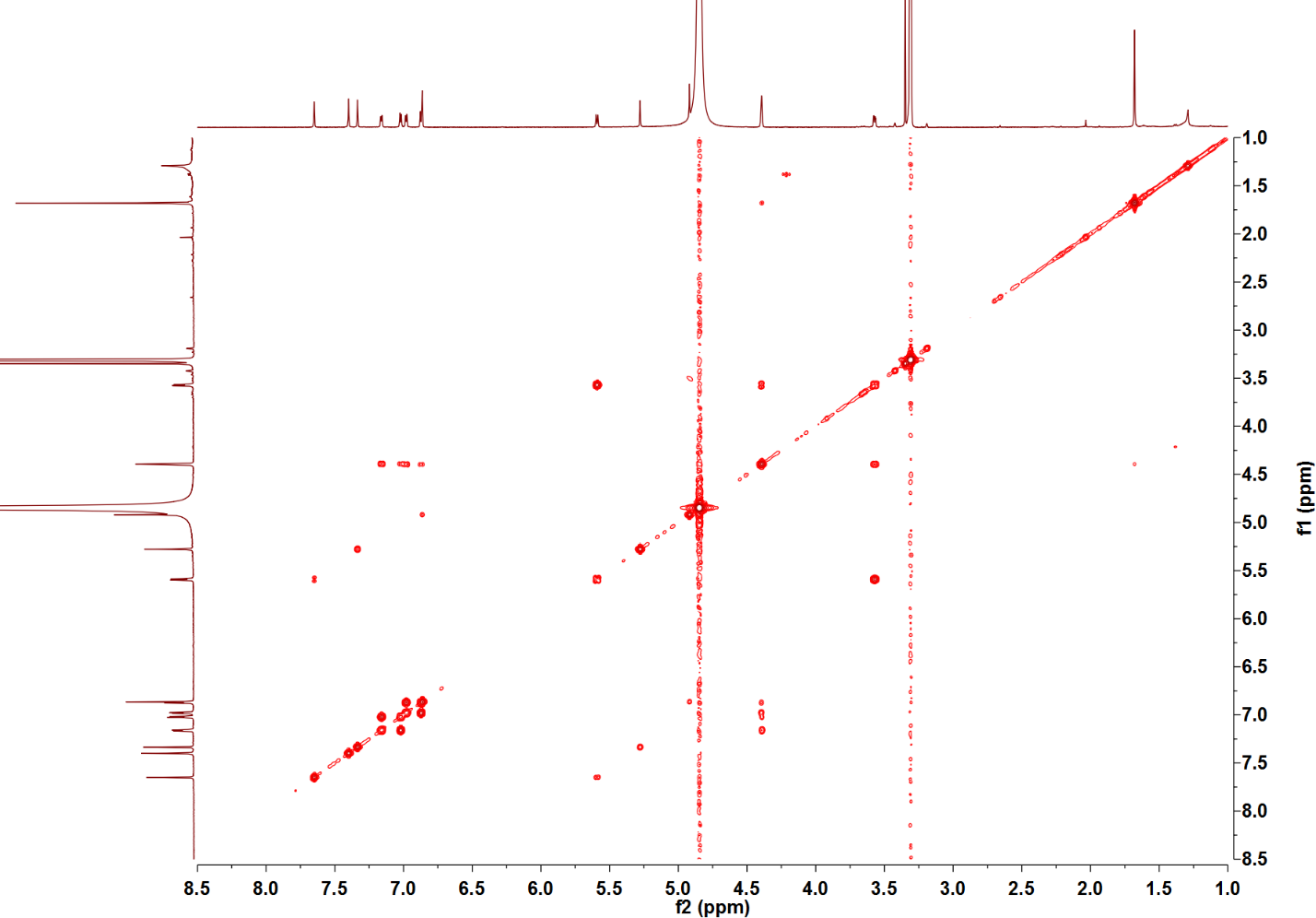

**Supplementary Fig. 23.** COSY spectrum of bipentaromycin A (**1**) in CD_3_OD.


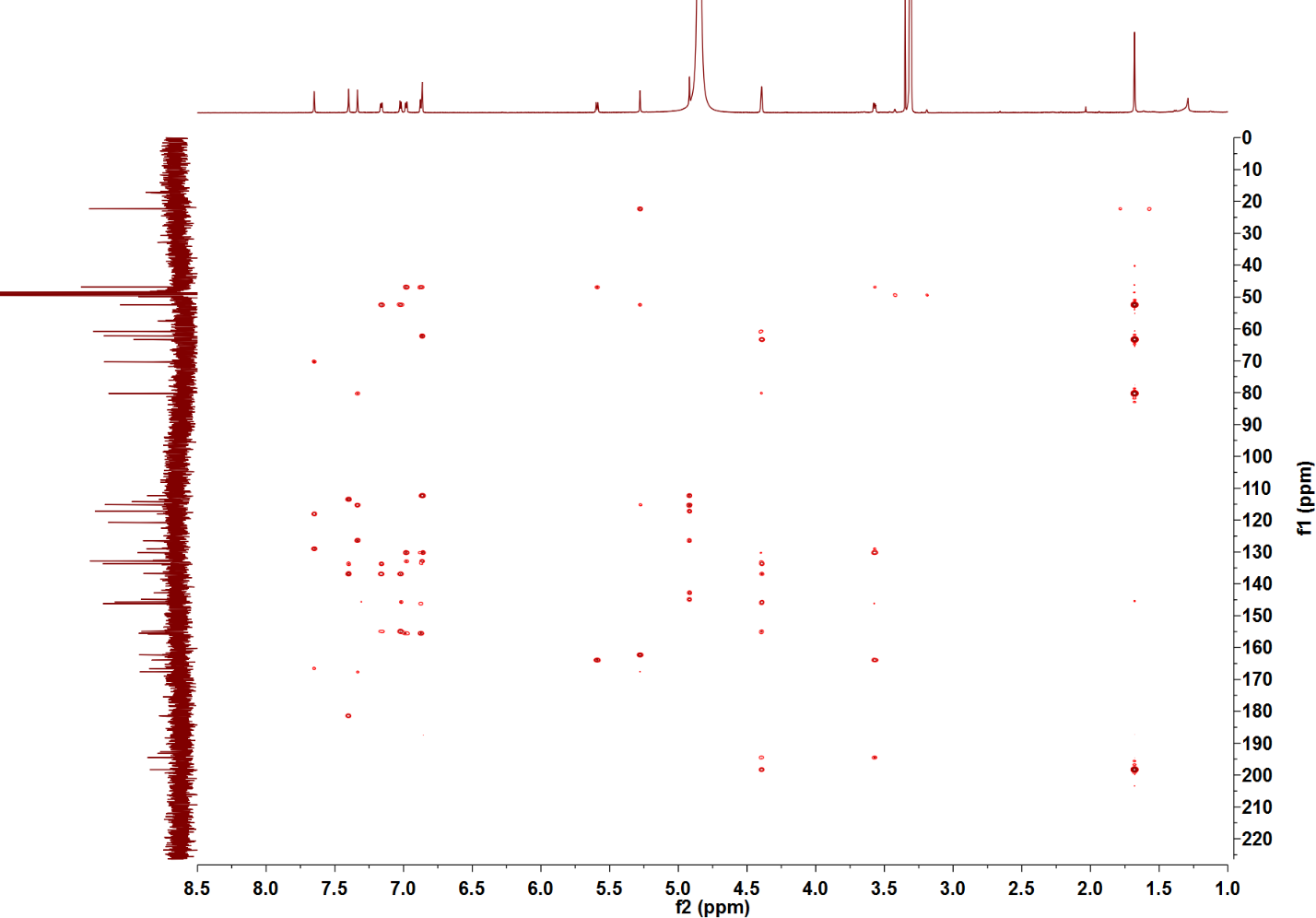

**Supplementary Fig. 24.** HMBC spectrum of bipentaromycin A (**1**) in CD_3_OD.


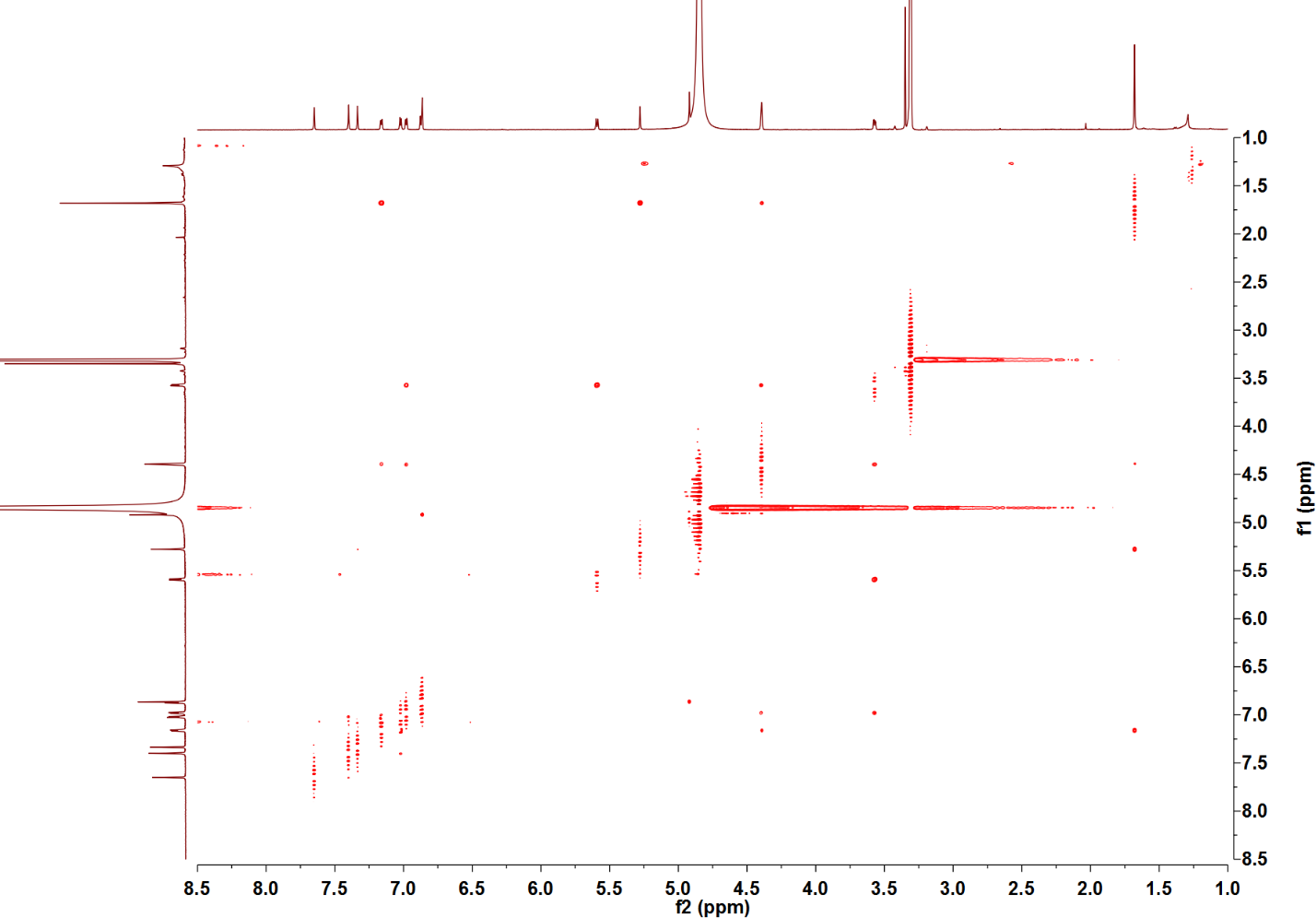

**Supplementary Fig. 25.** NOESY spectrum of bipentaromycin A (**1**) in CD_3_OD.


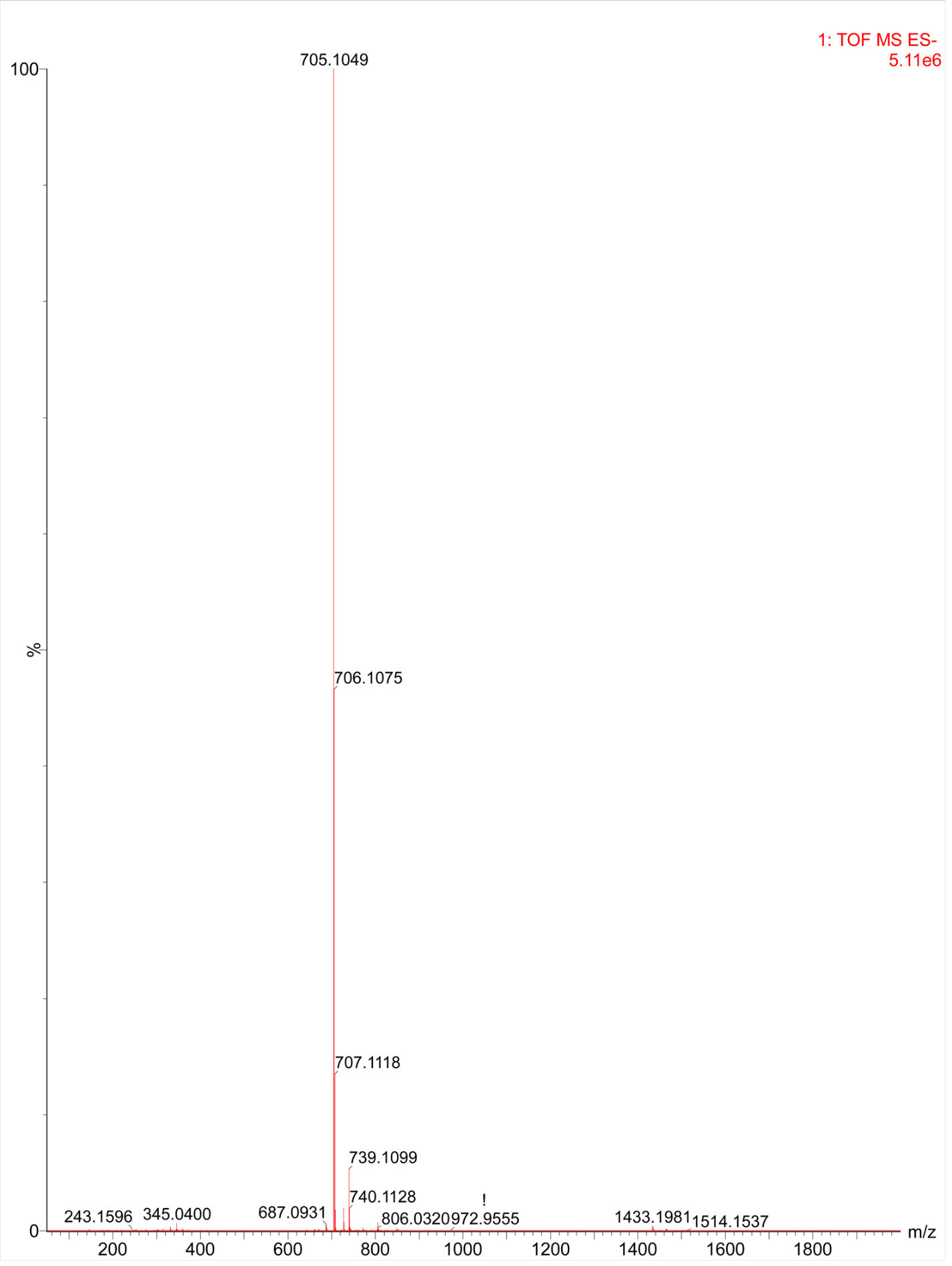


**Supplementary Fig. 26.** HRESIMS spectrum of bipentaromycin B (**2**).


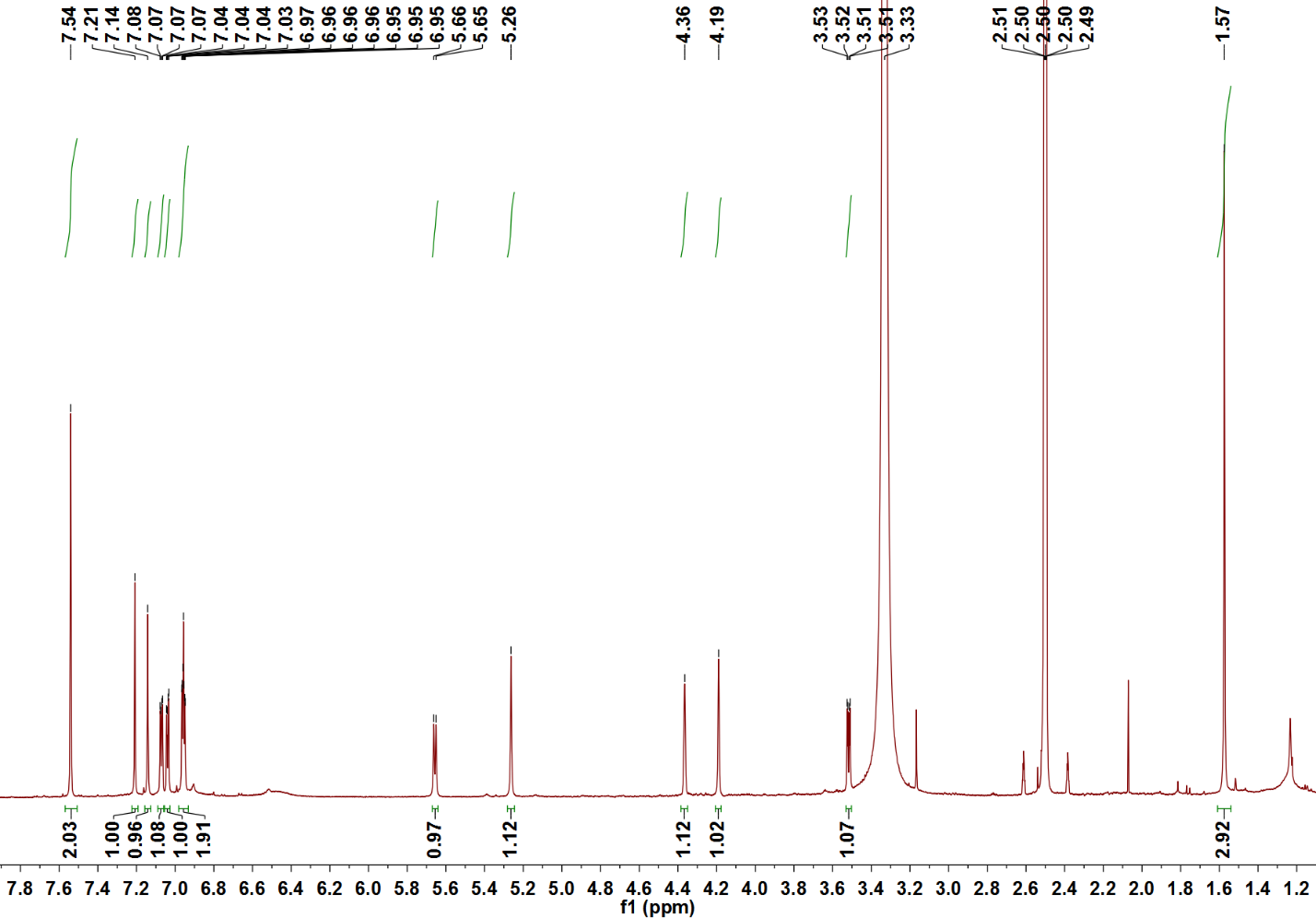


**Supplementary Fig. 27.** ^1^H NMR spectrum of bipentaromycin B (**2**) in DMSO-*d*_6_.


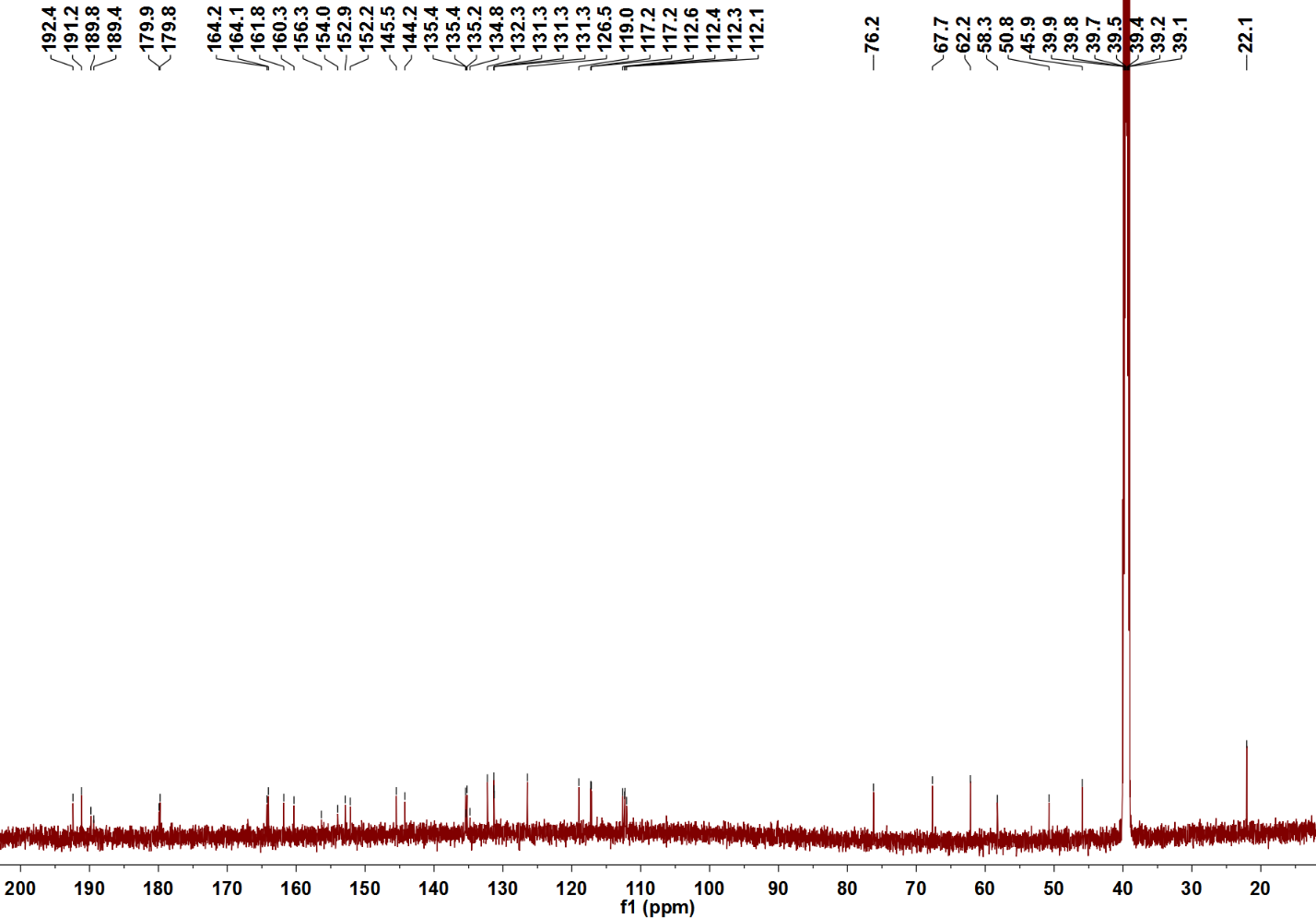


**Supplementary Fig. 28.** ^13^C NMR spectrum of bipentaromycin B (**2**) in DMSO-*d*_6_.


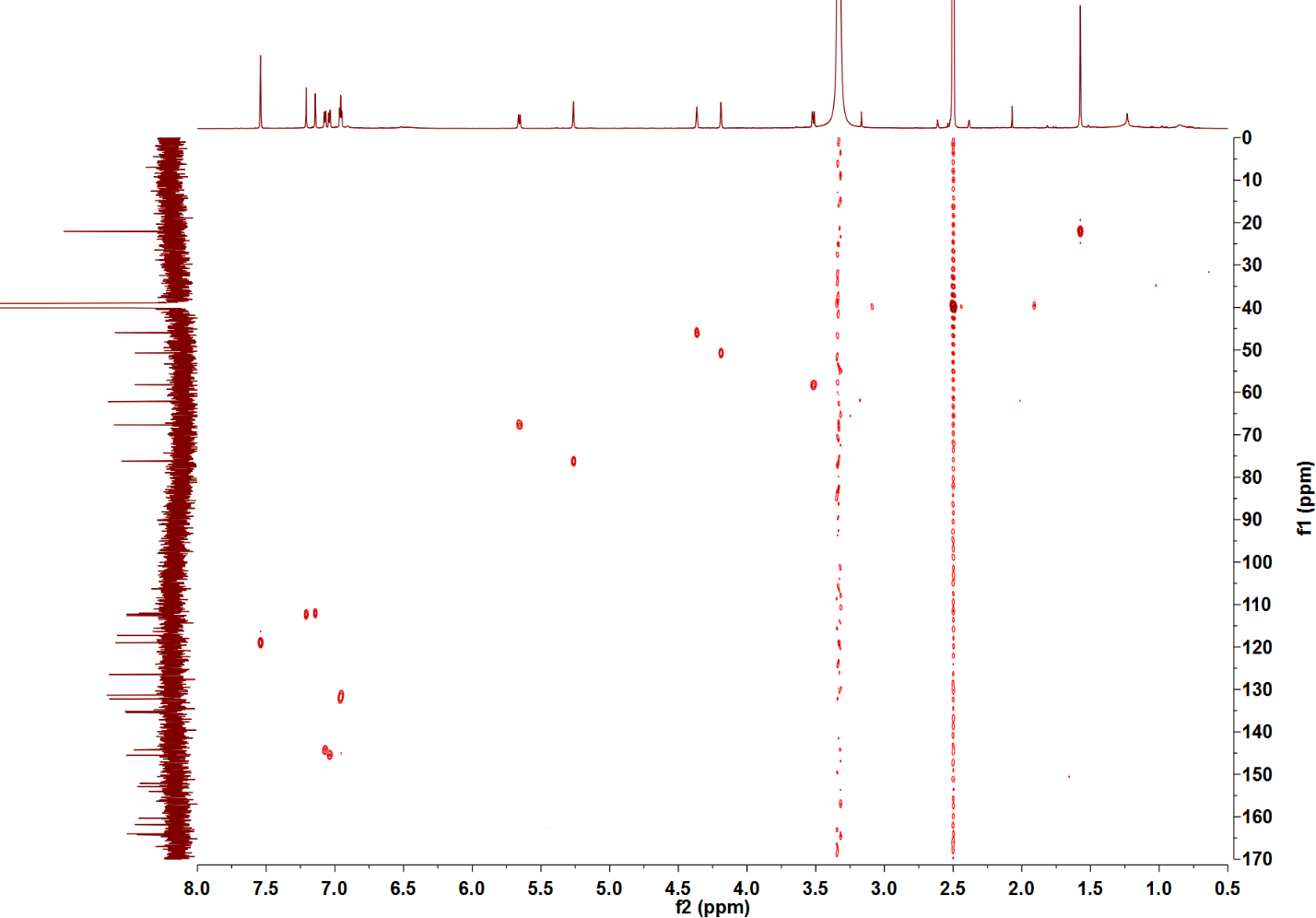
 **Supplementary Fig. 29.** HSQC spectrum of bipentaromycin B (**2**) in DMSO-*d*_6_.


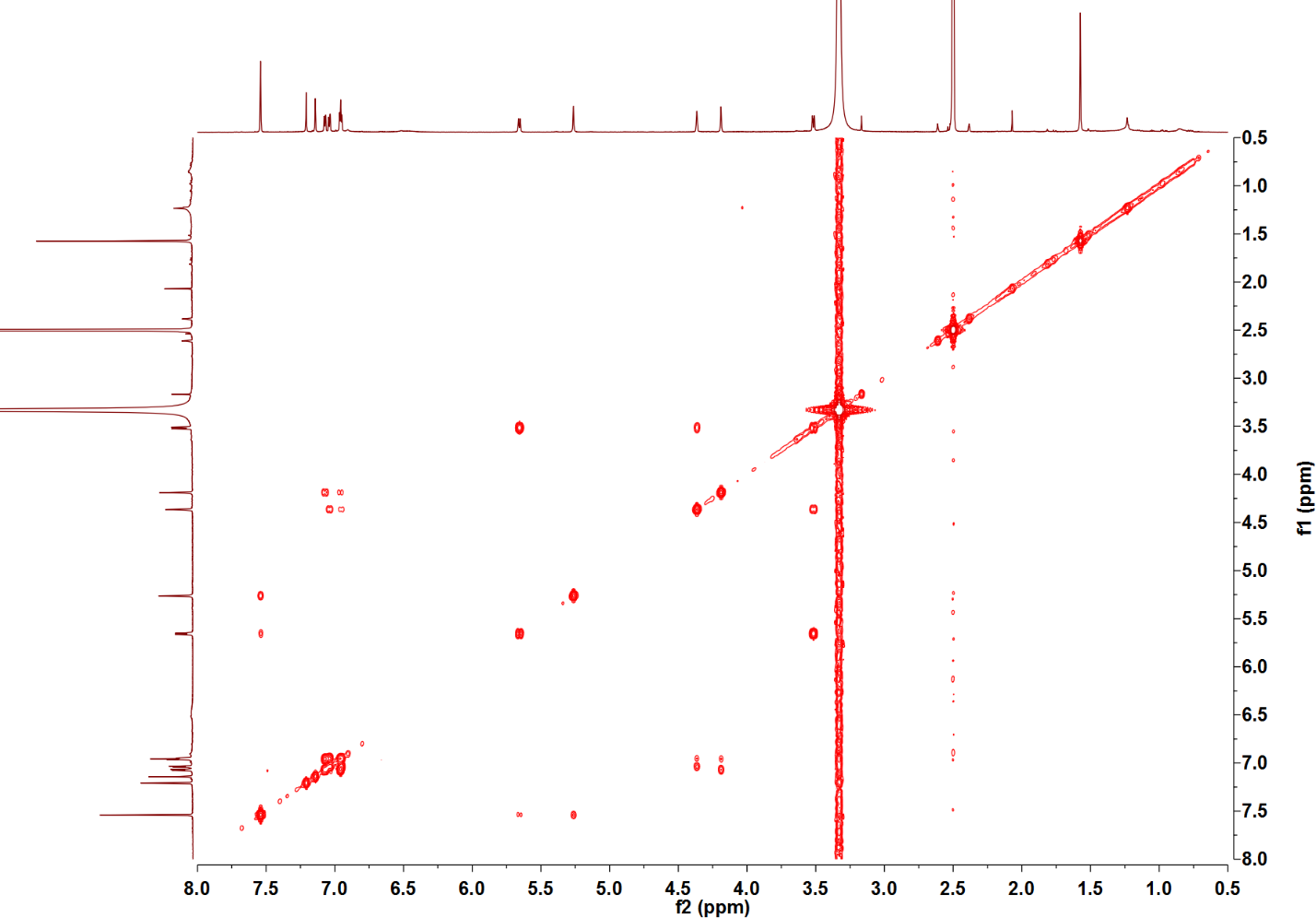

**Supplementary Fig. 30.** COSY spectrum of bipentaromycin B (**2**) in DMSO-*d*_6_.


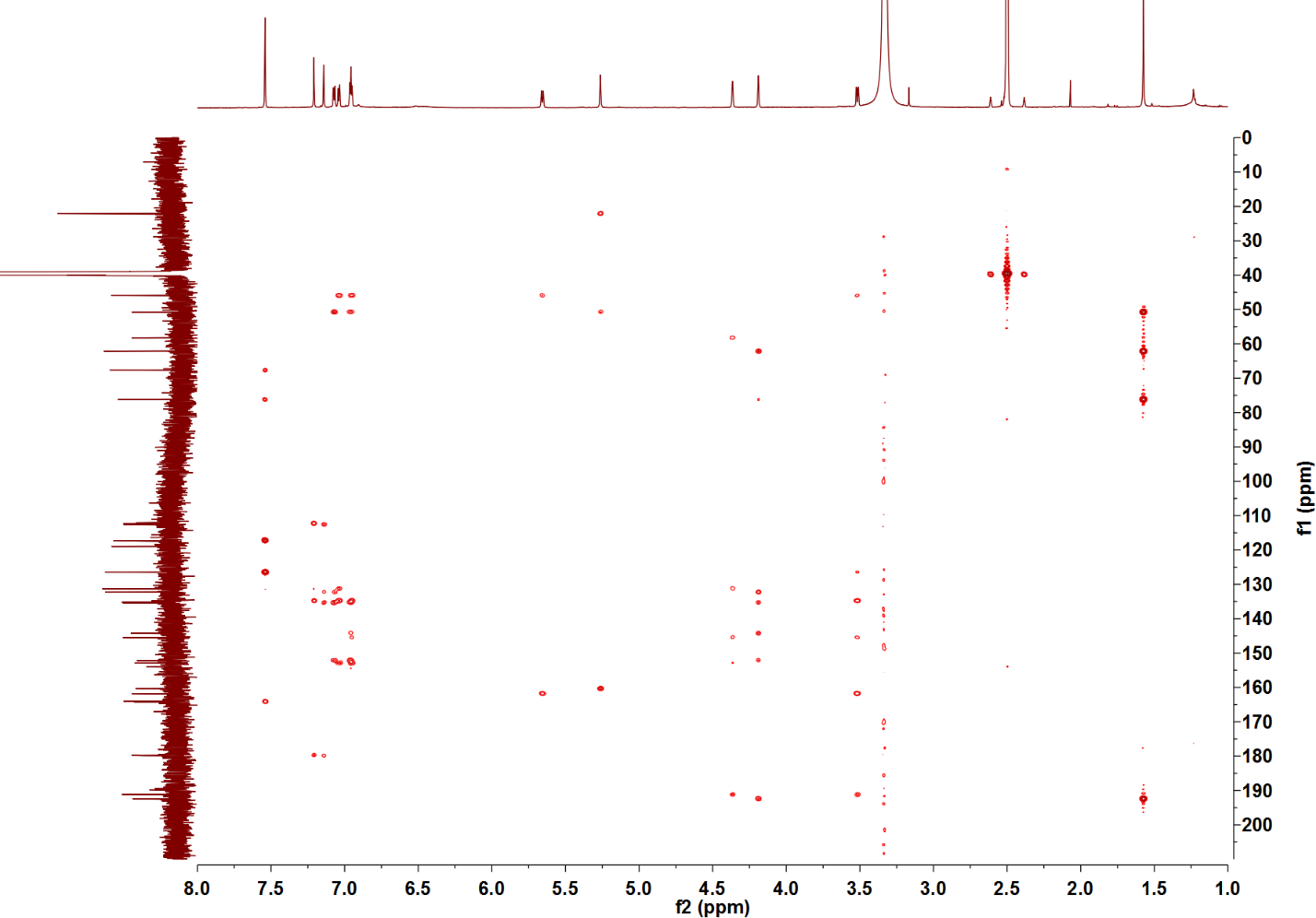

**Supplementary Fig. 31.** HMBC spectrum of bipentaromycin B (**2**) in DMSO-*d*_6_.


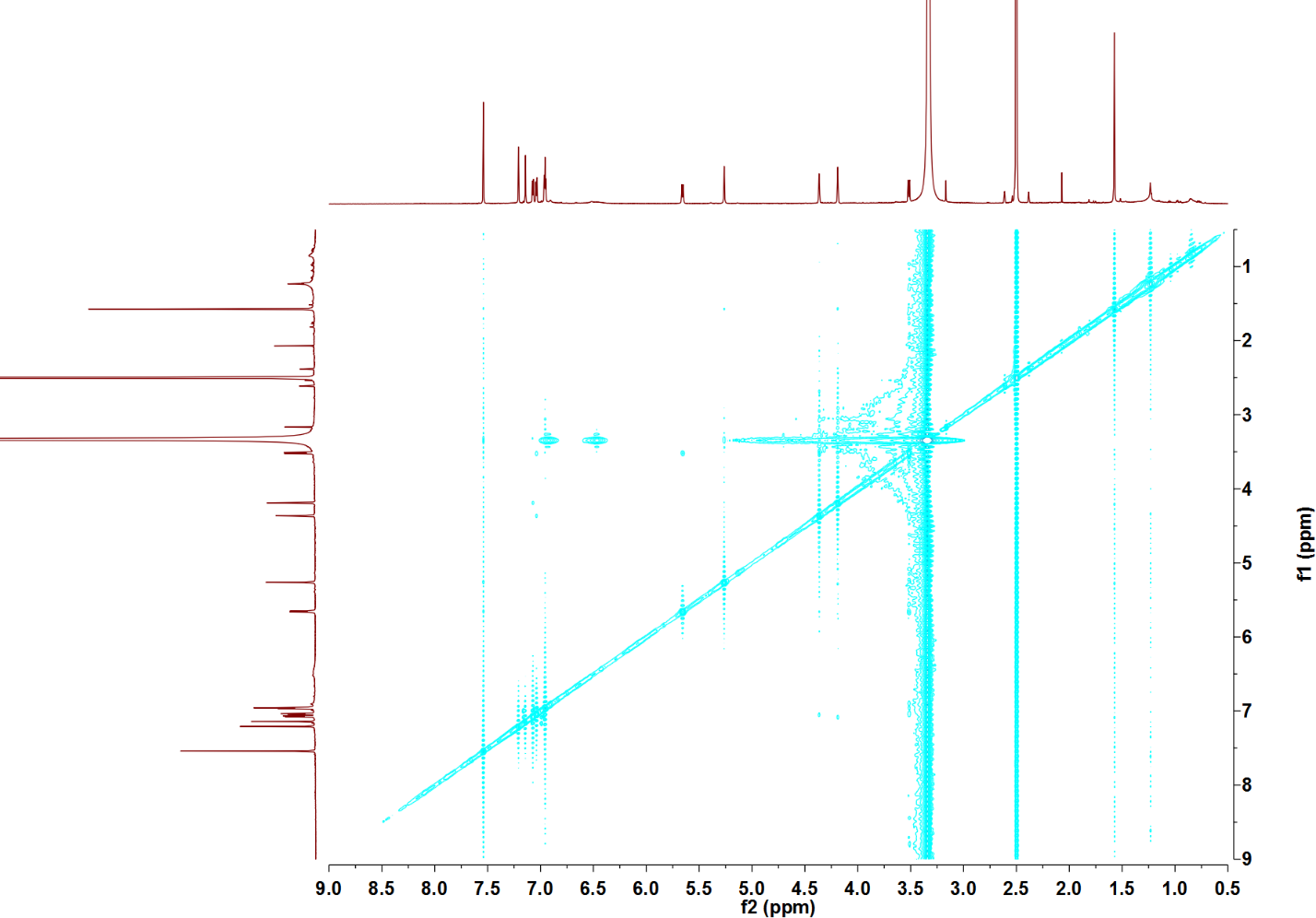


**Supplementary Fig. 32.** NOESY spectrum of bipentaromycin B (**2**) in DMSO-*d*_6_.


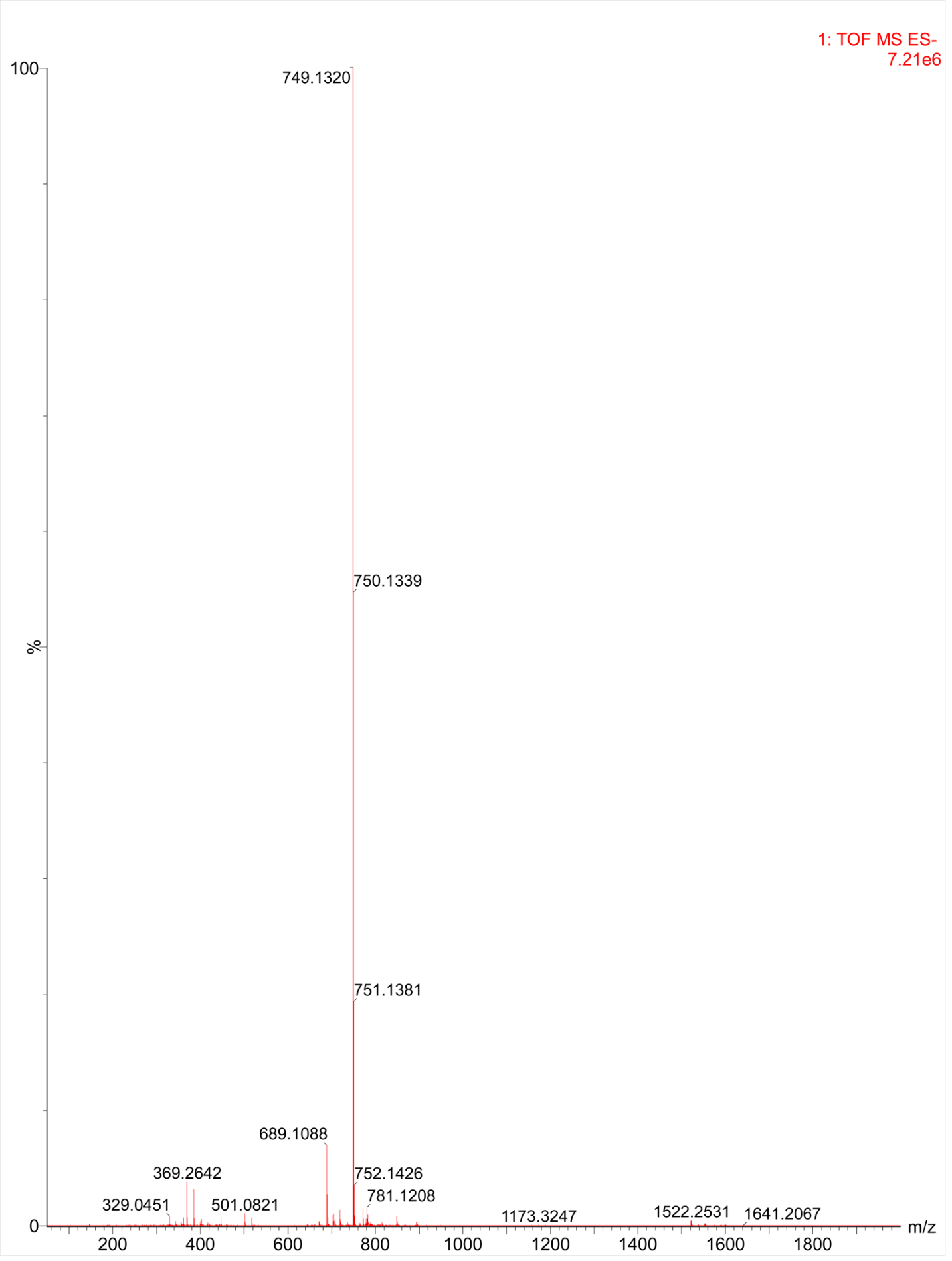


**Supplementary Fig. 33.** HRESIMS spectrum of bipentaromycin C (**3**).


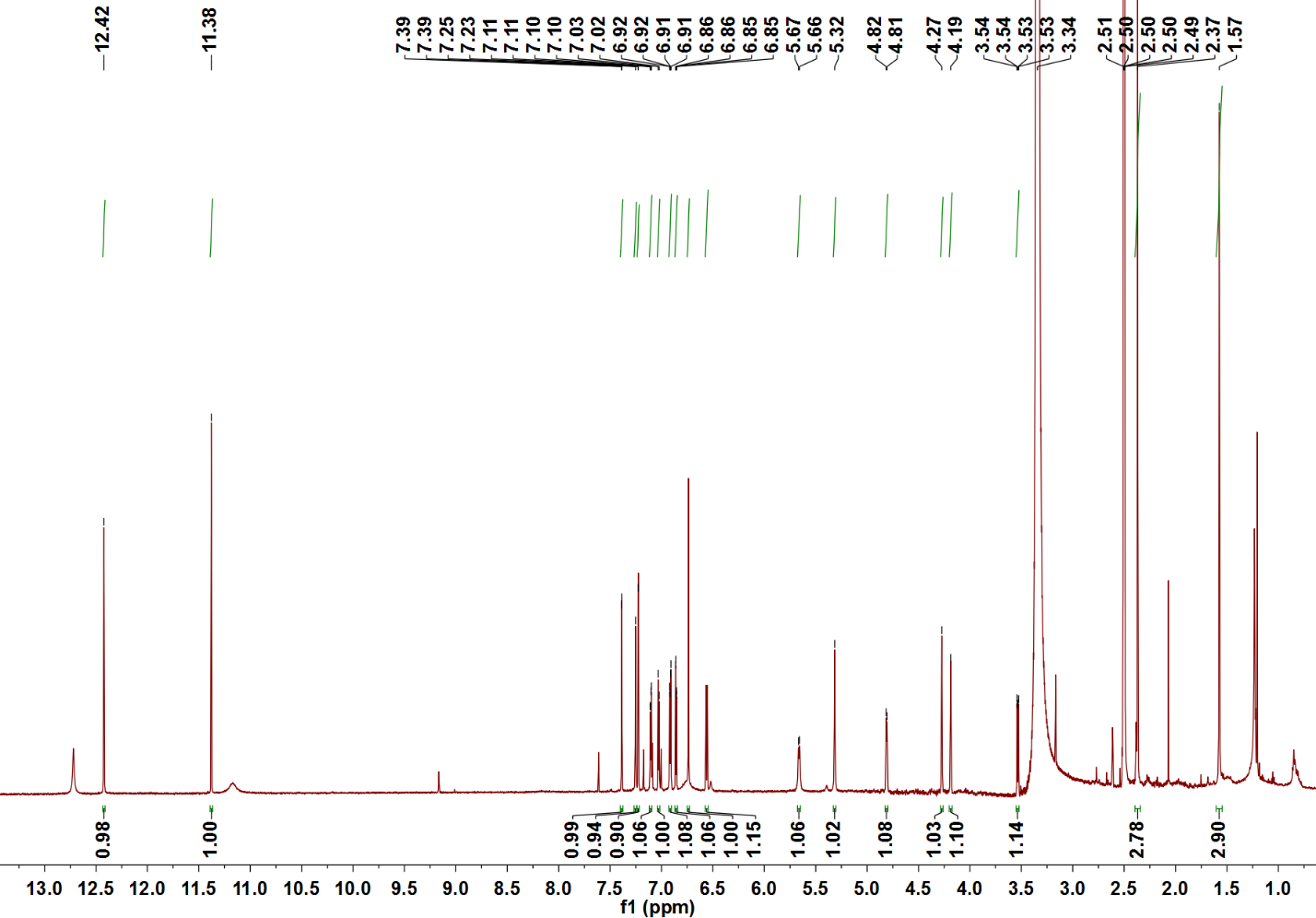


**Supplementary Fig. 34.** ^1^H NMR spectrum of bipentaromycin C (**3**) in DMSO-*d*_6_.


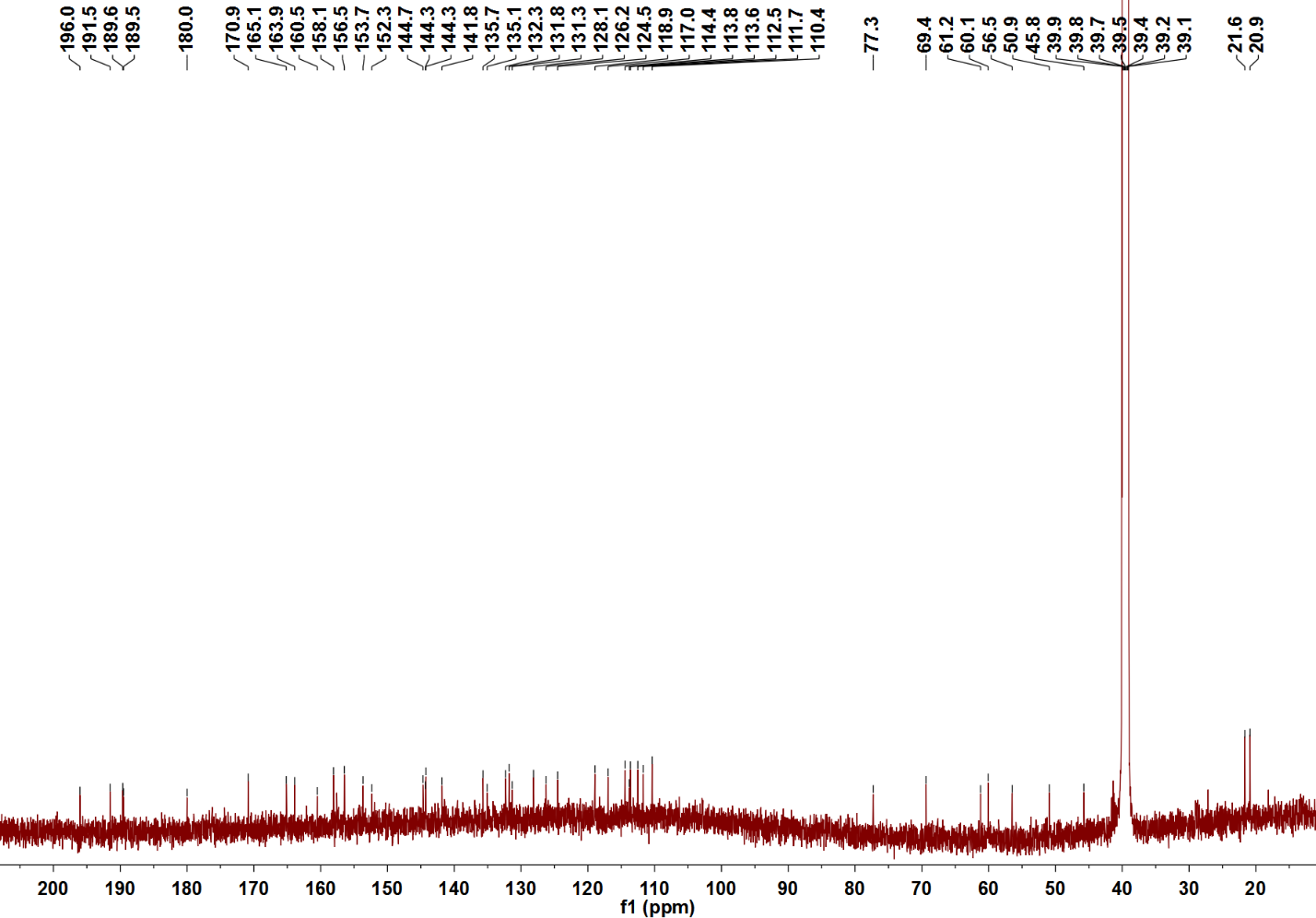


**Supplementary Fig. 35.** ^13^C NMR spectrum of bipentaromycin C (**3**) in DMSO-*d*_6_.


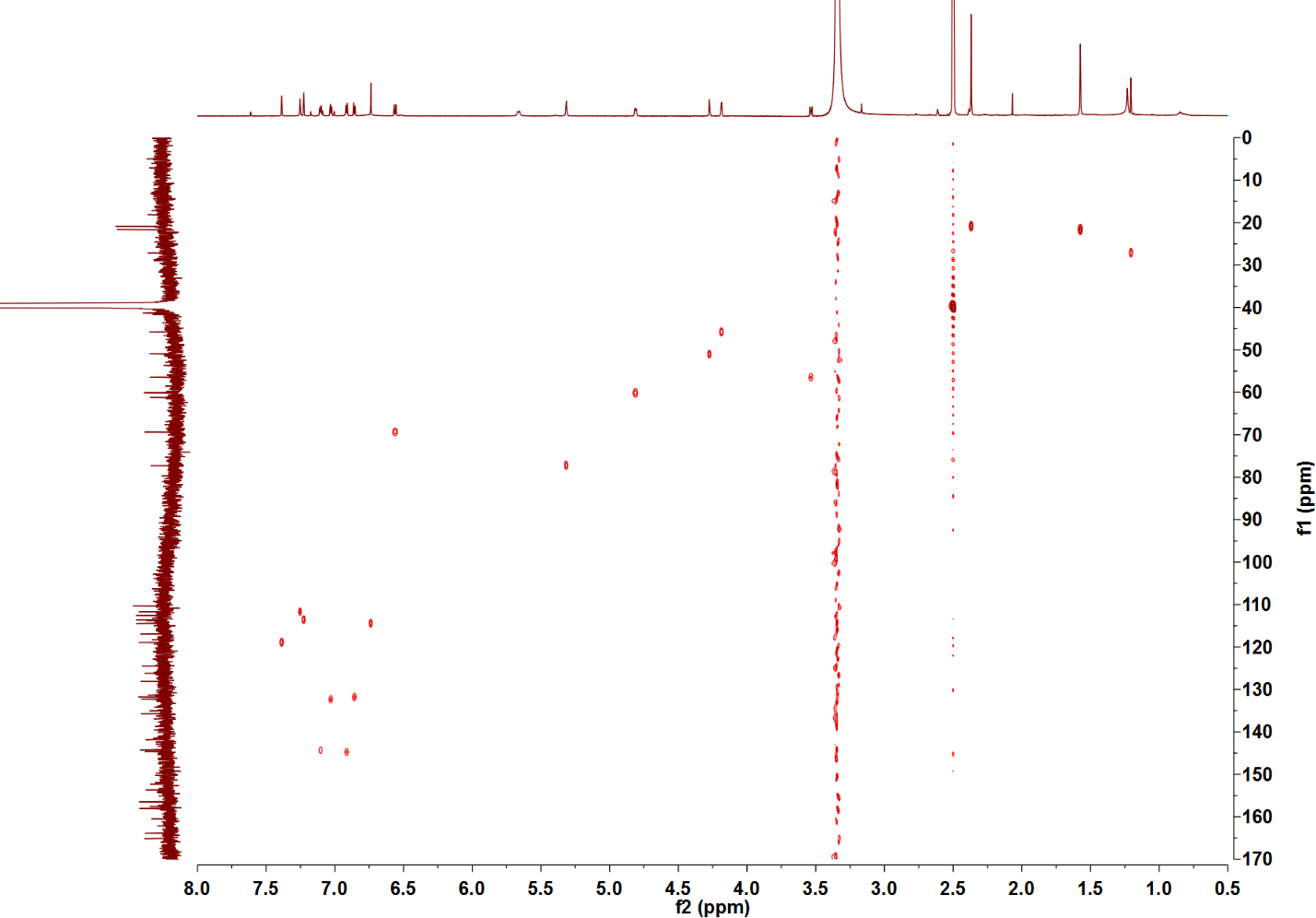


**Supplementary Fig. 36.** HSQC spectrum of bipentaromycin C (**3**) in DMSO-*d*_6_.


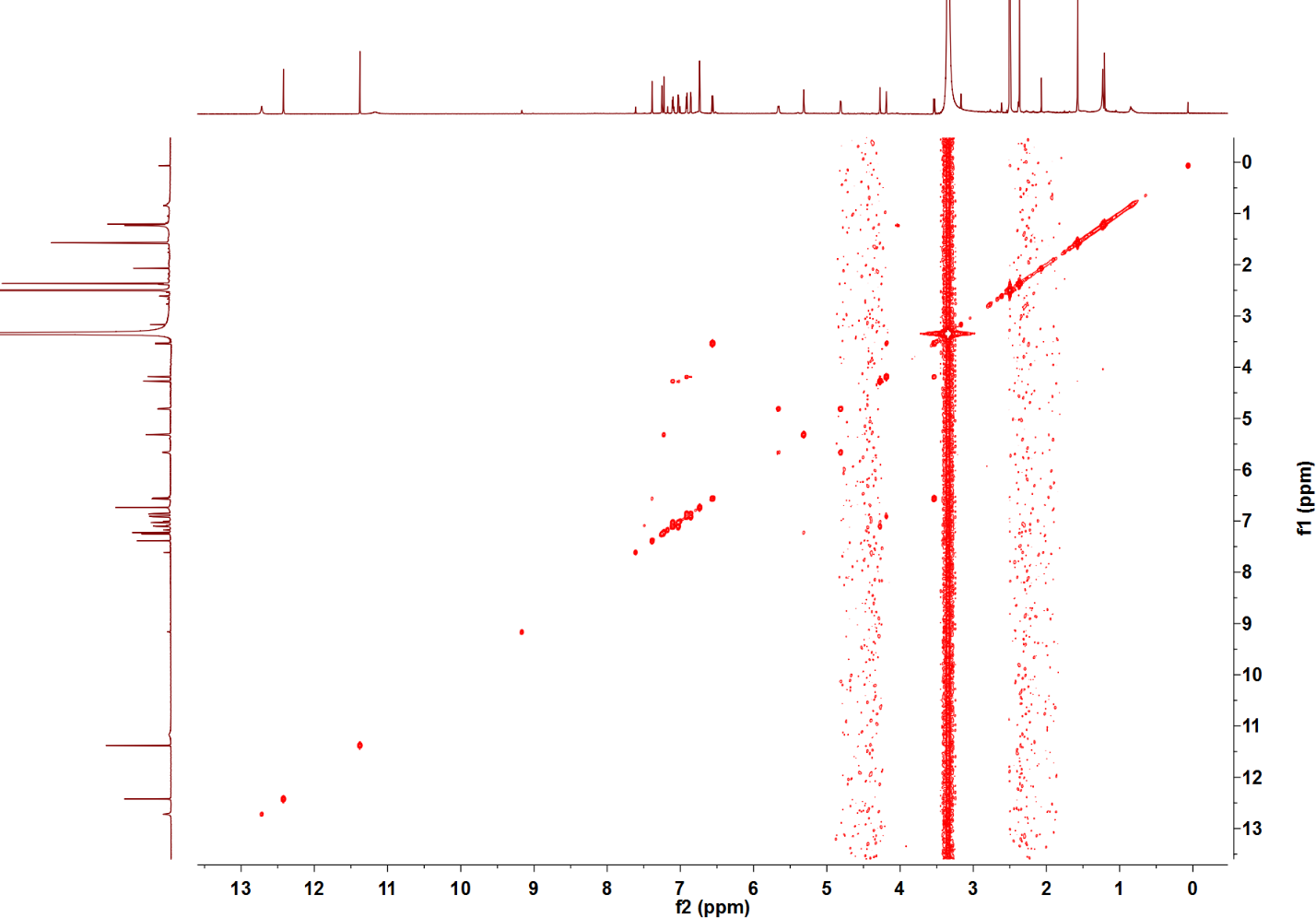

**Supplementary Fig. 37.** COSY spectrum of bipentaromycin C (**3**) in DMSO-*d*_6_.


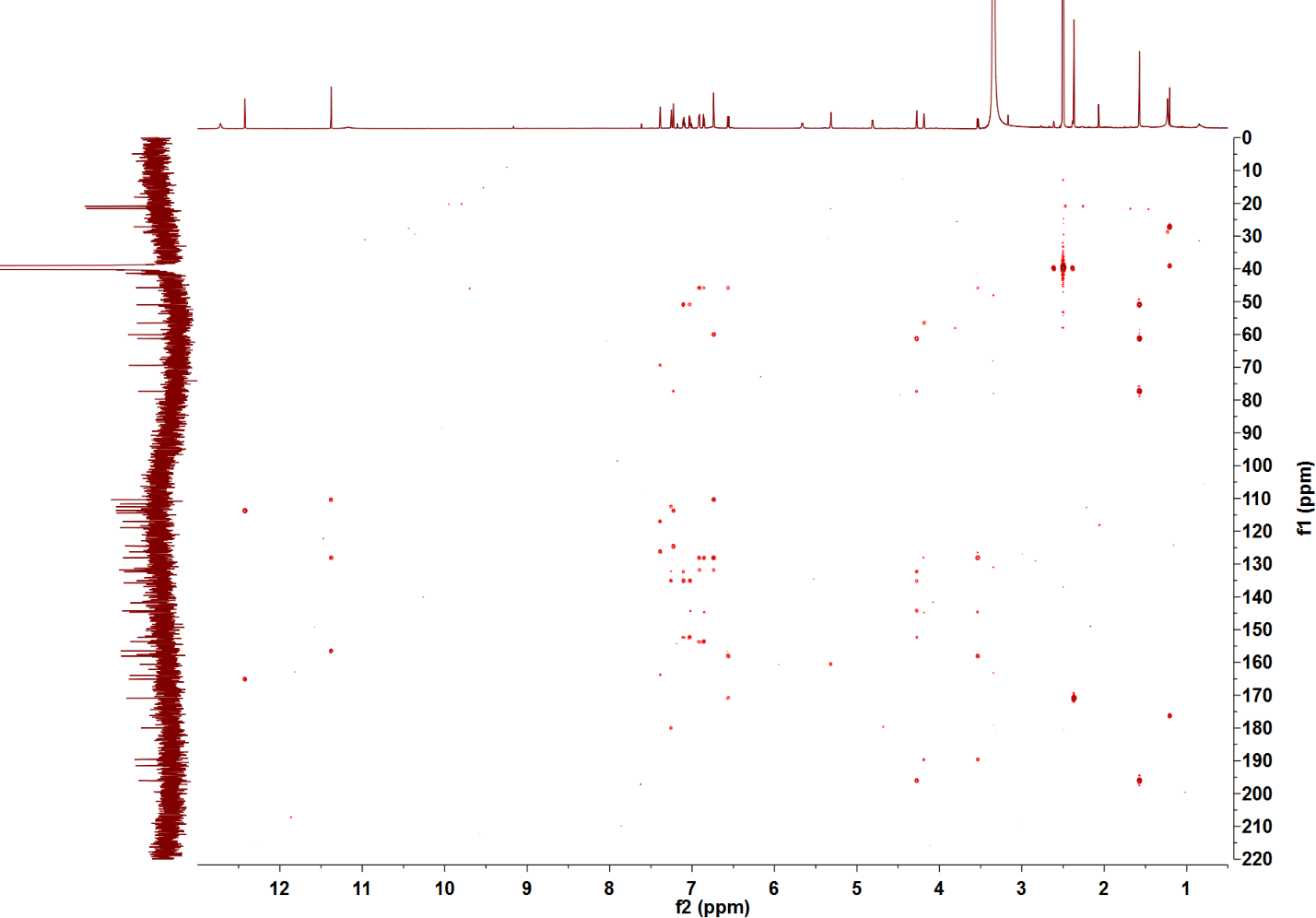

**Supplementary Fig. 38.** HMBC spectrum of bipentaromycin C (**3**) in DMSO-*d*_6_.


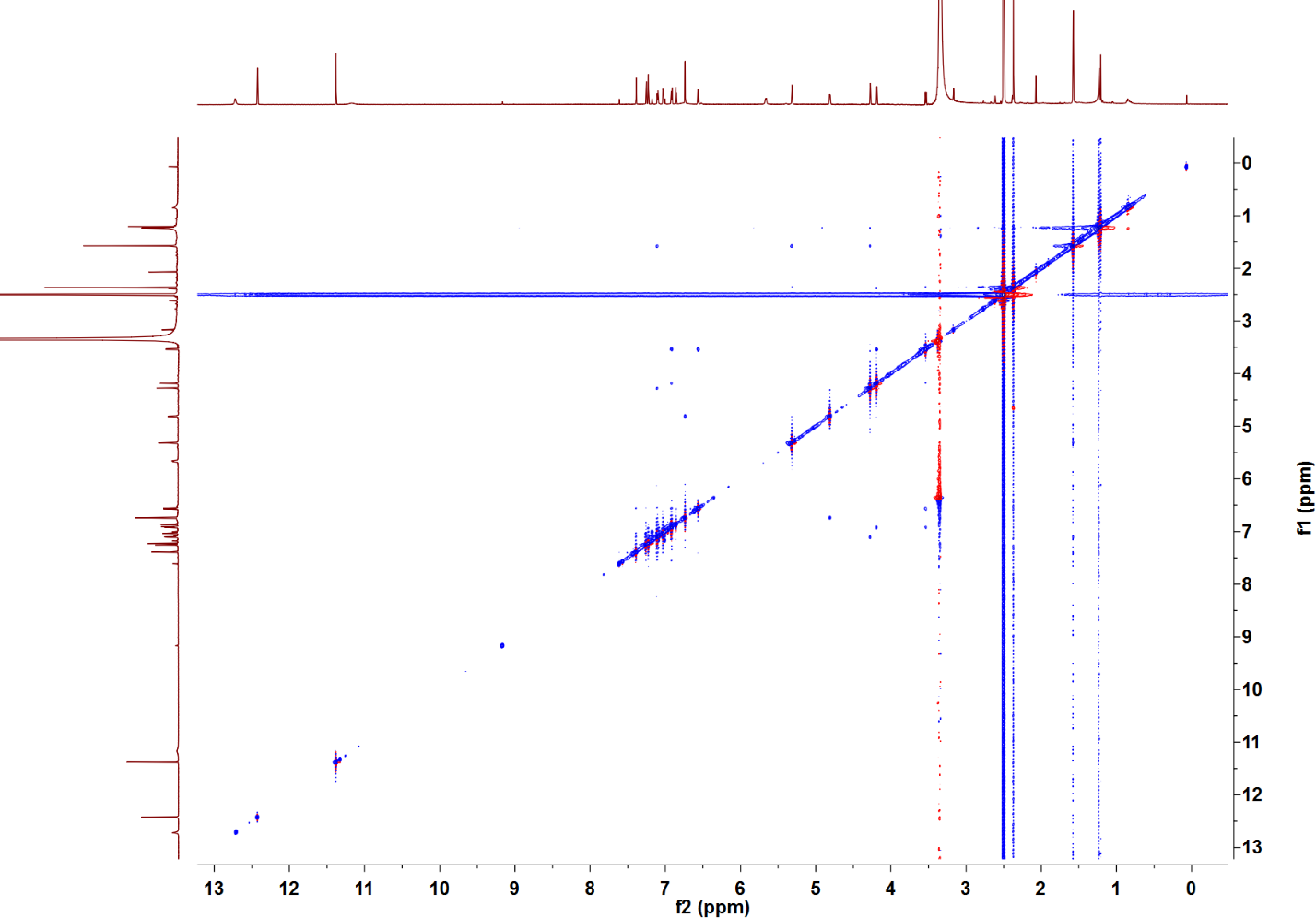

**Supplementary Fig. 39.** NOESY spectrum of bipentaromycin C (**3**) in DMSO-*d*_6_.


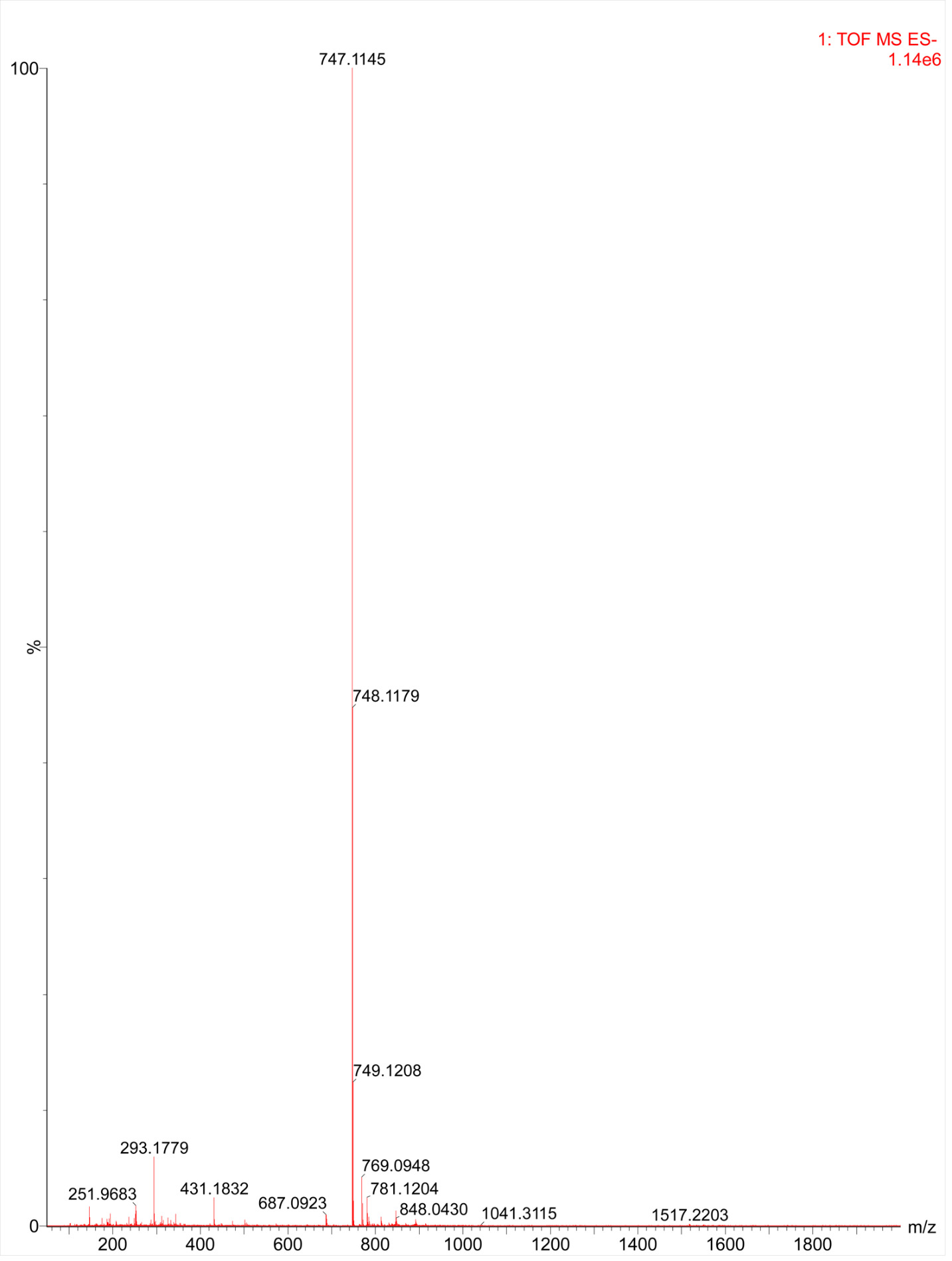


**Supplementary Fig. 40.** HRESIMS spectrum of bipentaromycin D (**4**).


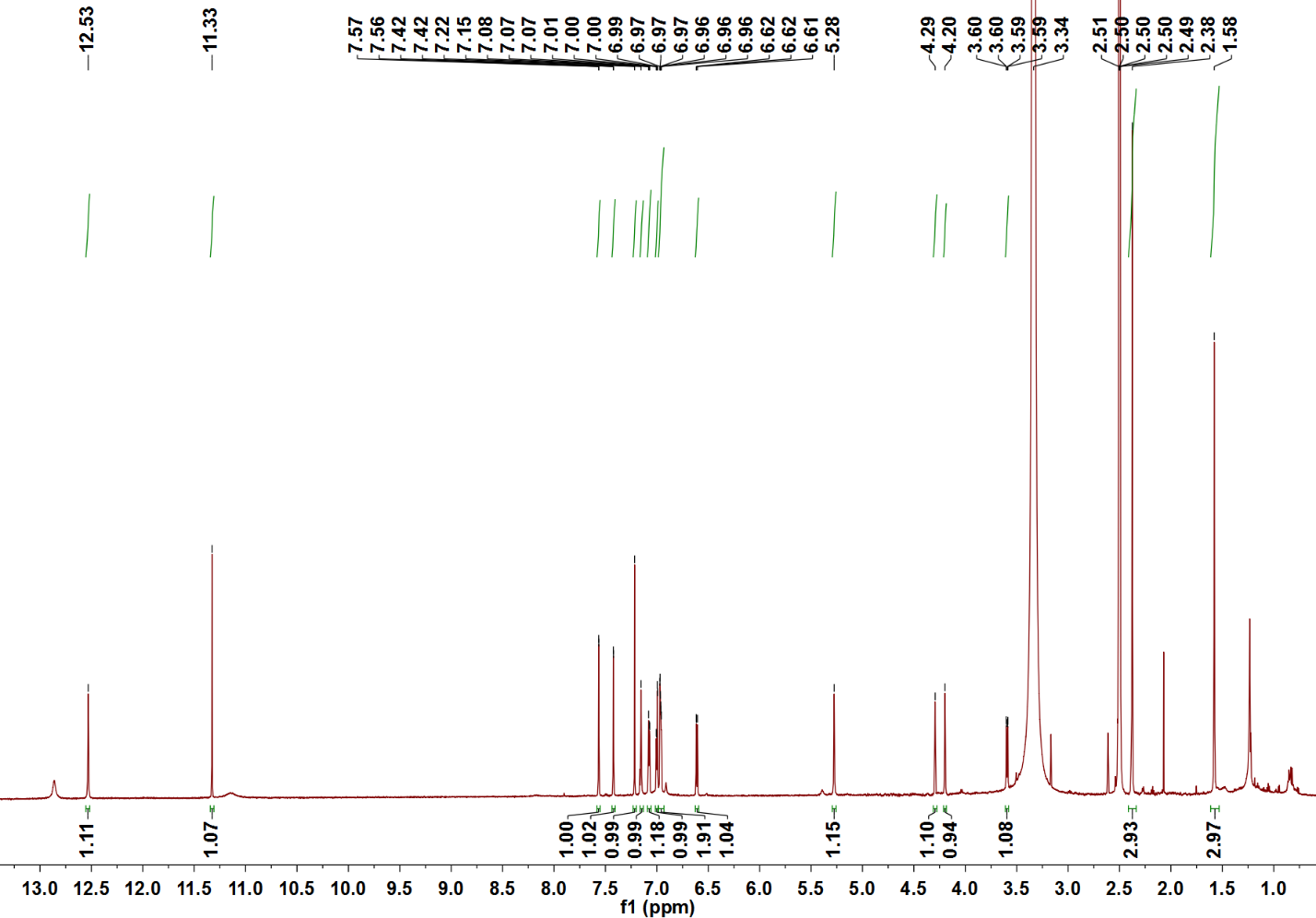


**Supplementary Fig. 41.** ^1^H NMR spectrum of bipentaromycin D (**4**) in DMSO-*d*_6_.


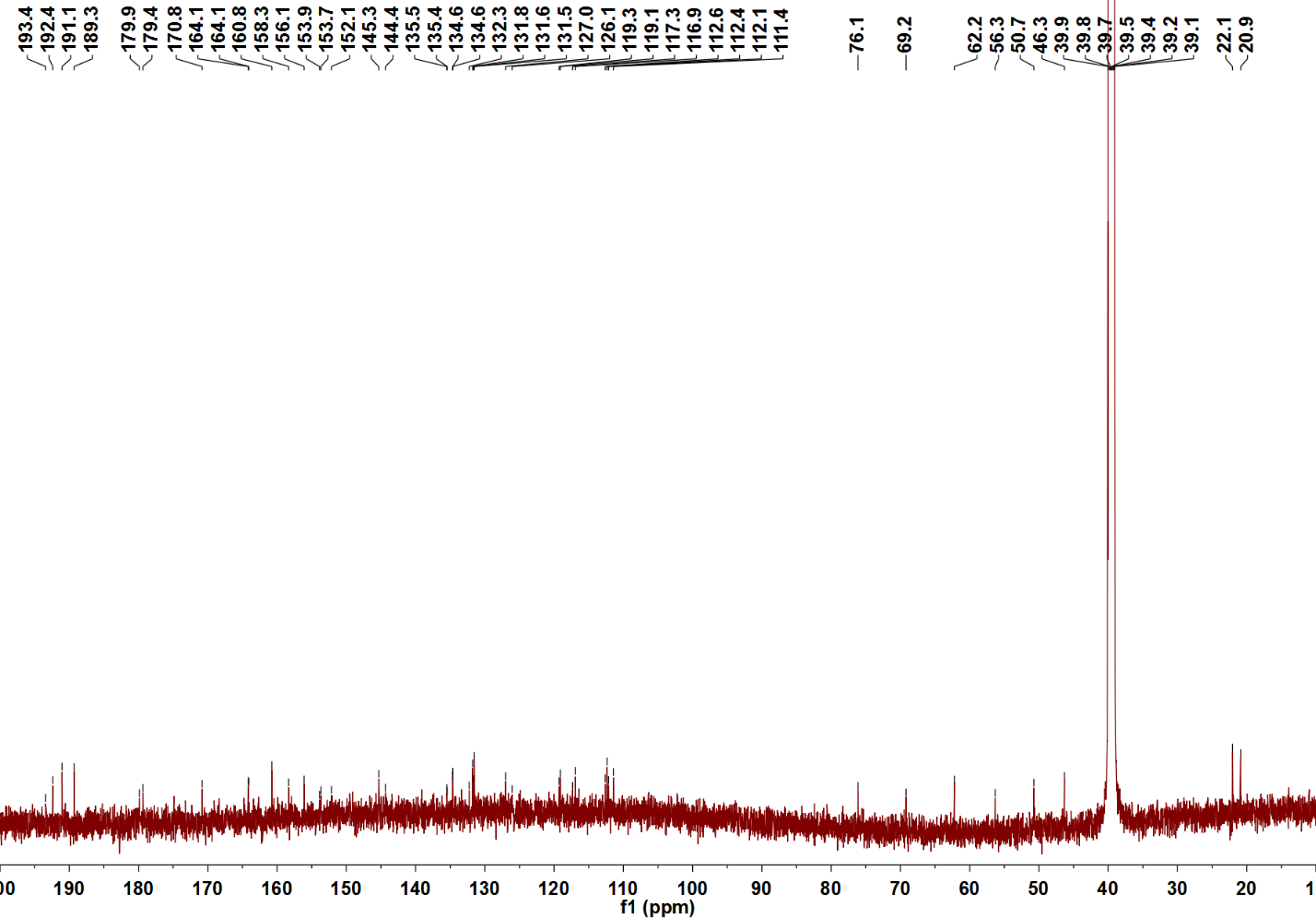


**Supplementary Fig. 42.** ^13^C NMR spectrum of bipentaromycin D (**4**) in DMSO-*d*_6_.


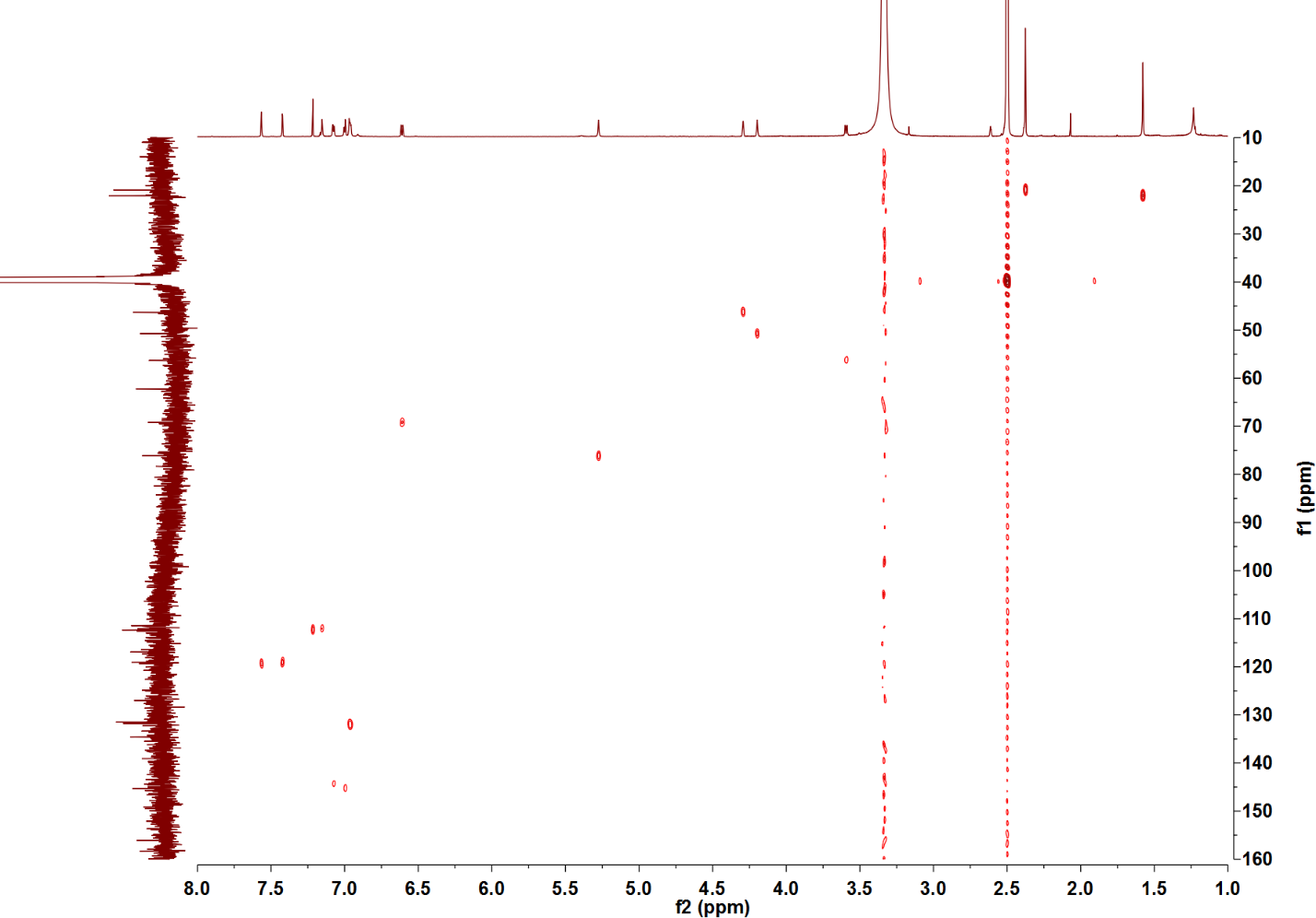


**Supplementary Fig. 43.** HSQC spectrum of bipentaromycin D (**4**) in DMSO-*d*_6_.


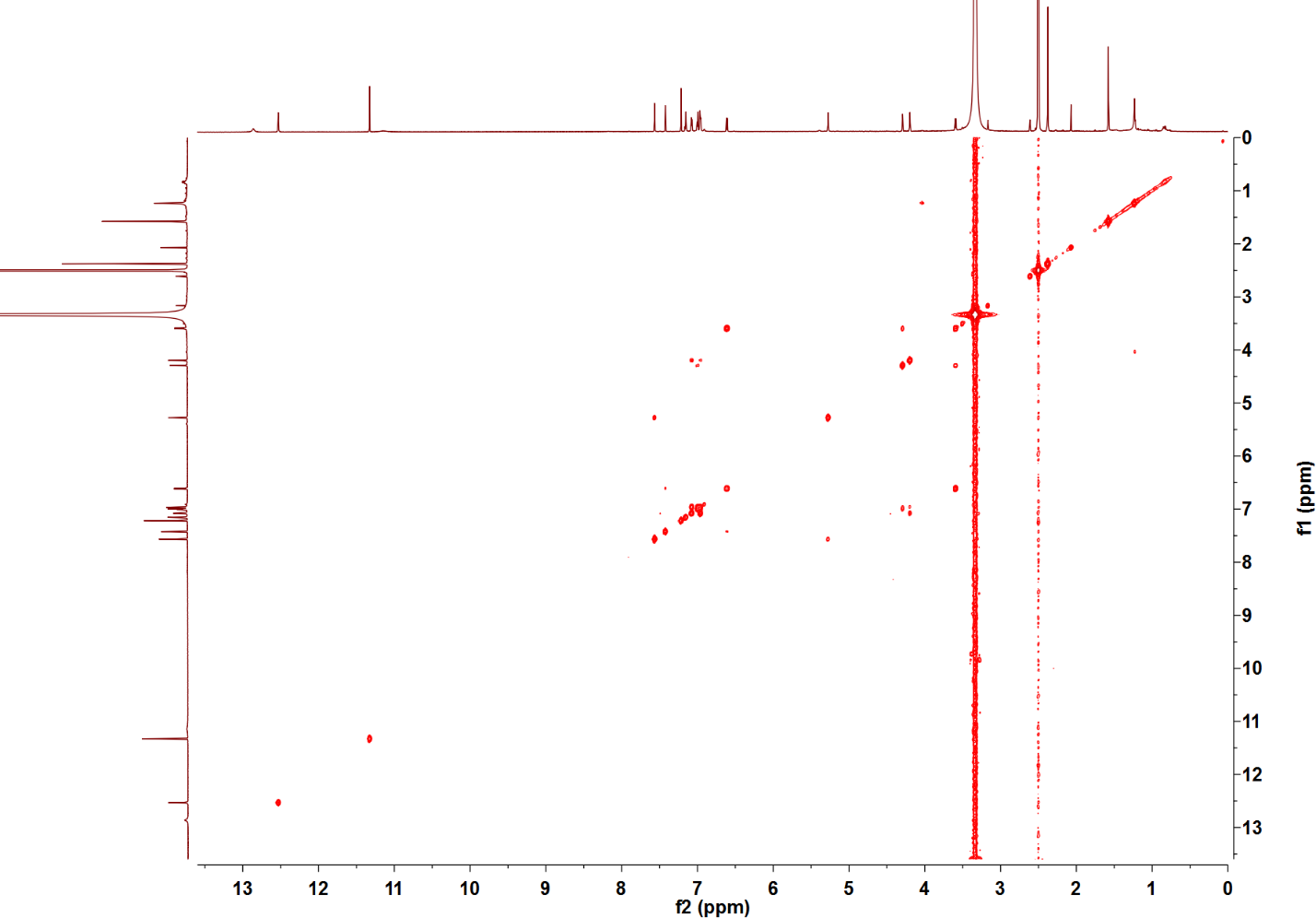

**Supplementary Fig. 44.** COSY spectrum of bipentaromycin D (**4**) in DMSO-*d*_6_.


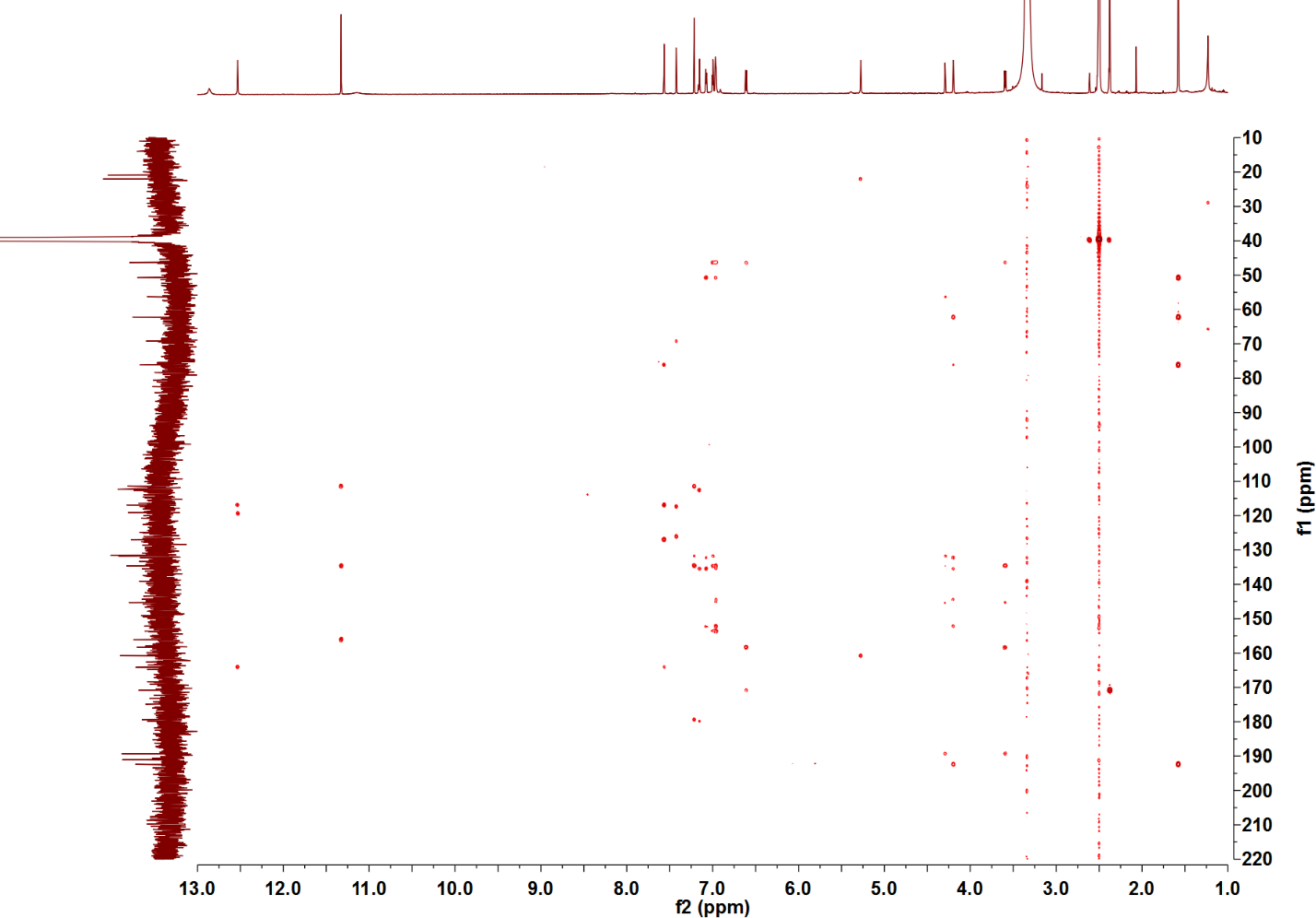

**Supplementary Fig. 45.** HMBC spectrum of bipentaromycin D (**4**) in DMSO-*d*_6_.


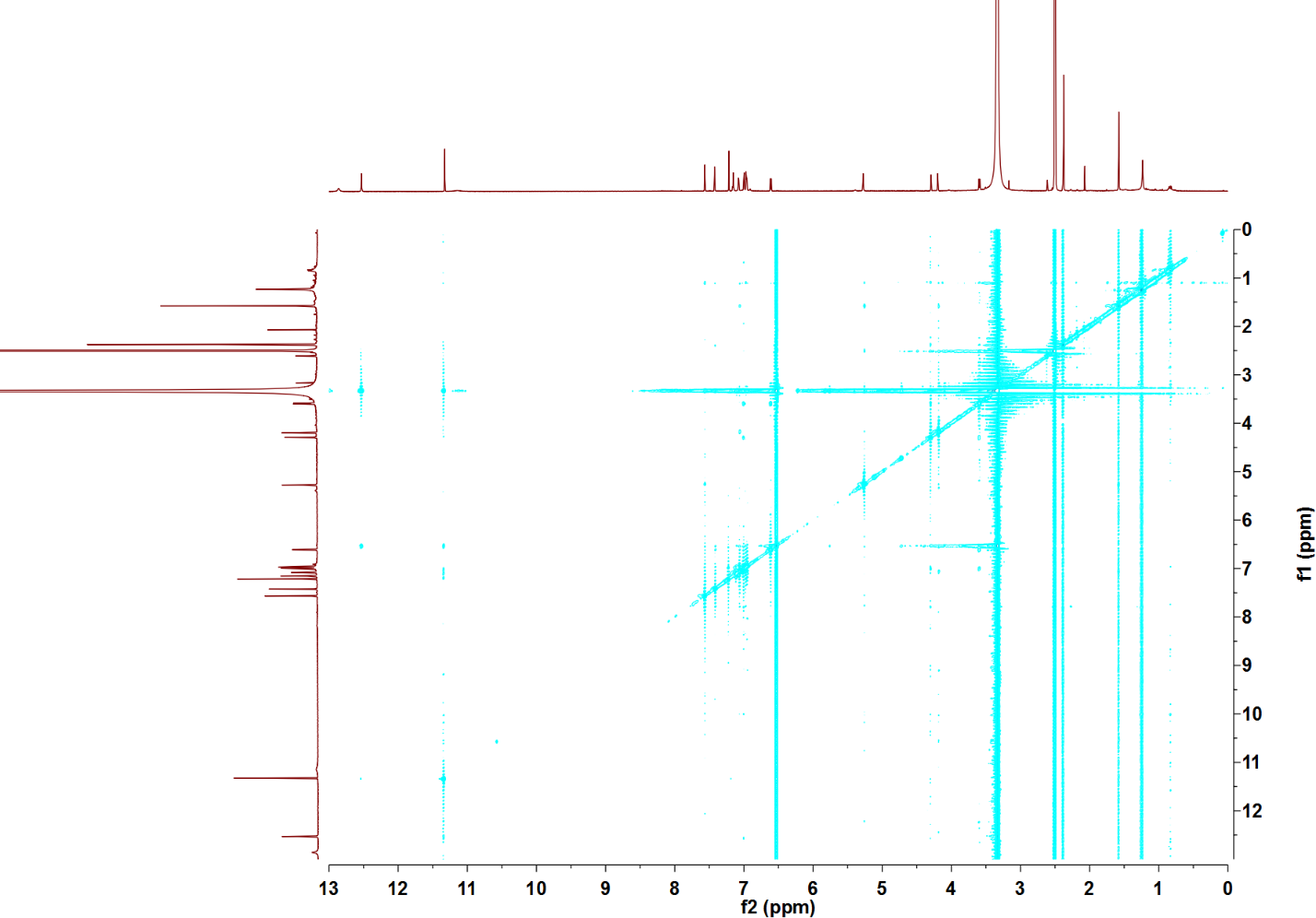

**Supplementary Fig. 46.** NOESY spectrum of bipentaromycin D (**4**) in DMSO-*d*_6_.


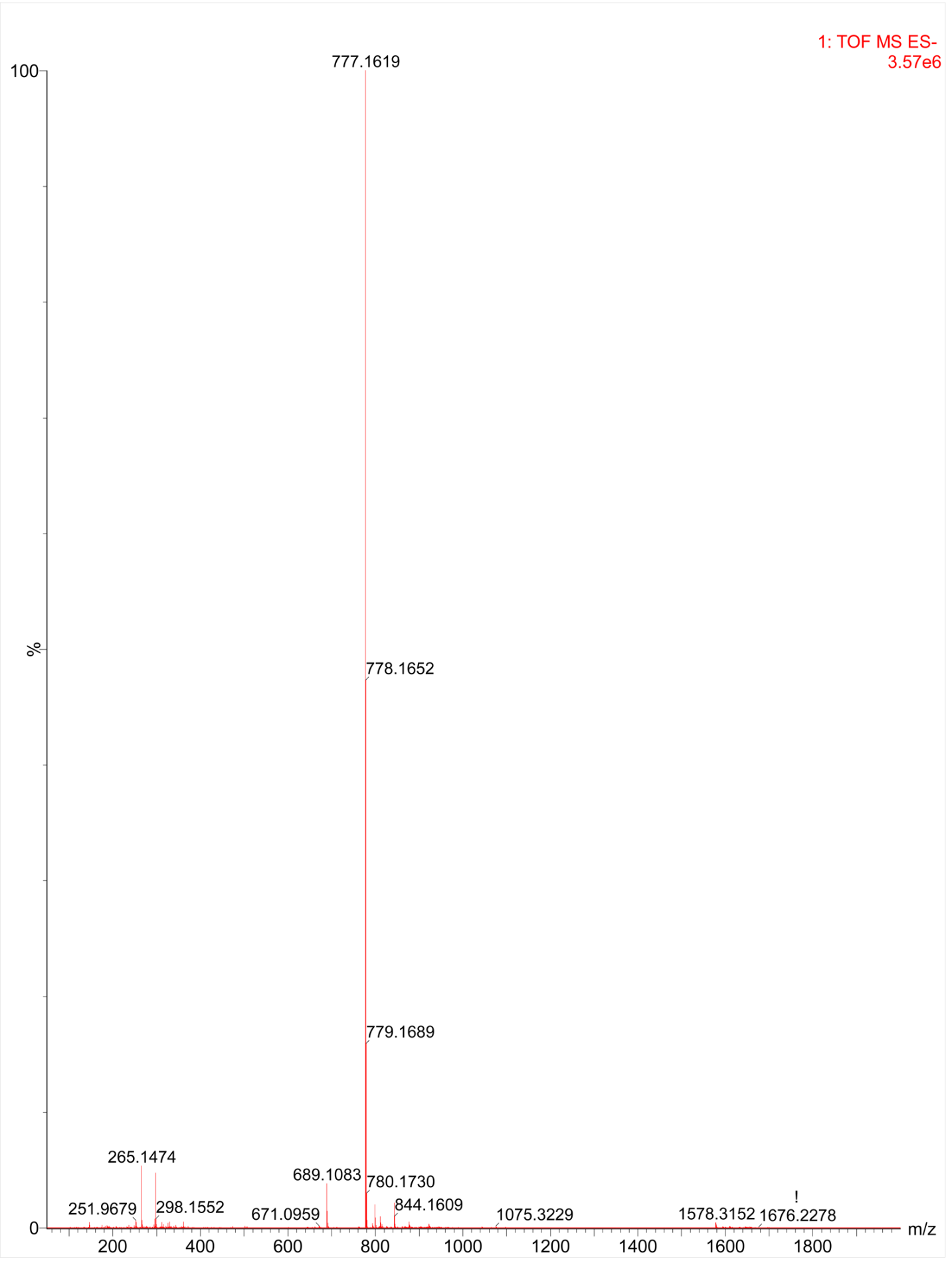


**Supplementary Fig. 47.** HRESIMS spectrum of bipentaromycin E (**5**).


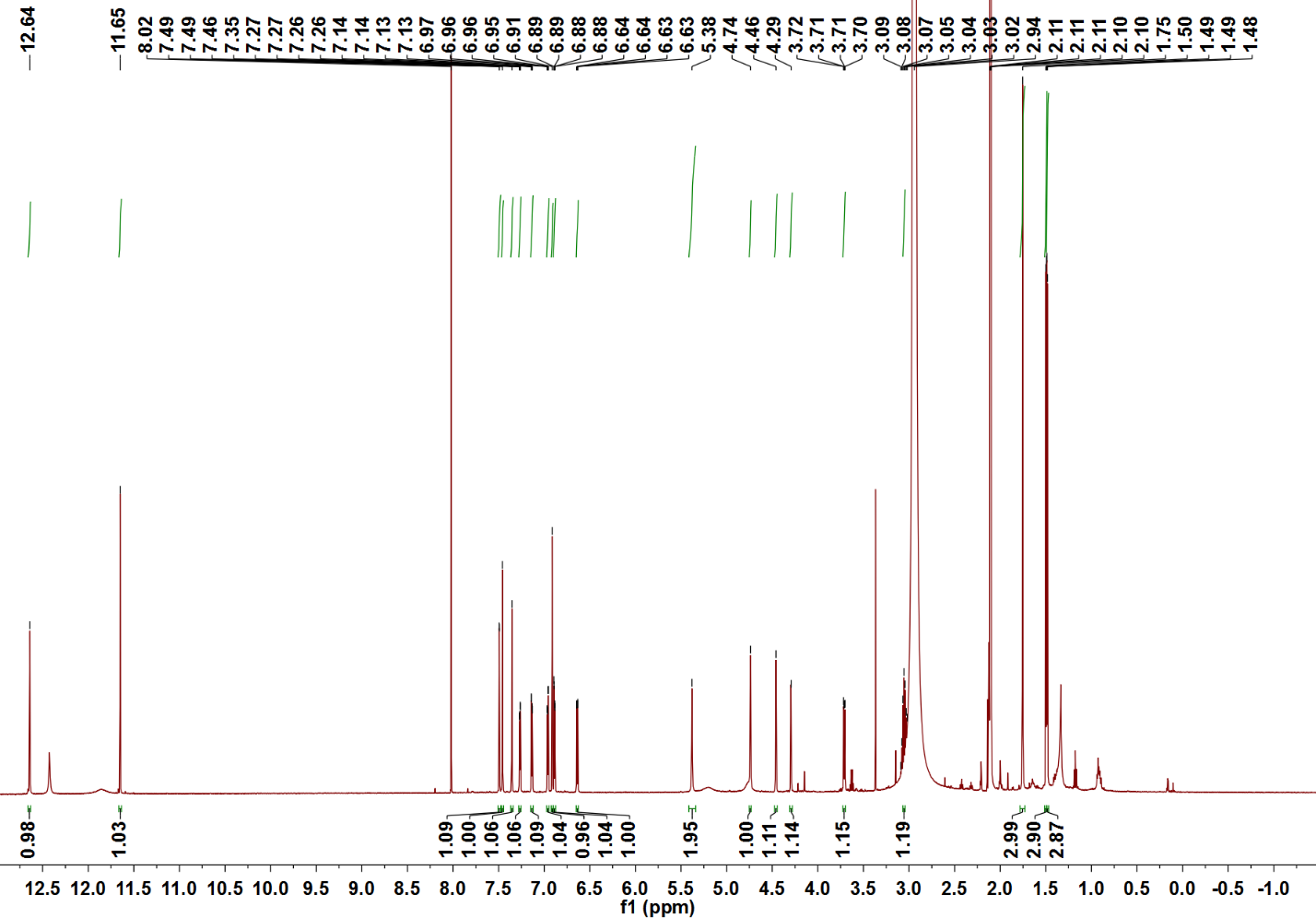


**Supplementary Fig. 48.** ^1^H NMR spectrum of bipentaromycin E (**5**) in (CD_3_)_2_CO/CDCl_3_.


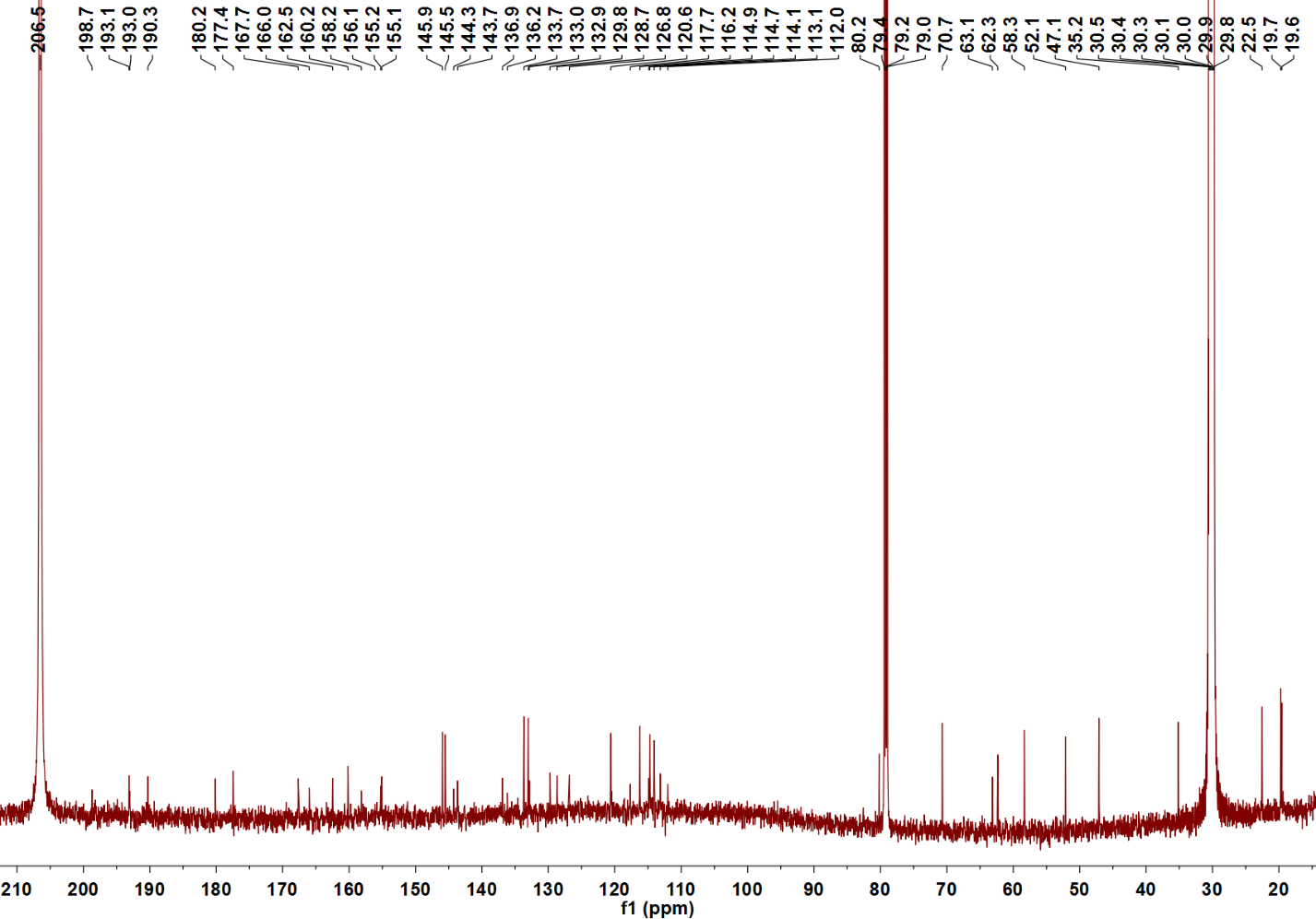


**Supplementary Fig. 49.** ^13^C NMR spectrum of bipentaromycin E (**5**) in (CD_3_)_2_CO/CDCl_3_.


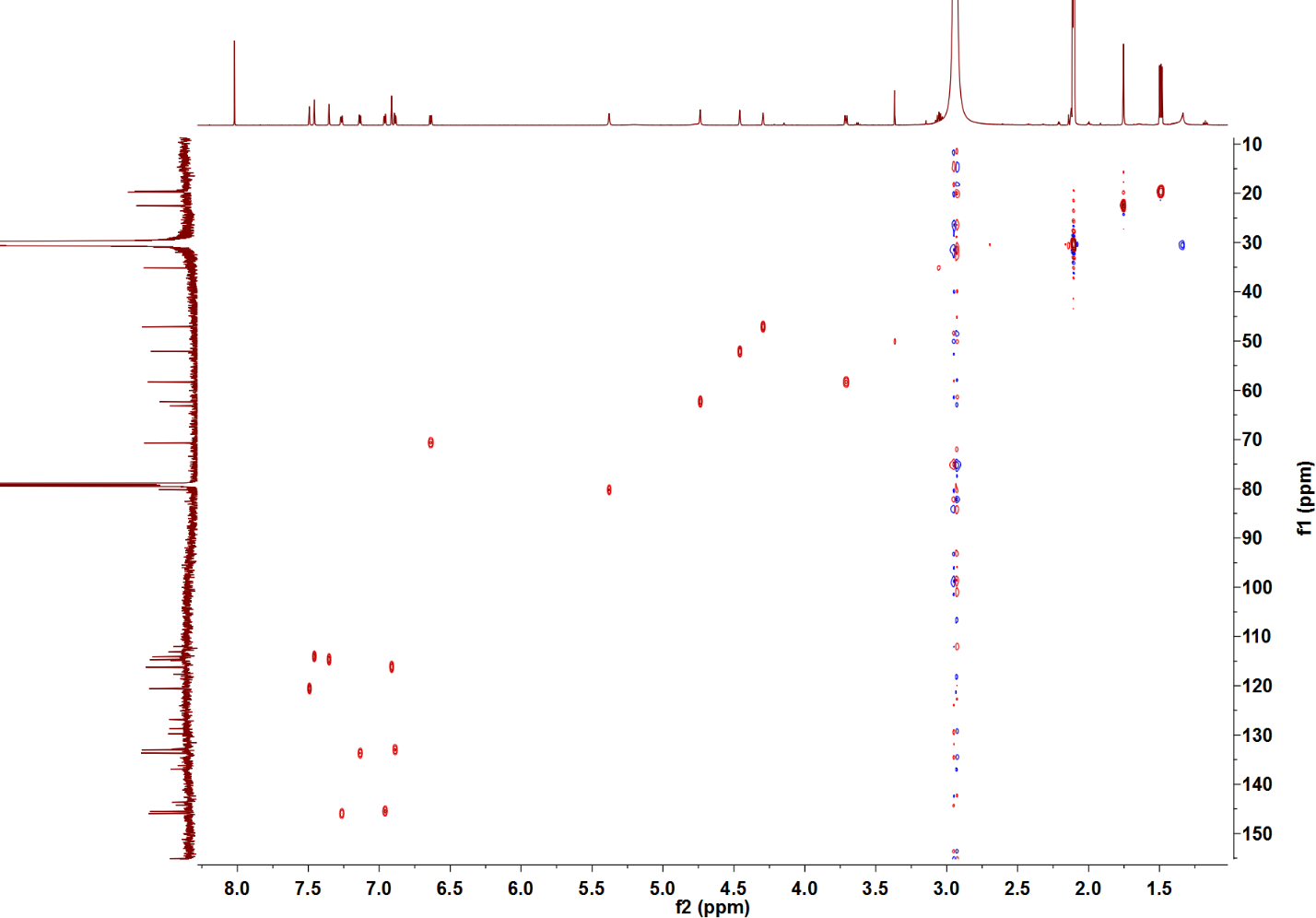


**Supplementary Fig. 50.** HSQC spectrum of bipentaromycin E (**5**) in (CD_3_)_2_CO/CDCl_3_.


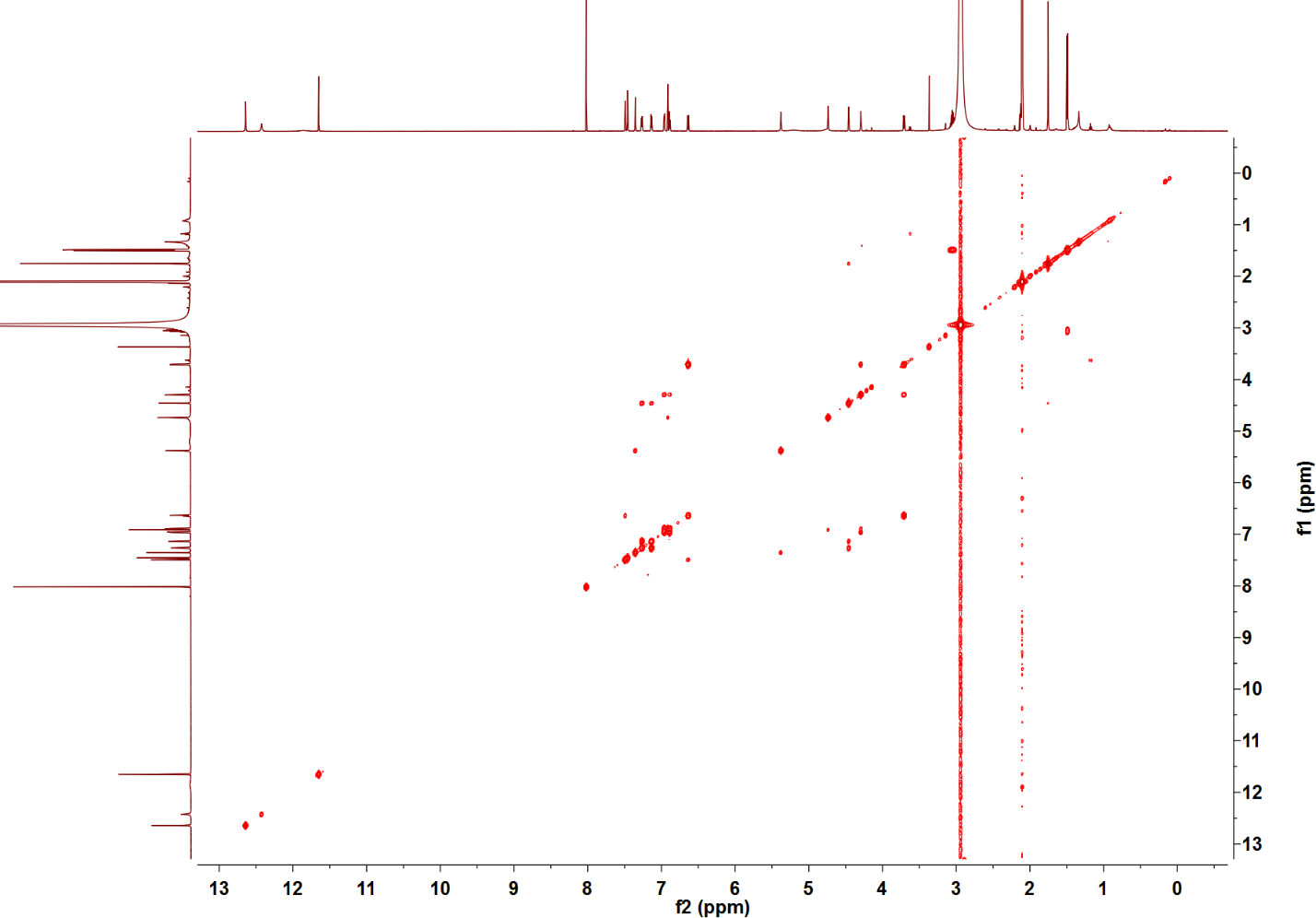

**Supplementary Fig. 51.** COSY spectrum of bipentaromycin E (**5**) in (CD_3_)_2_CO/CDCl_3_.


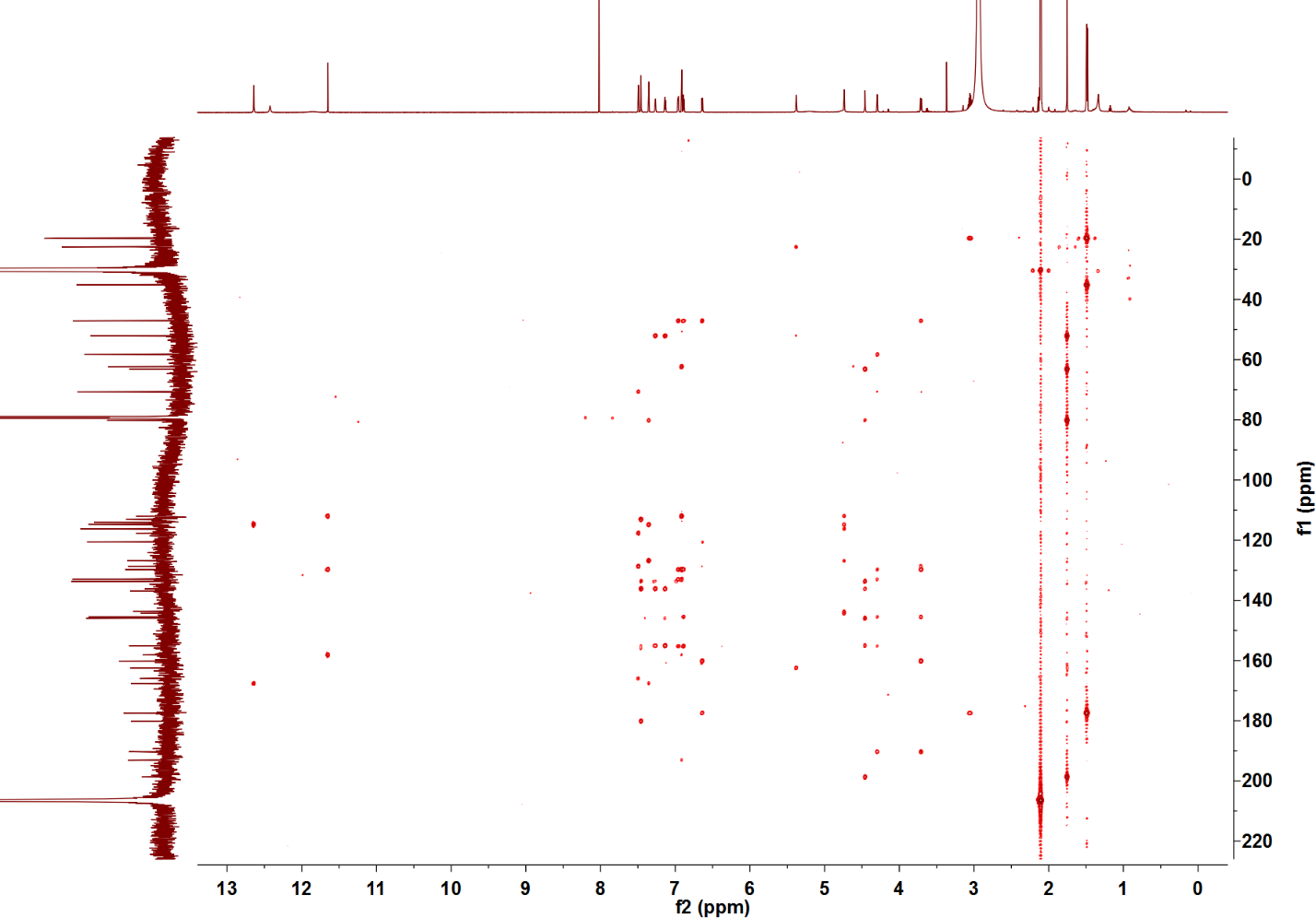

**Supplementary Fig. 52.** HMBC spectrum of bipentaromycin E (**5**) in (CD_3_)_2_CO/CDCl_3_.


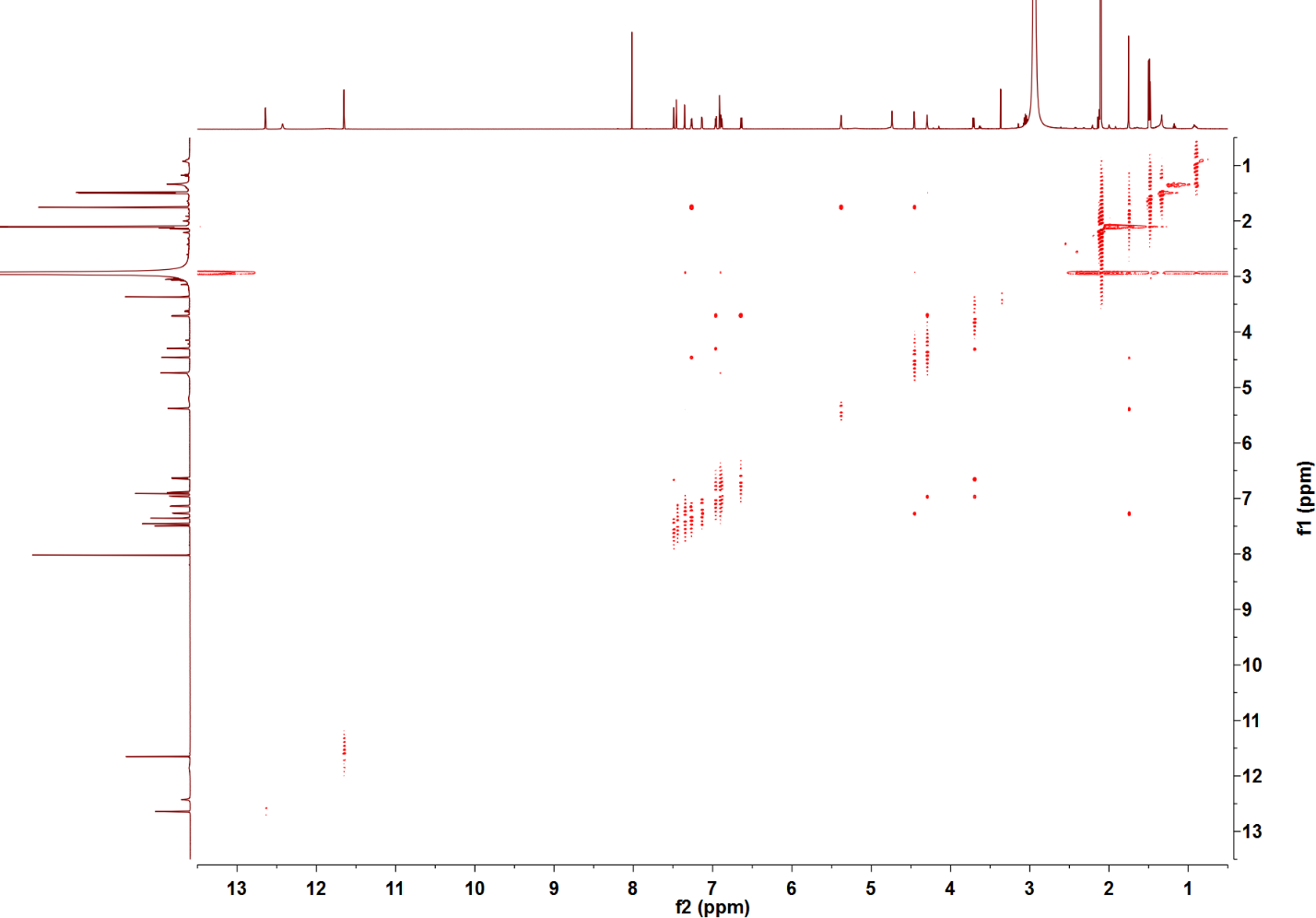

**Supplementary Fig. 53.** NOESY spectrum of bipentaromycin E (**5**) in (CD_3_)_2_CO/CDCl_3_.


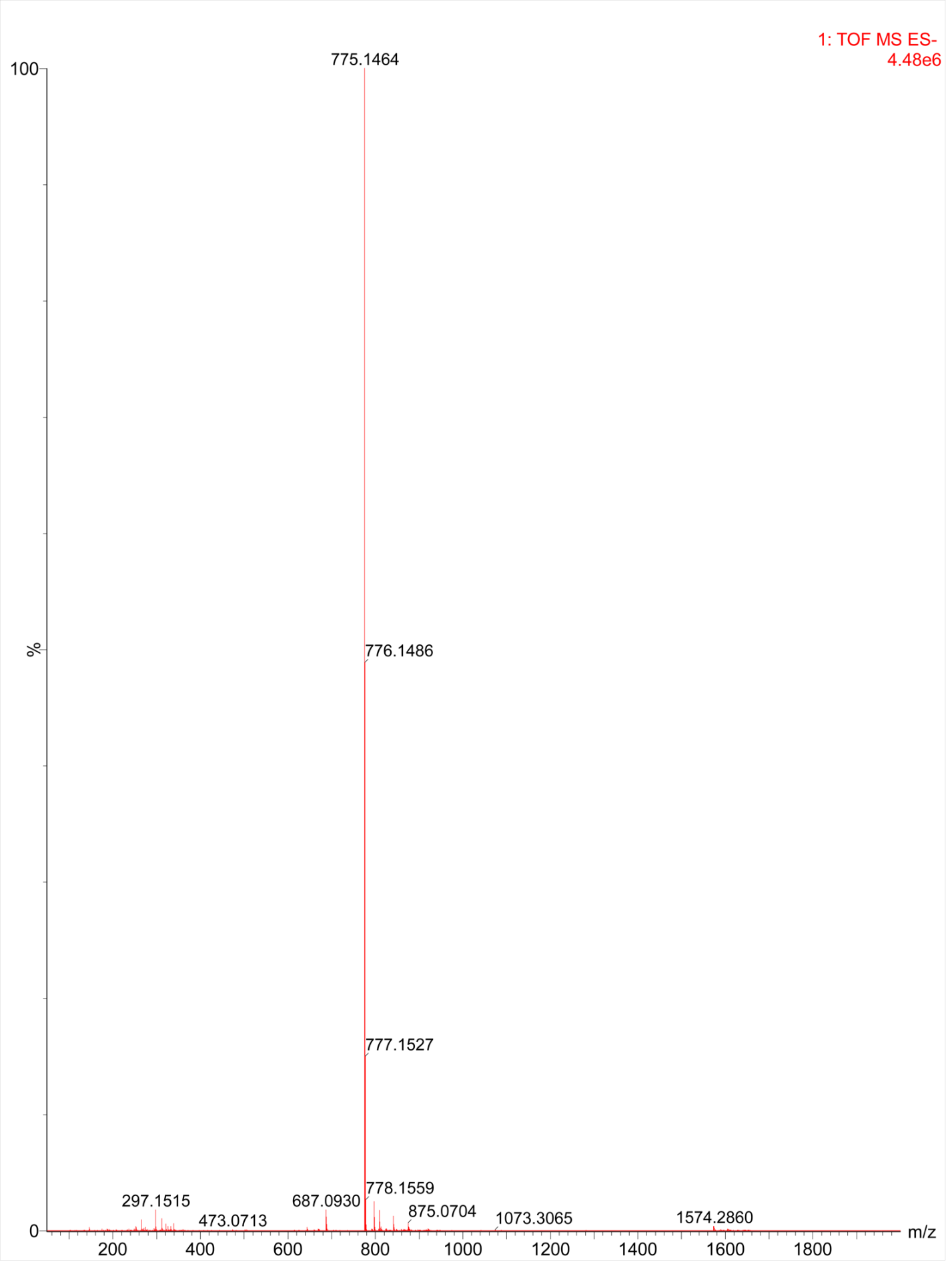


**Supplementary Fig. 54.** HRESIMS spectrum of bipentaromycin F (**6**).


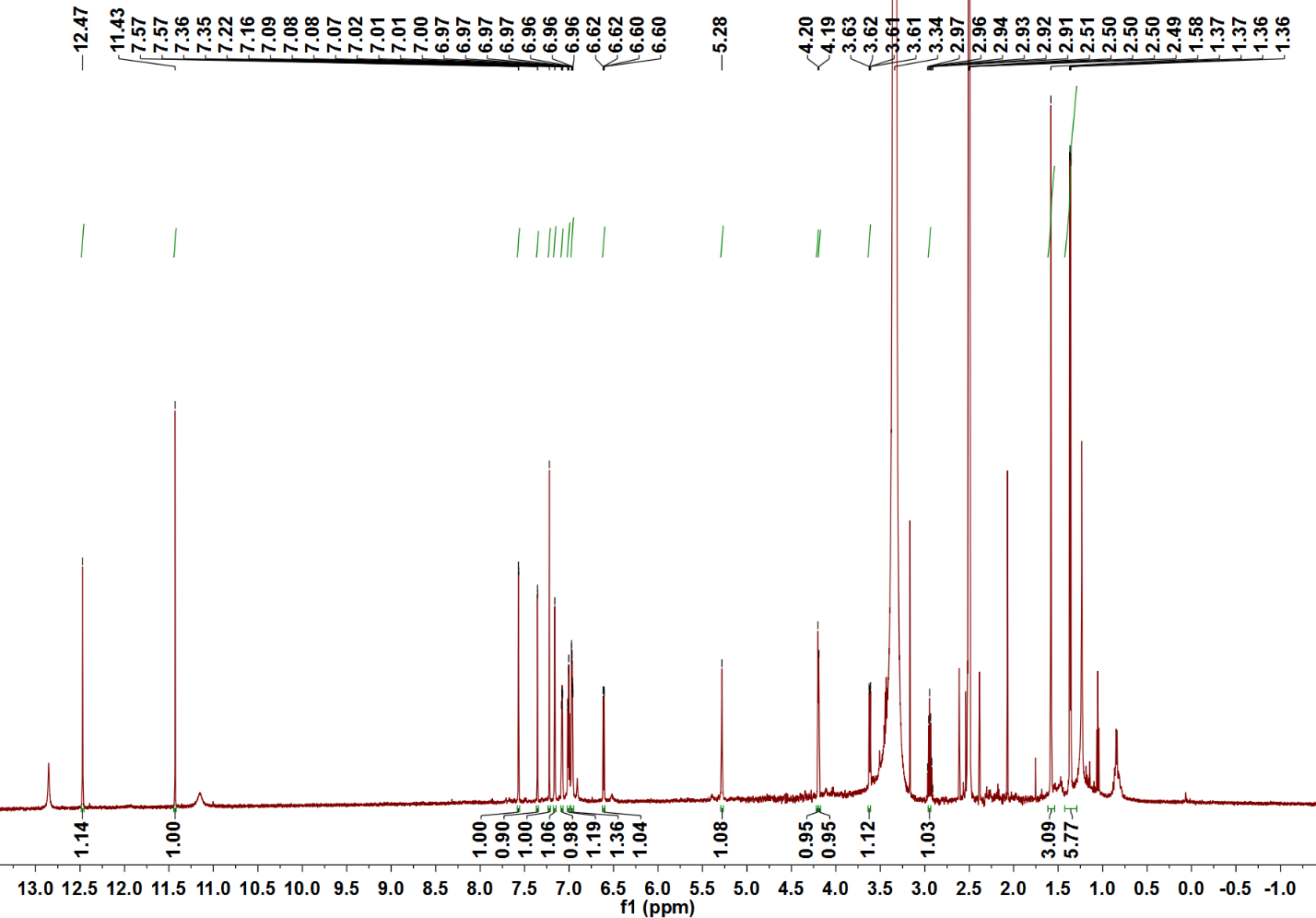


**Supplementary Fig. 55.** ^1^H NMR spectrum of bipentaromycin F (**6**) in DMSO-*d*_6_.


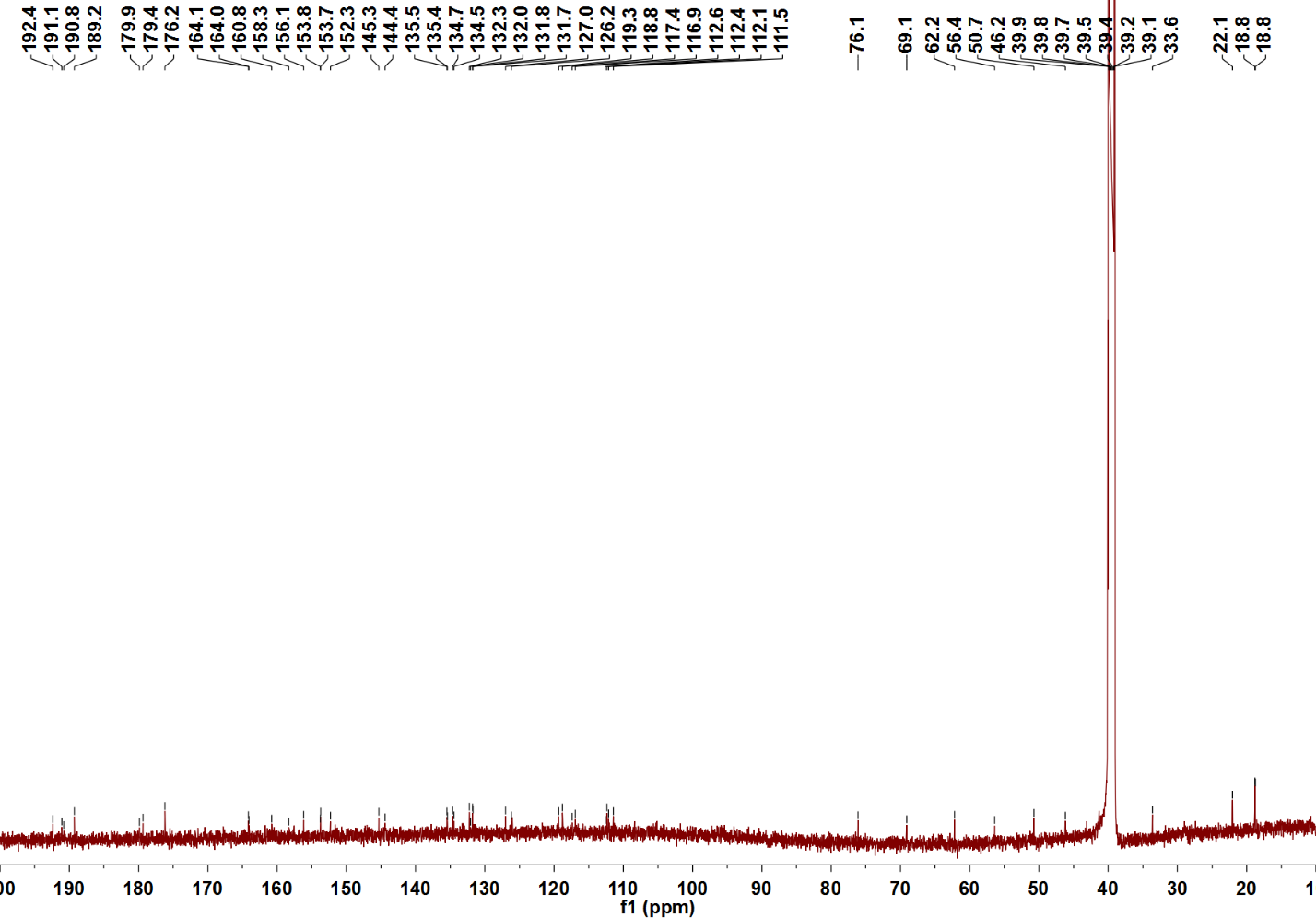


**Supplementary Fig. 56.** ^13^C NMR spectrum of bipentaromycin F (**6**) in DMSO-*d*_6_.


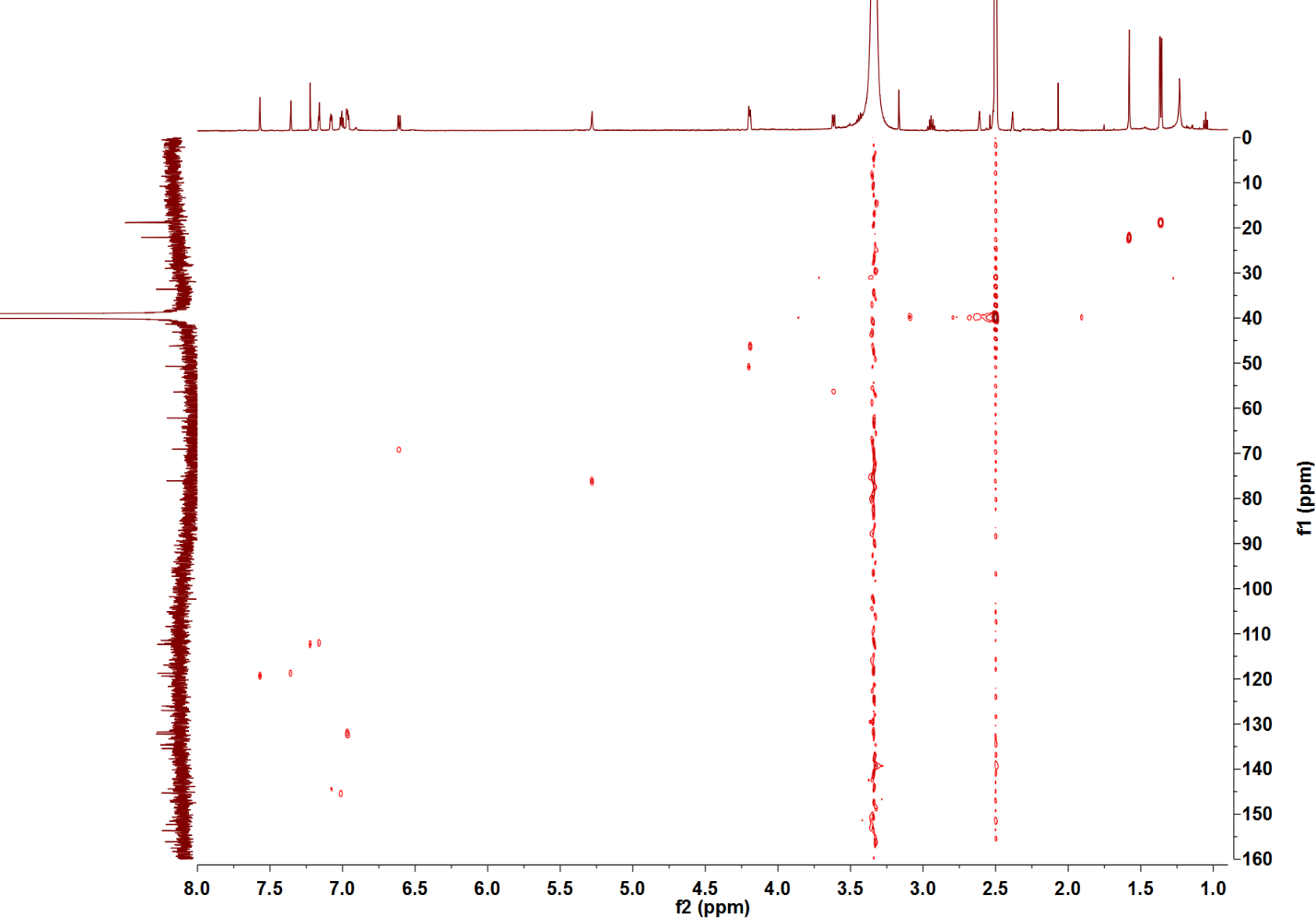


**Supplementary Fig. 57.** HSQC spectrum of bipentaromycin F (**6**) in DMSO-*d*_6_.


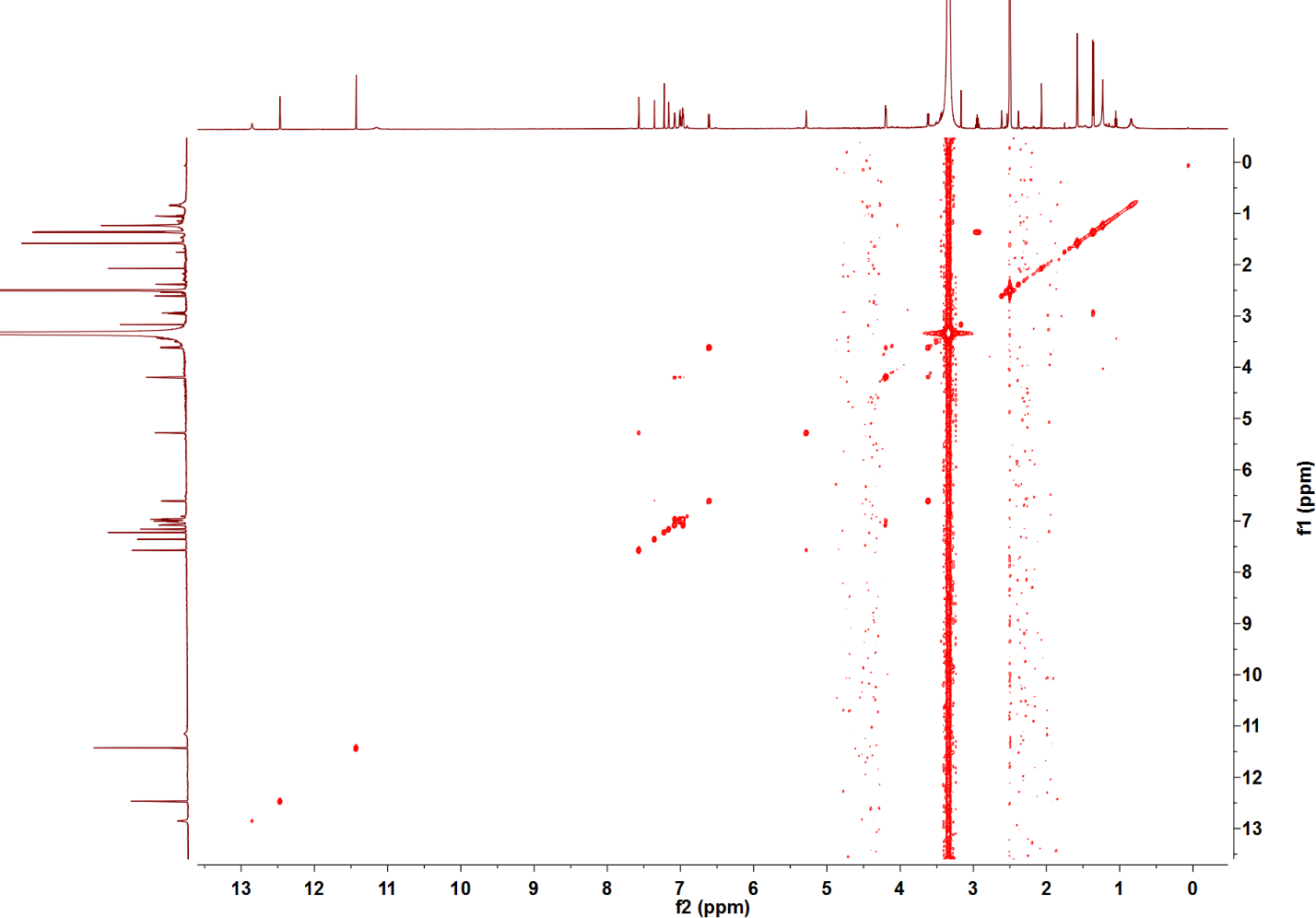

**Supplementary Fig. 58.** COSY spectrum of bipentaromycin F (**6**) in DMSO-*d*_6_.


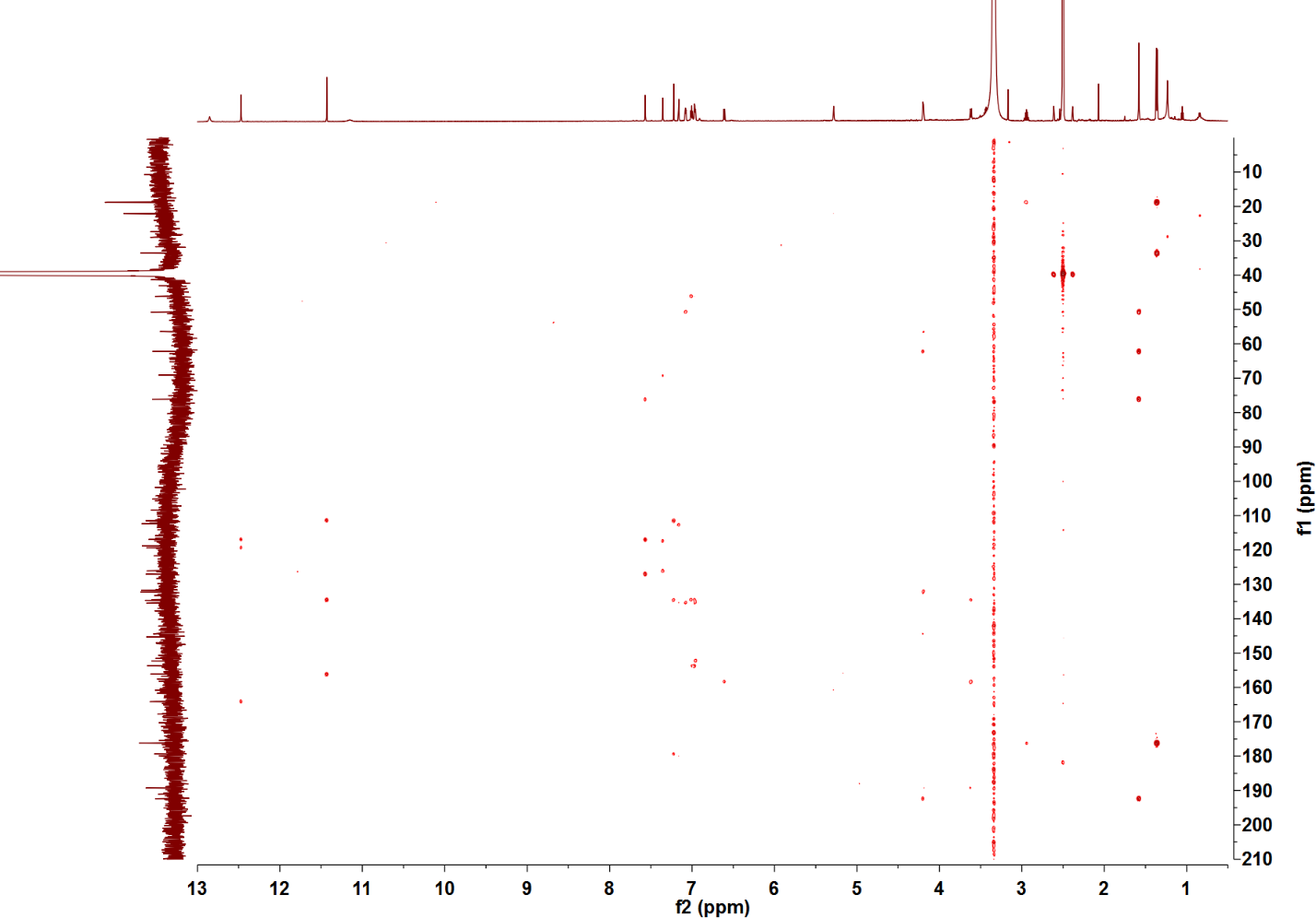

**Supplementary Fig. 59.** HMBC spectrum of bipentaromycin F (**6**) in DMSO-*d*_6_.


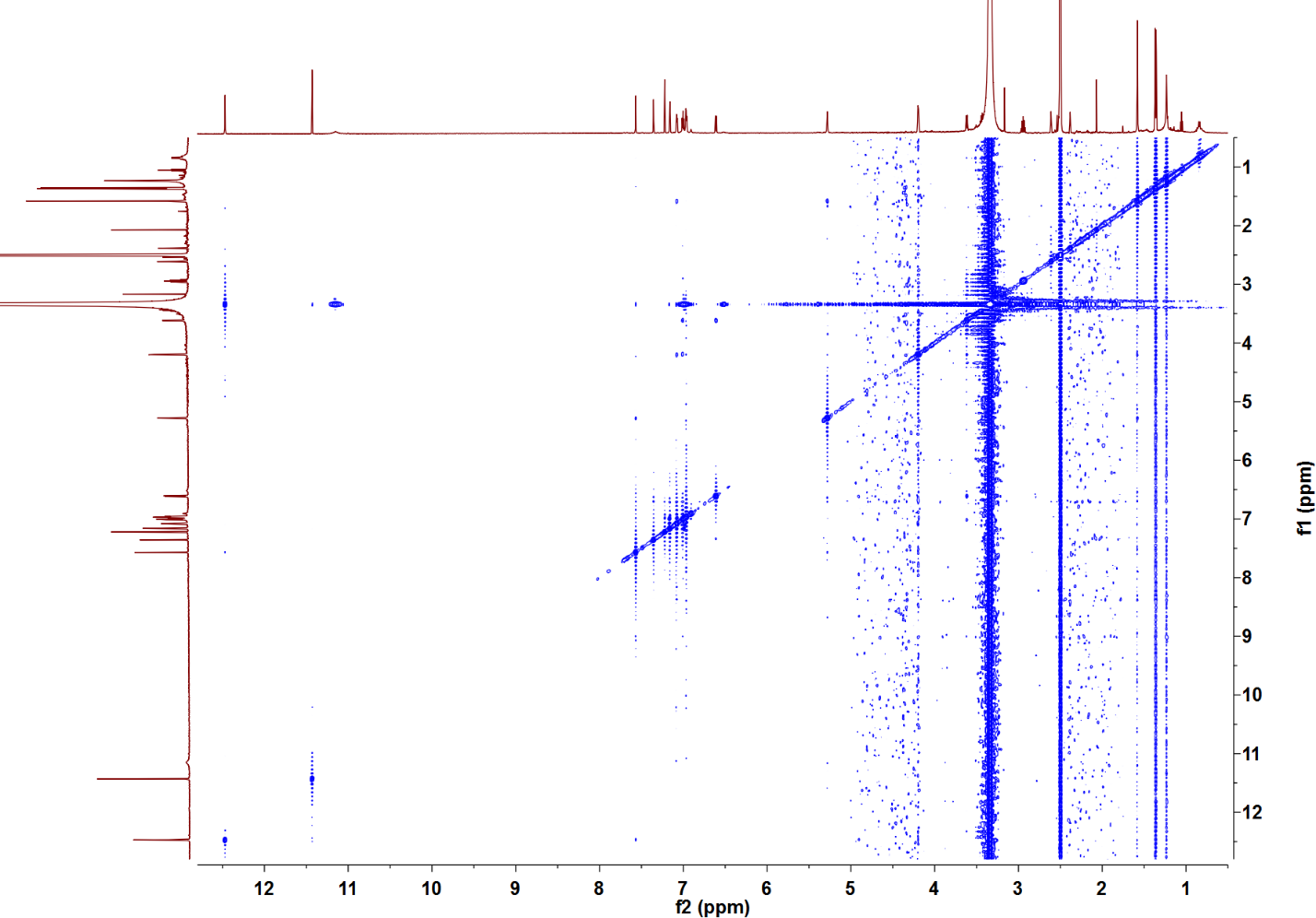

**Supplementary Fig. 60.** NOESY spectrum of bipentaromycin F (**6**) in DMSO-*d*_6_.


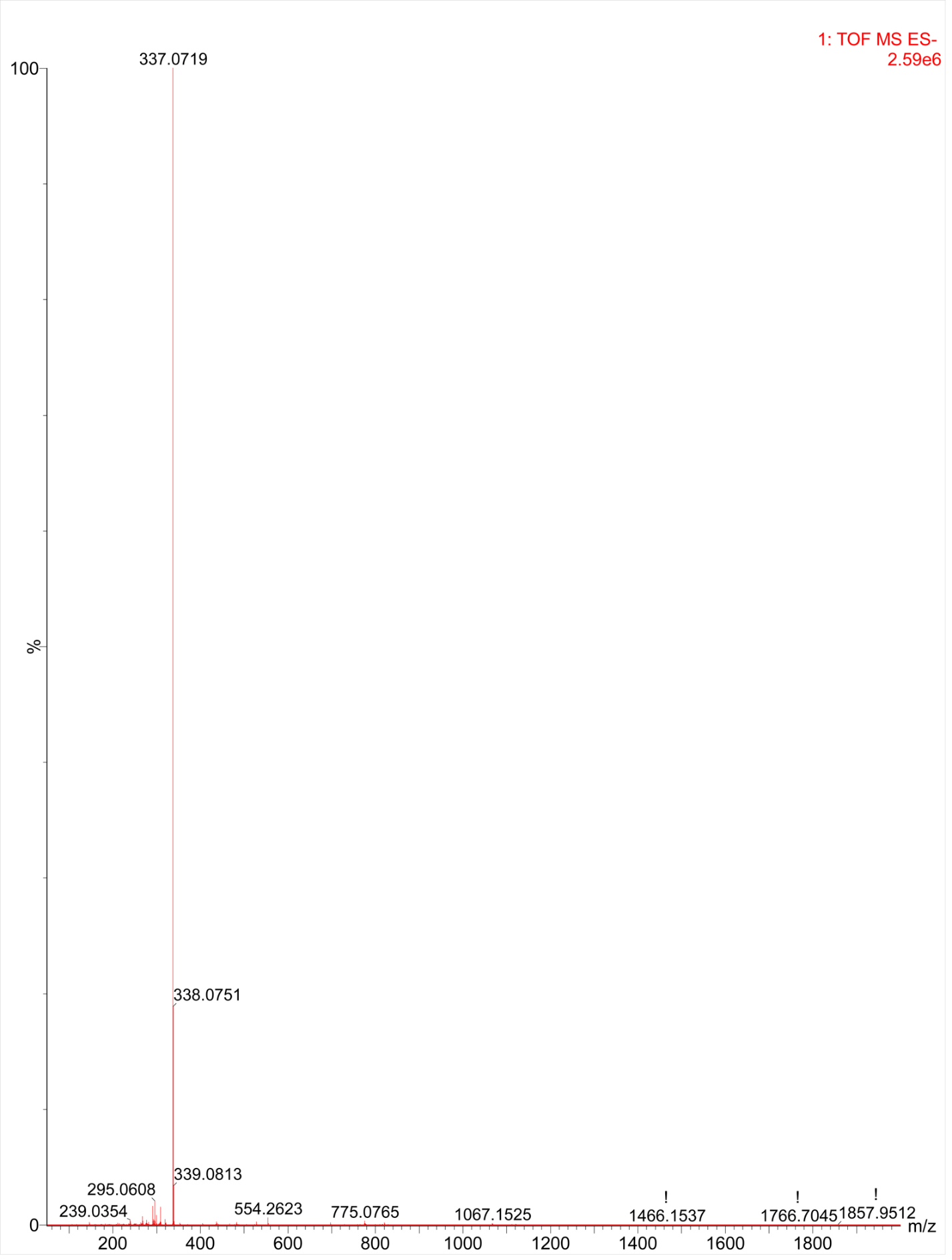


**Supplementary Fig. 61.** HRESIMS spectrum of 6-hydroxyfujianmycin A_1_ (**7**).


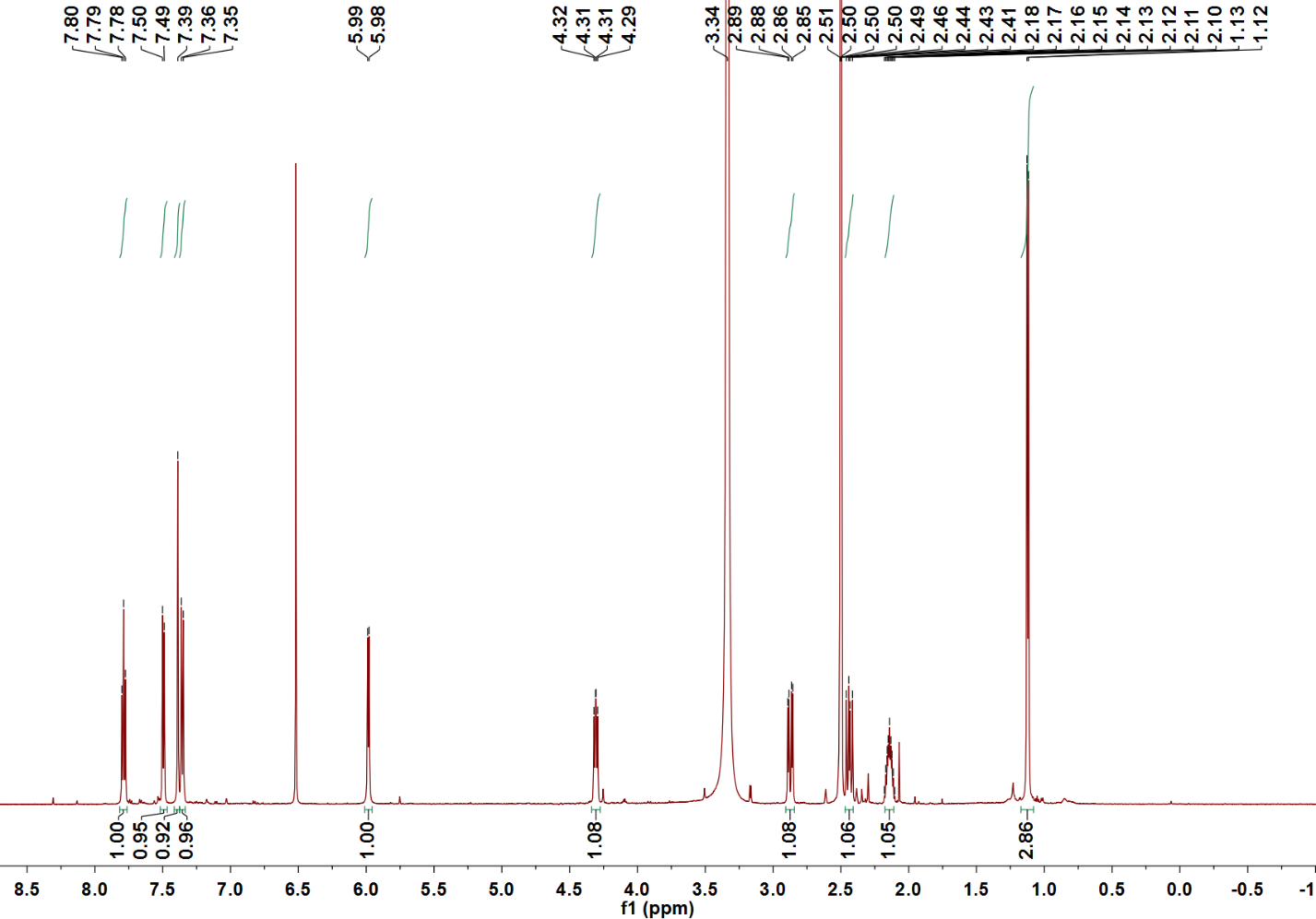


**Supplementary Fig. 62.** ^1^H NMR spectrum of 6-hydroxyfujianmycin A_1_ (**7**) in DMSO-*d*_6_.


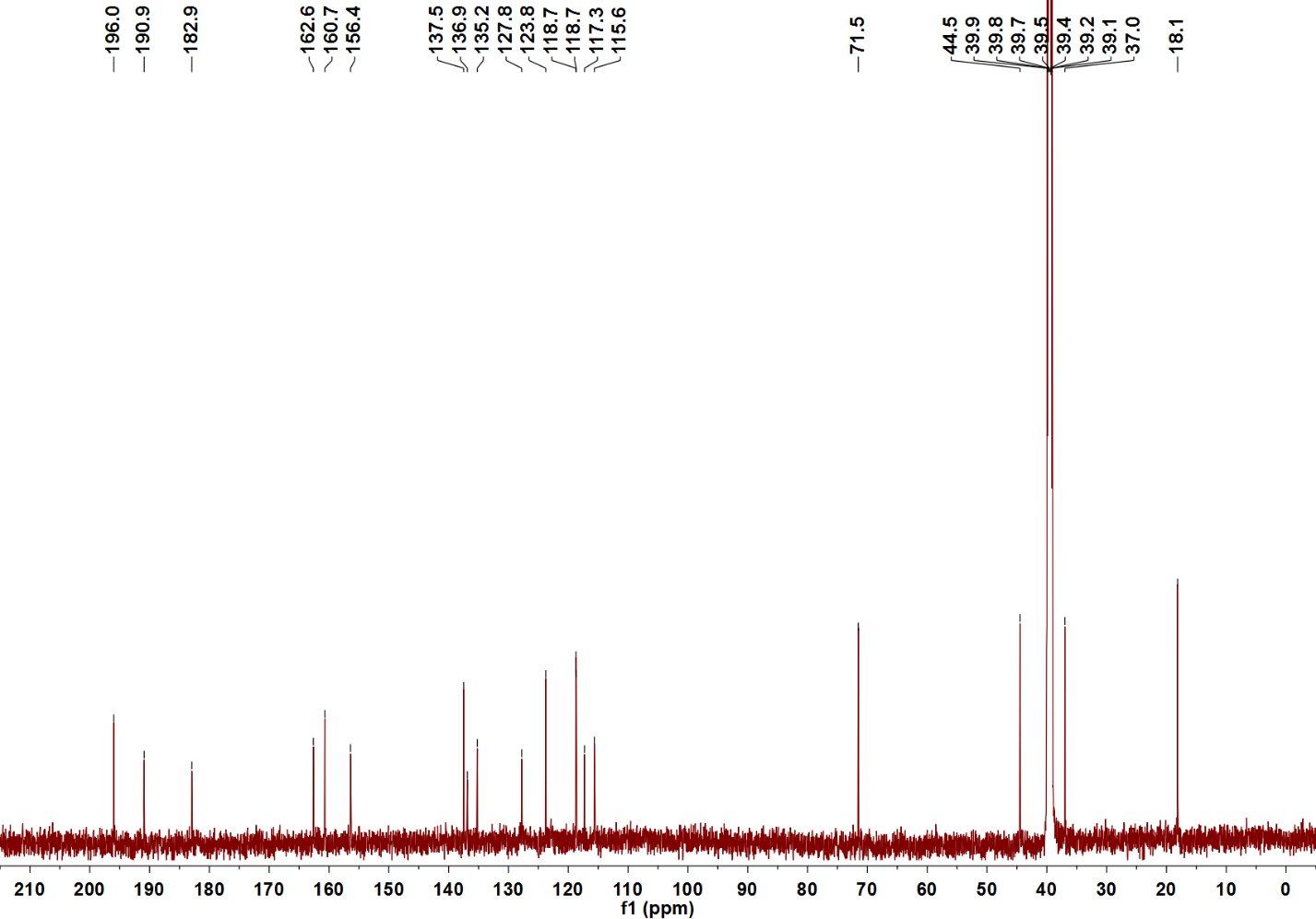


**Supplementary Fig. 63.** ^13^C NMR spectrum of 6-hydroxyfujianmycin A_1_ (**7**) in DMSO-*d*_6_.


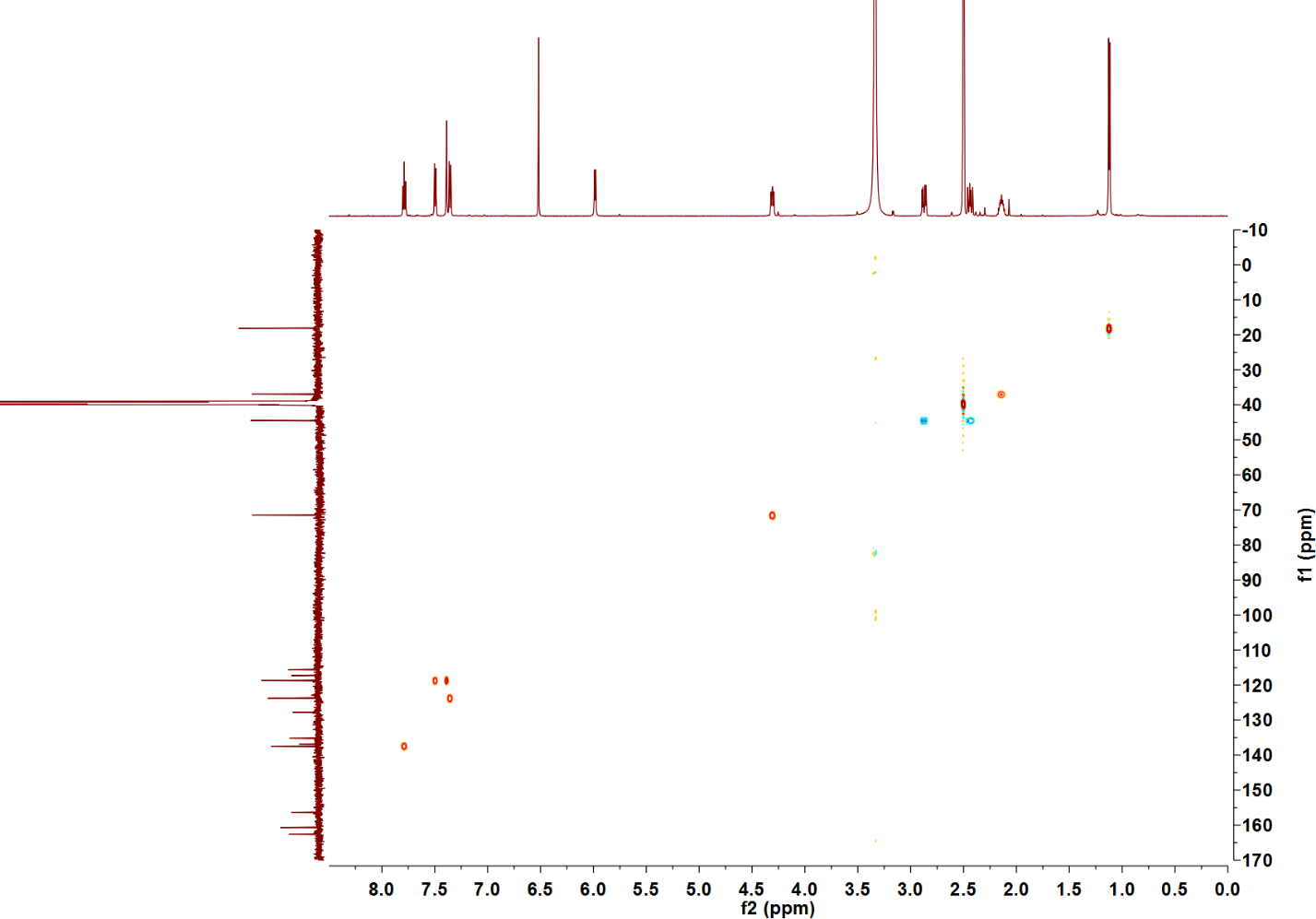


**Supplementary Fig. 64.** HSQC spectrum of 6-hydroxyfujianmycin A_1_ (**7**) in DMSO-*d*_6_.


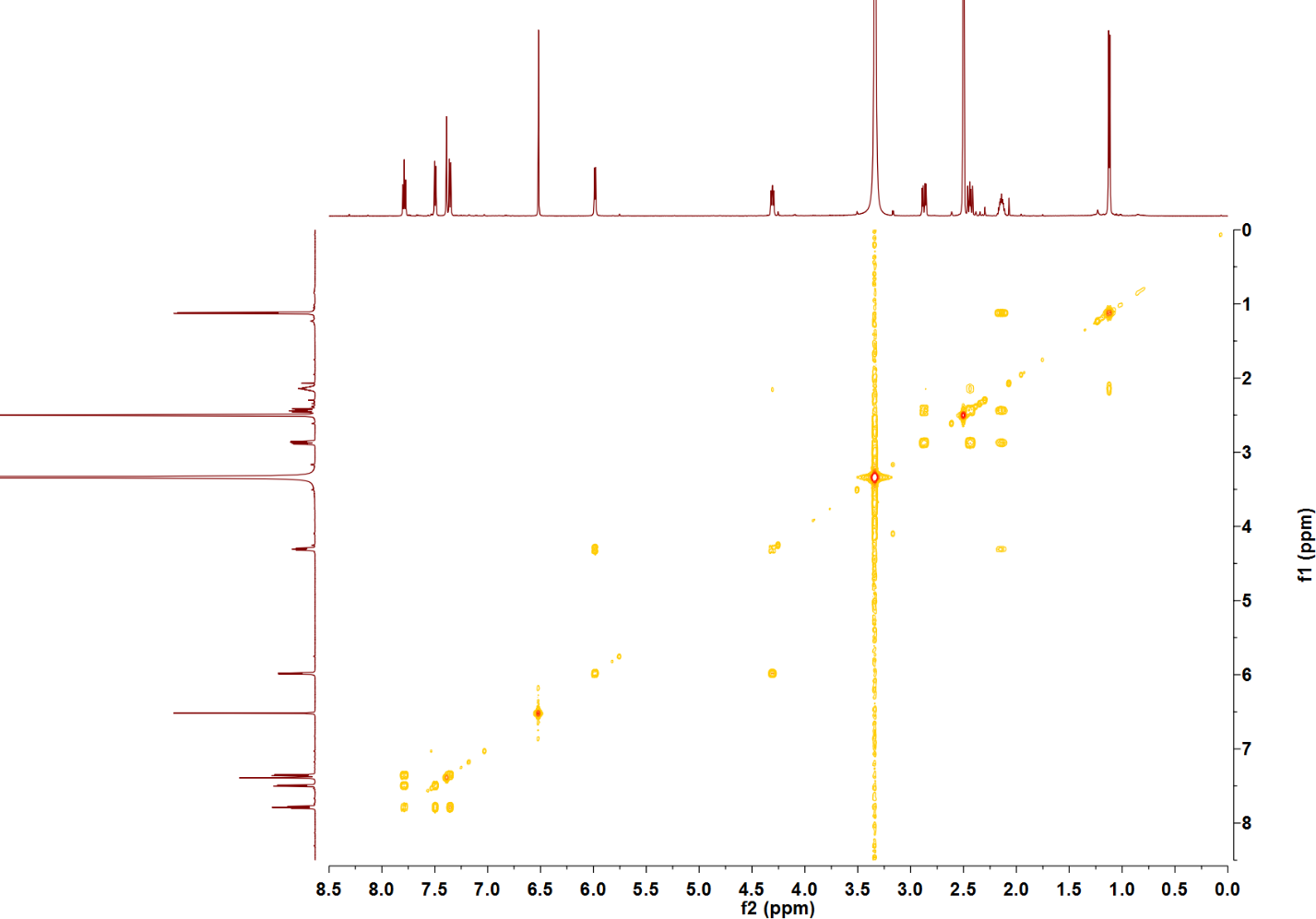


**Supplementary Fig. 65.** COSY spectrum of 6-hydroxyfujianmycin A_1_ (**7**) in DMSO-*d*_6_.


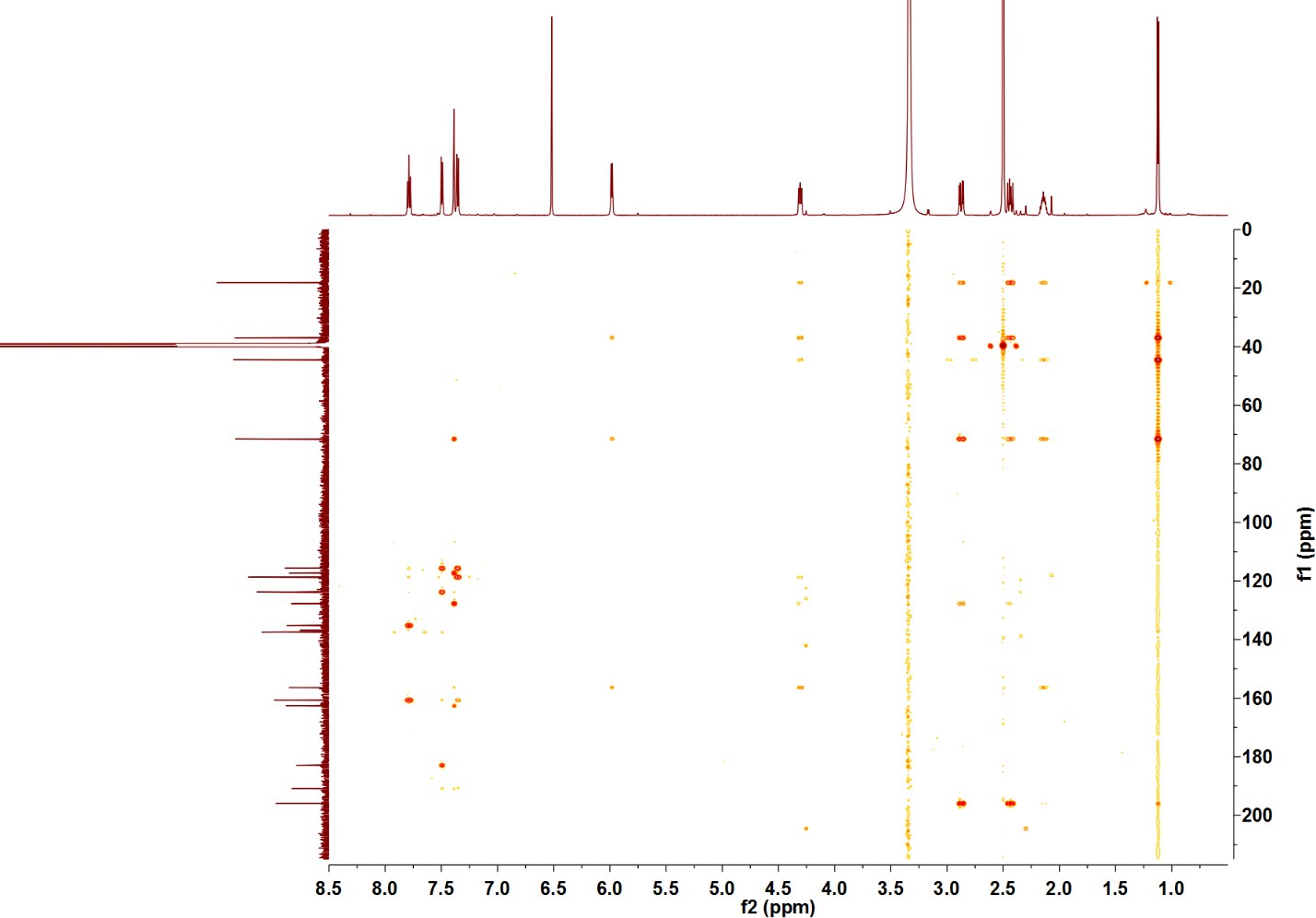


**Supplementary Fig. 66.** HMBC spectrum of 6-hydroxyfujianmycin A_1_ (**7**) in DMSO-*d*_6_.


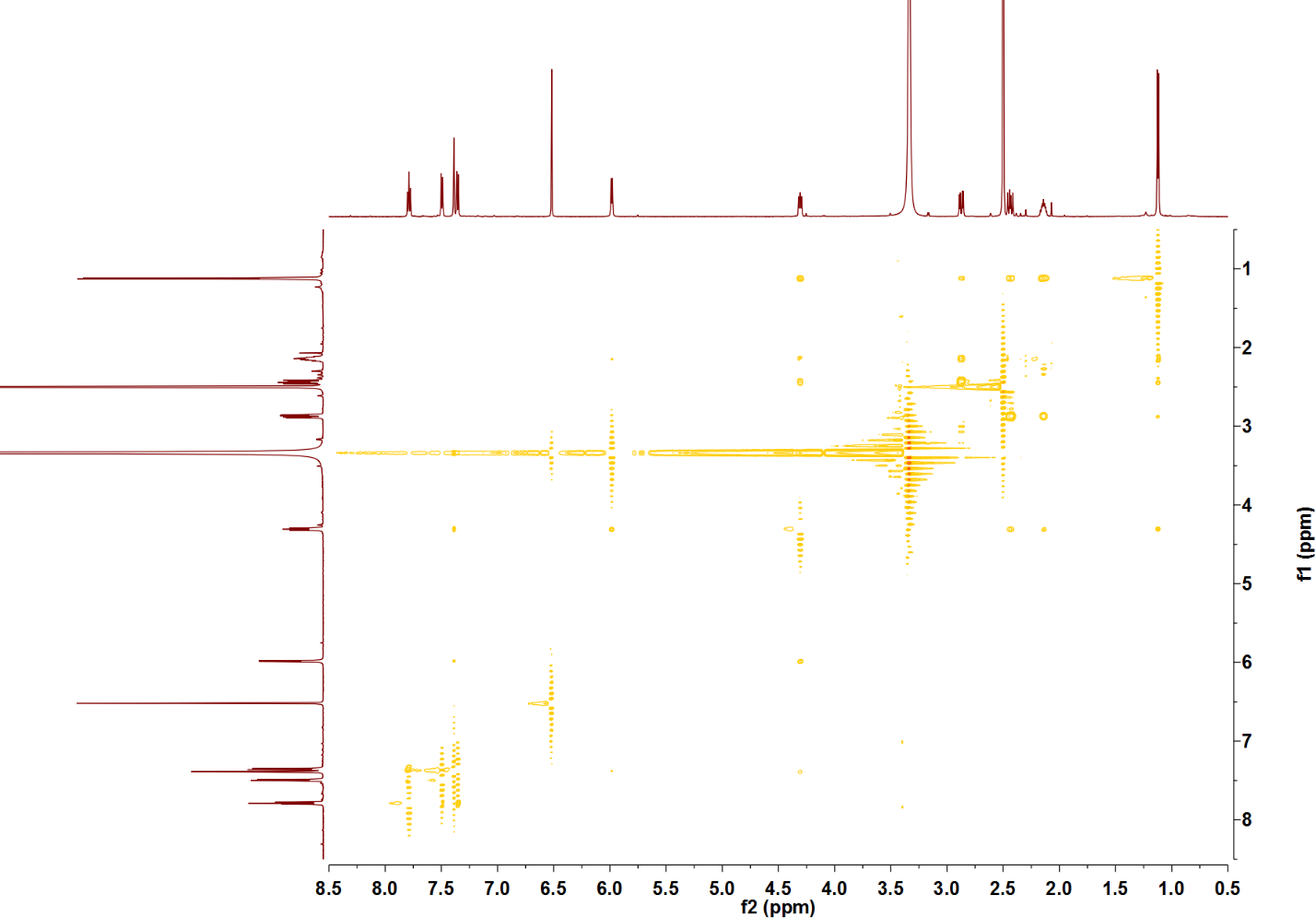


**Supplementary Fig. 67.** NOESY spectrum of 6-hydroxyfujianmycin A_1_ (**7**) DMSO-*d*_6_.


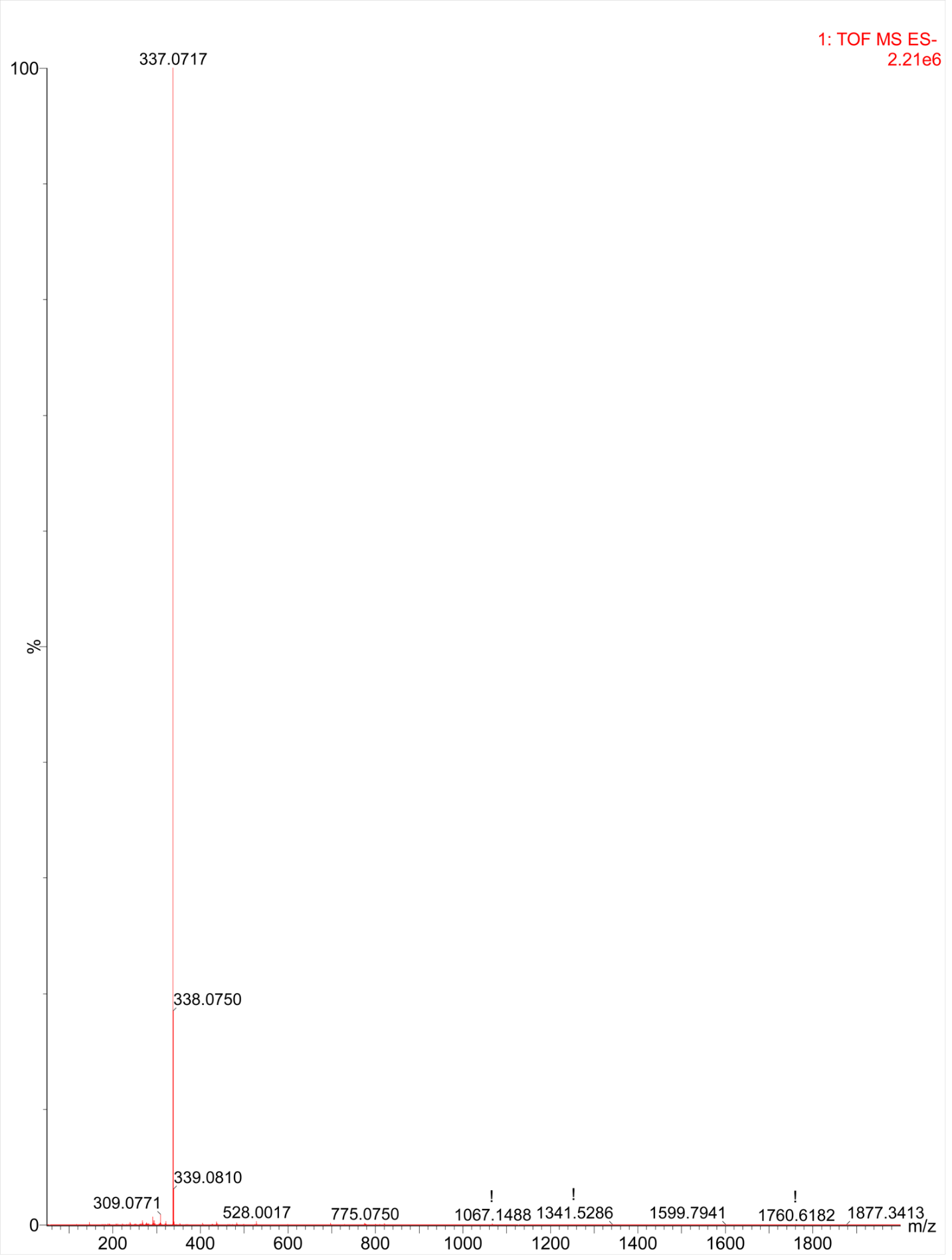


**Supplementary Fig. 68.** HRESIMS spectrum of 6-hydroxyfujianmycin A_2_ (**8**).


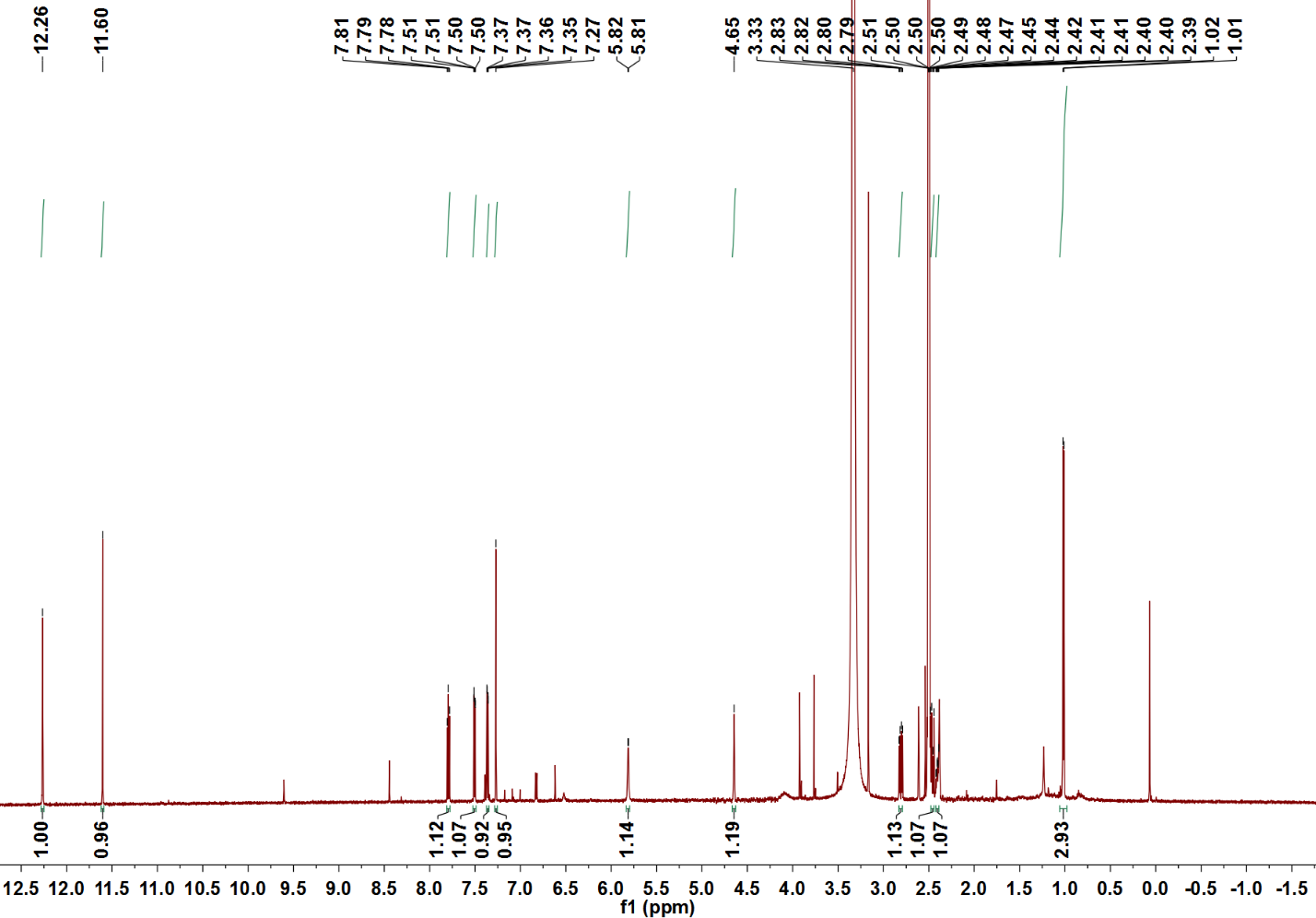


**Supplementary Fig. 69.** ^1^H NMR spectrum of 6-hydroxyfujianmycin A_2_ (**8**) in DMSO-*d*_6_.


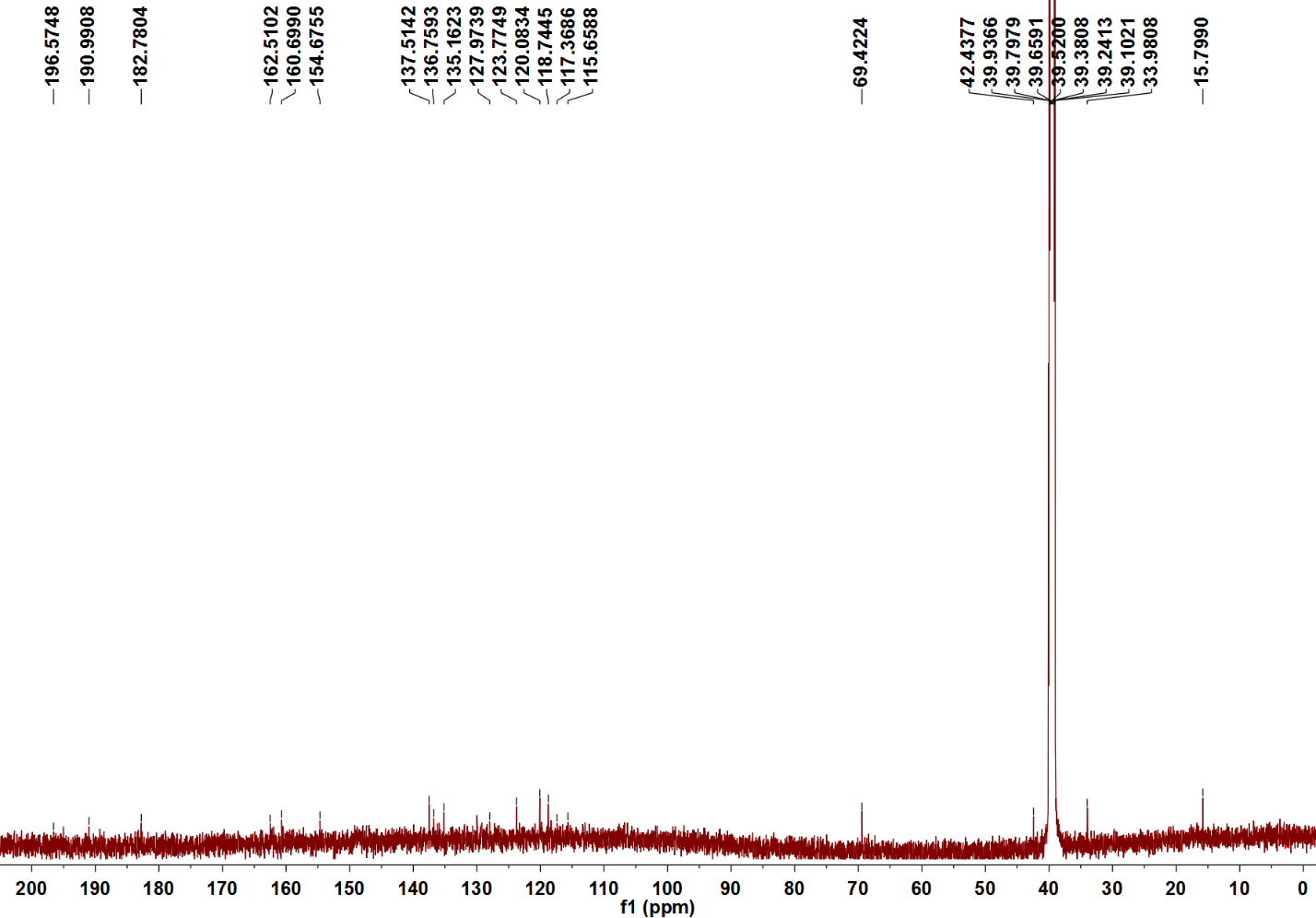


**Supplementary Fig. 70.** ^13^C NMR spectrum of 6-hydroxyfujianmycin A_2_ (**8**) in DMSO-*d*_6_.


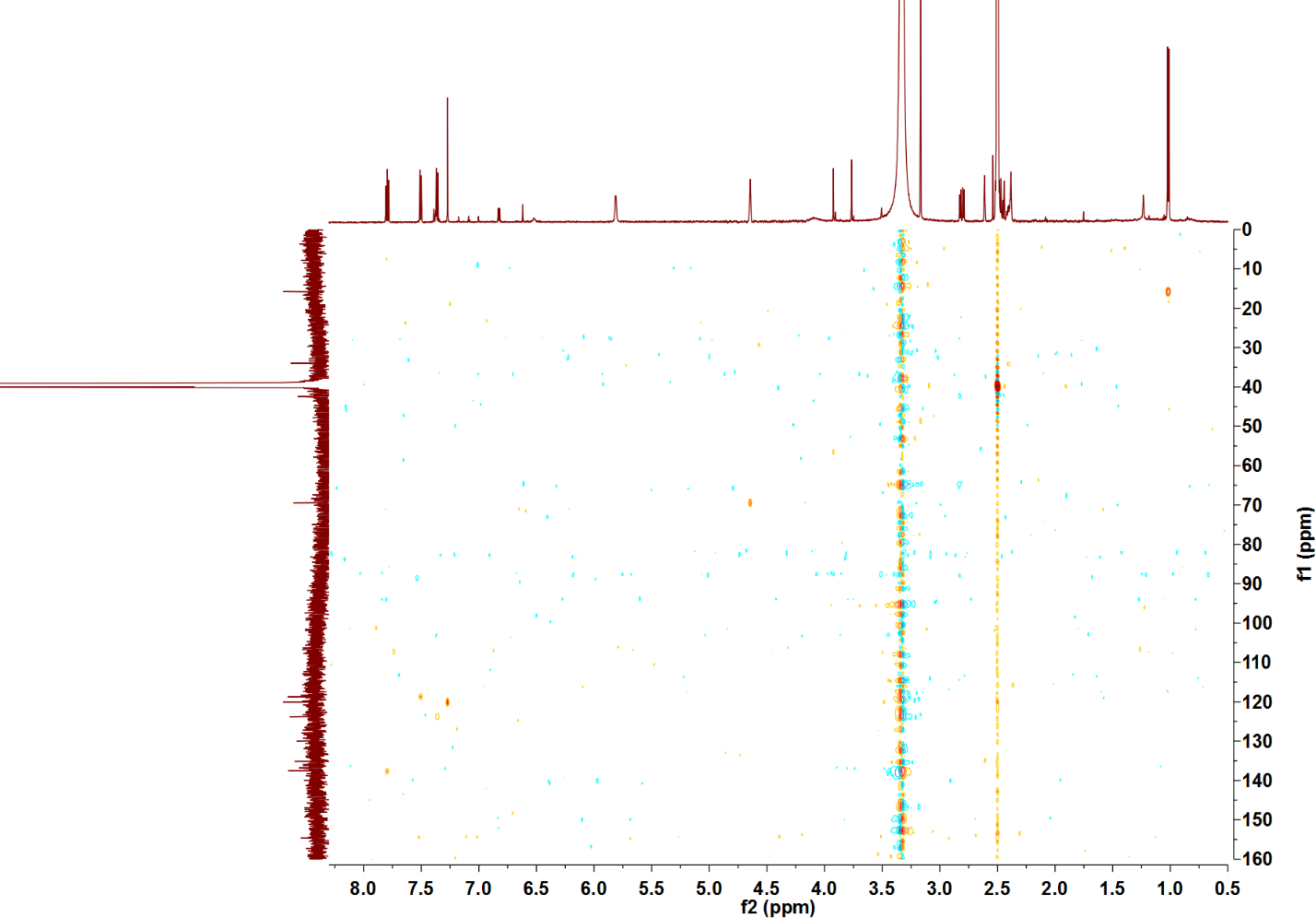


**Supplementary Fig. 71.** HSQC spectrum of 6-hydroxyfujianmycin A_2_ (**8**) in DMSO-*d*_6_.


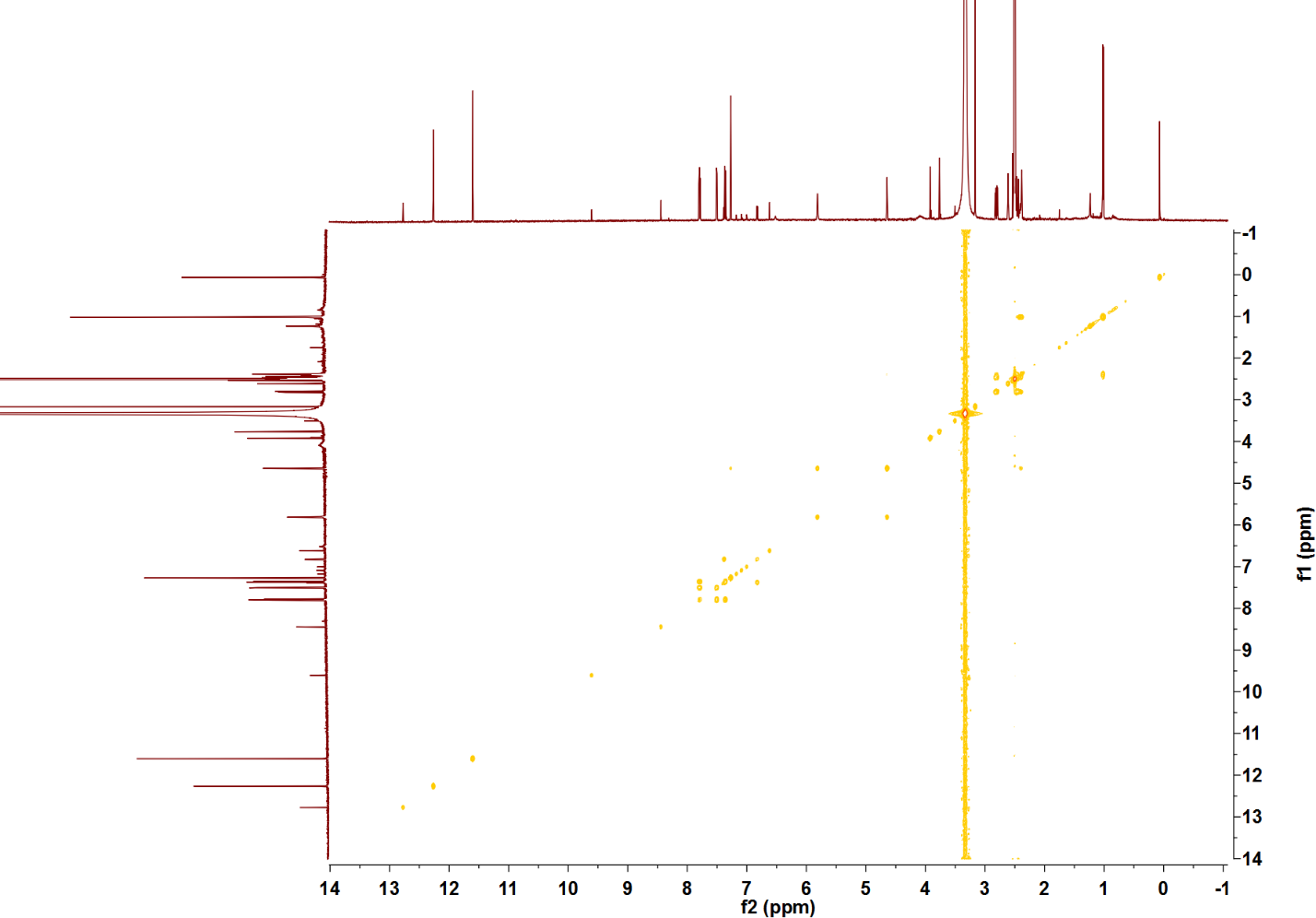


**Supplementary Fig. 72.** COSY spectrum of 6-hydroxyfujianmycin A_2_ (**8**) in DMSO-*d*_6_.


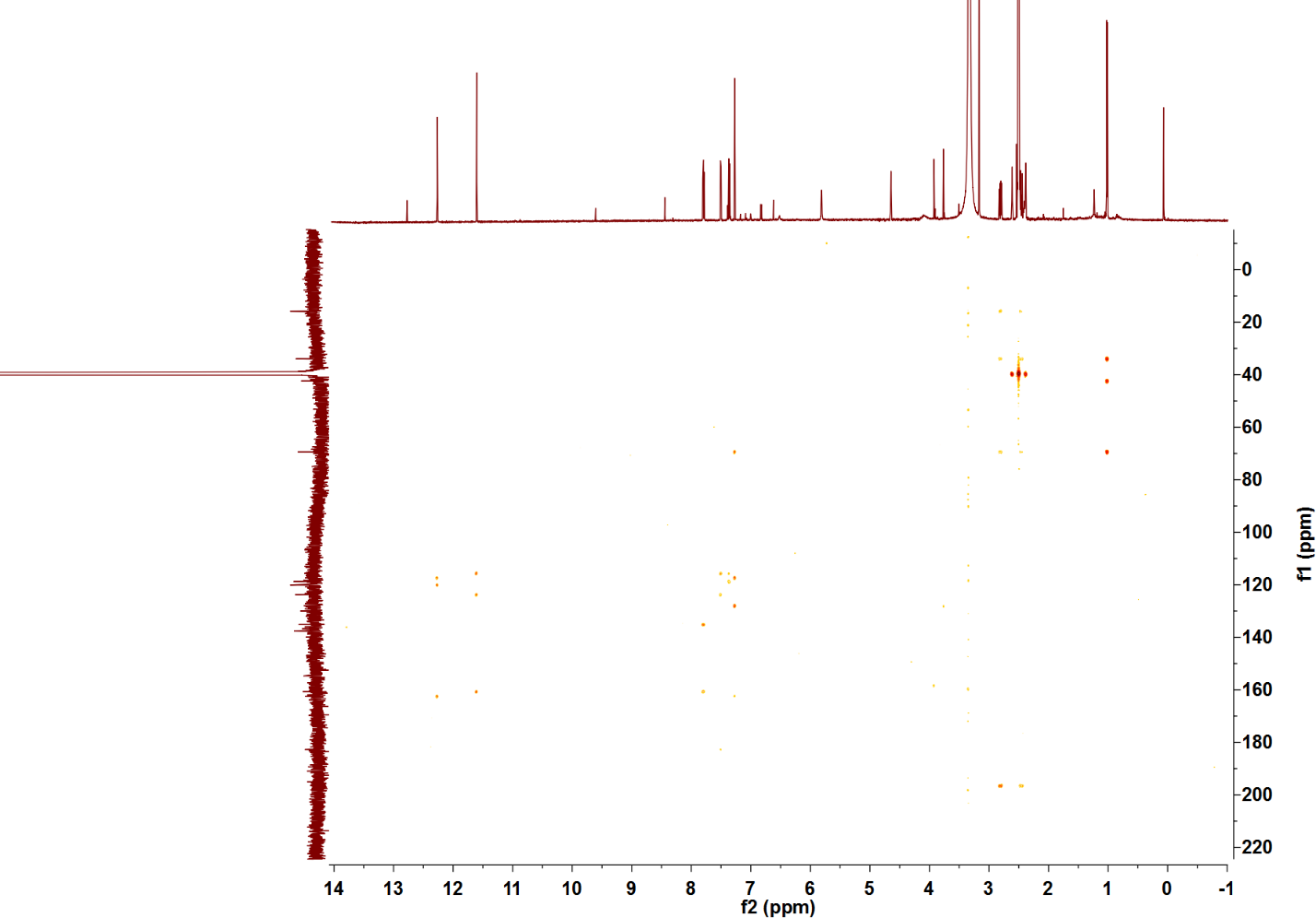


**Supplementary Fig. 73.** HMBC spectrum of 6-hydroxyfujianmycin A_2_ (**8**) in DMSO-*d*_6_.


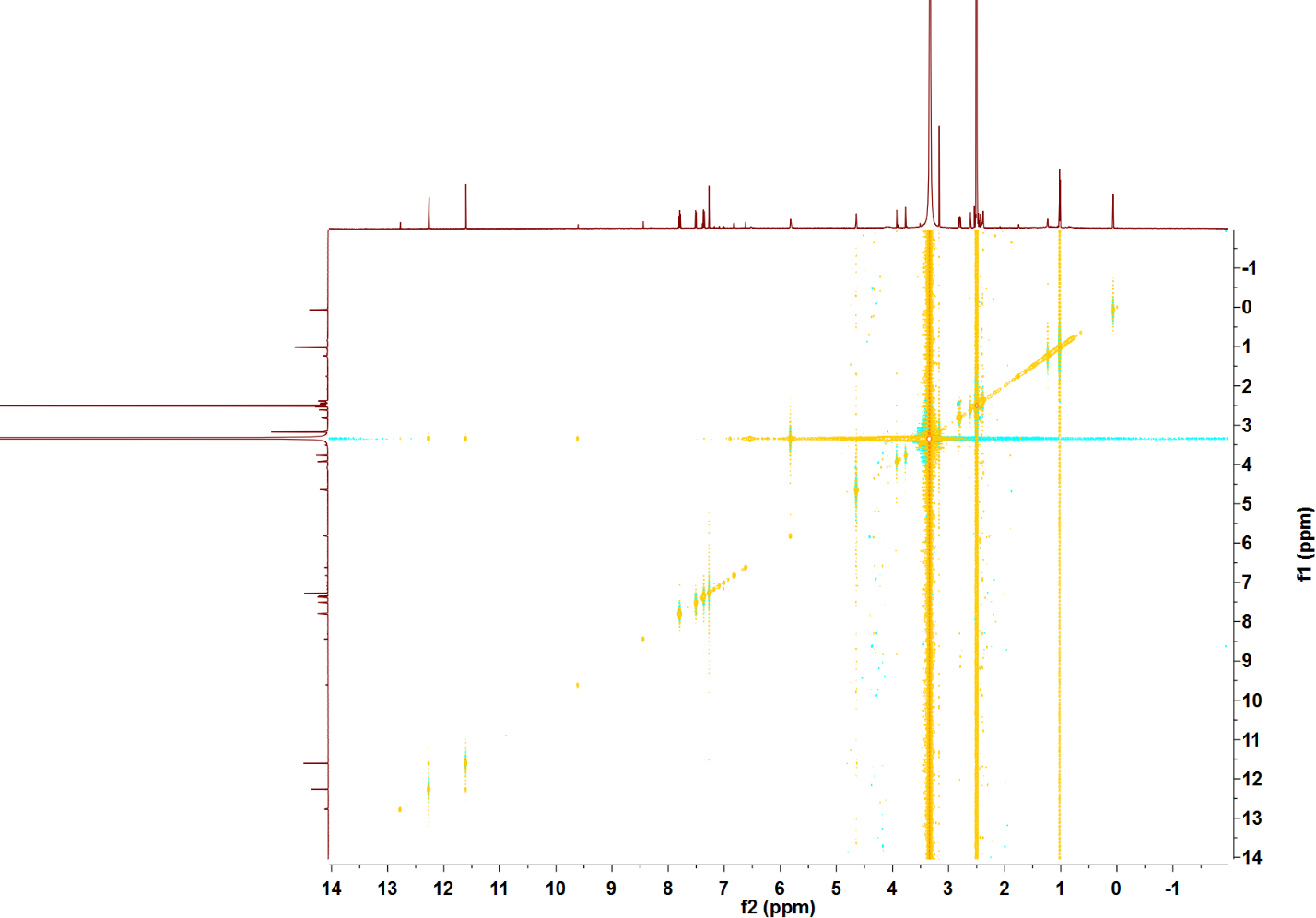


**Supplementary Fig. 74.** NOESY spectrum of 6-hydroxyfujianmycin A_2_ (**8**) DMSO-*d*_6_.


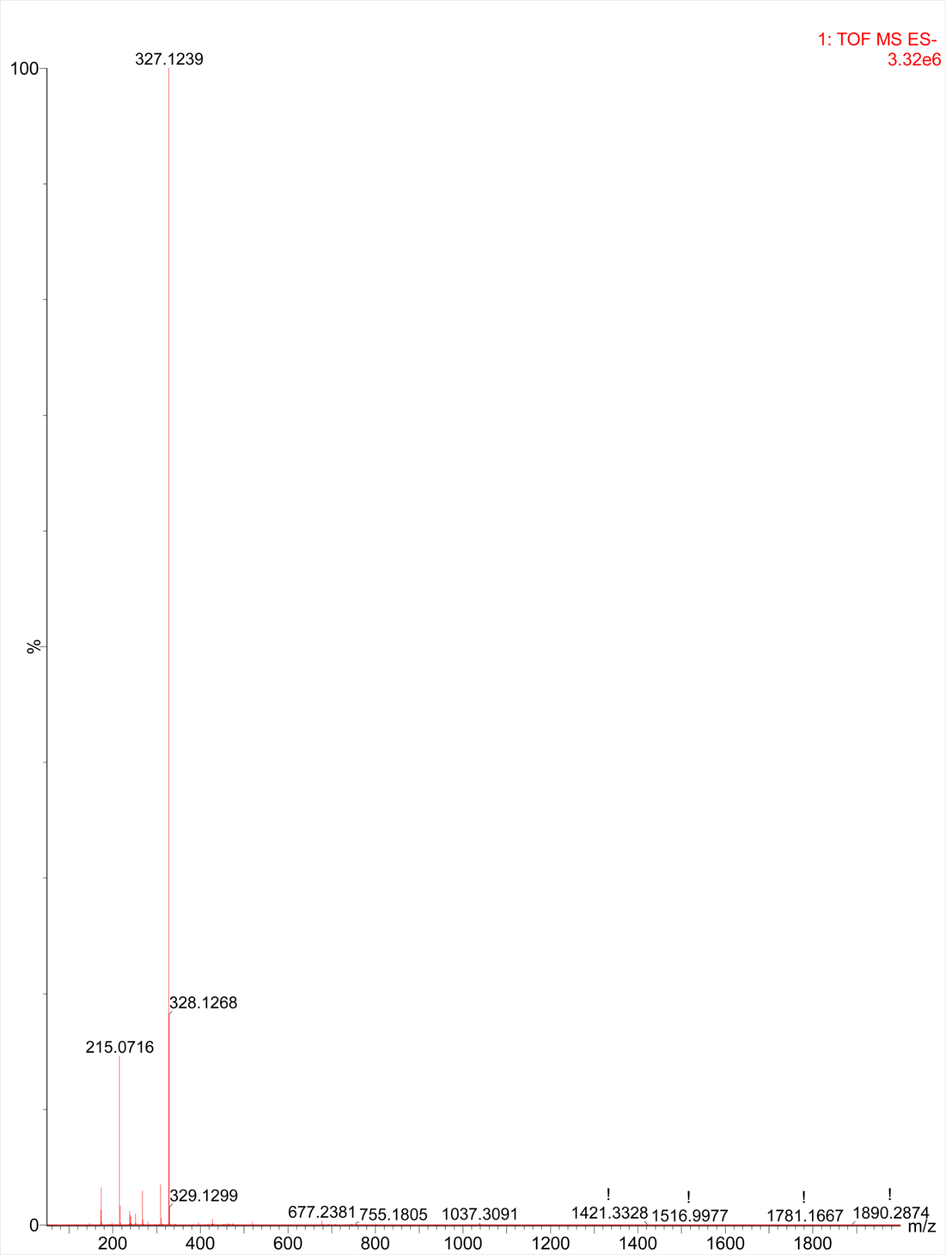


**Supplementary Fig. 75.** HRESIMS spectrum of 2,3-*seco*-prejadomycin (**9**).


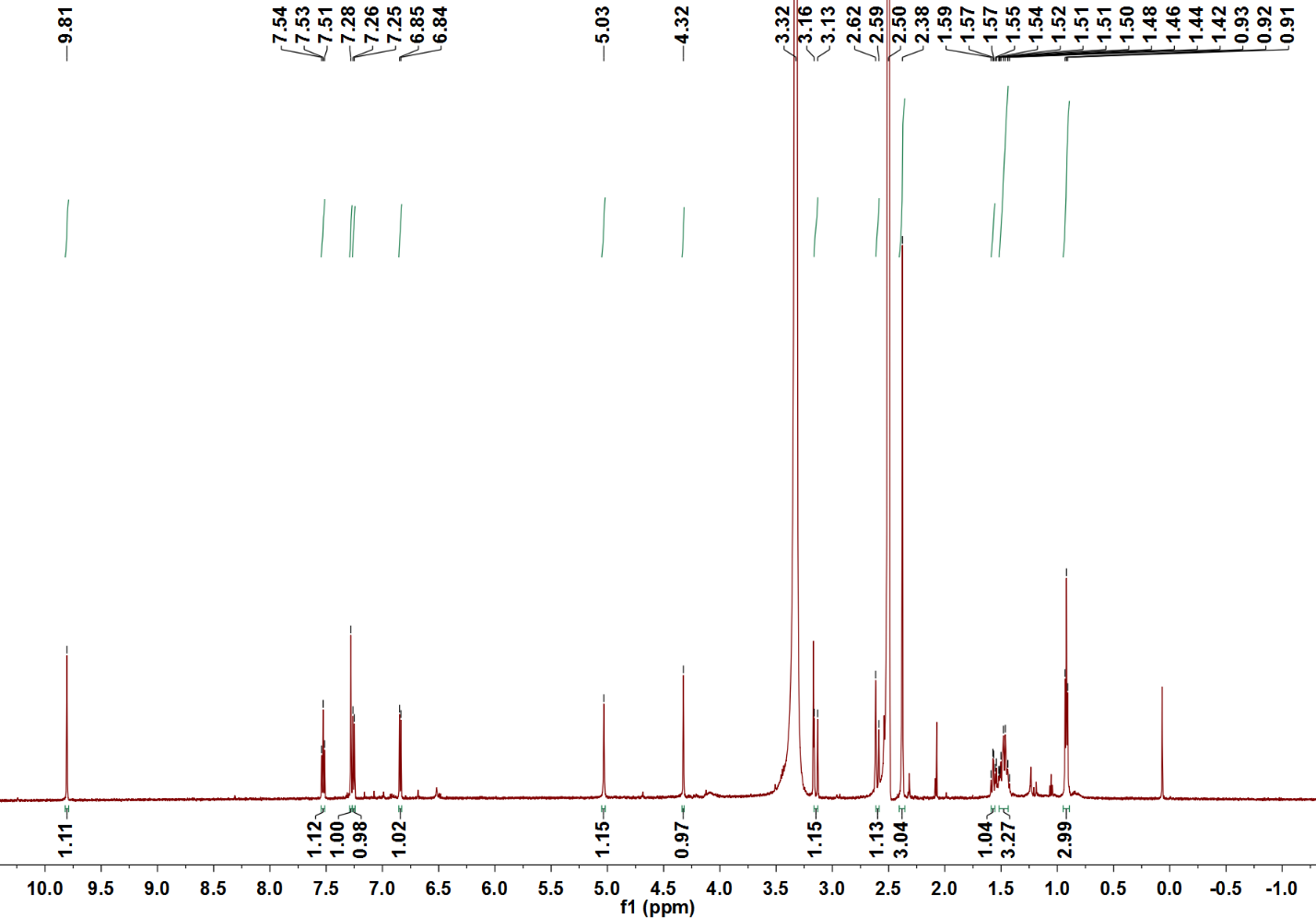


**Supplementary Fig. 76.** ^1^H NMR spectrum of 2,3-*seco*-prejadomycin (**9**) in DMSO-*d*_6_.


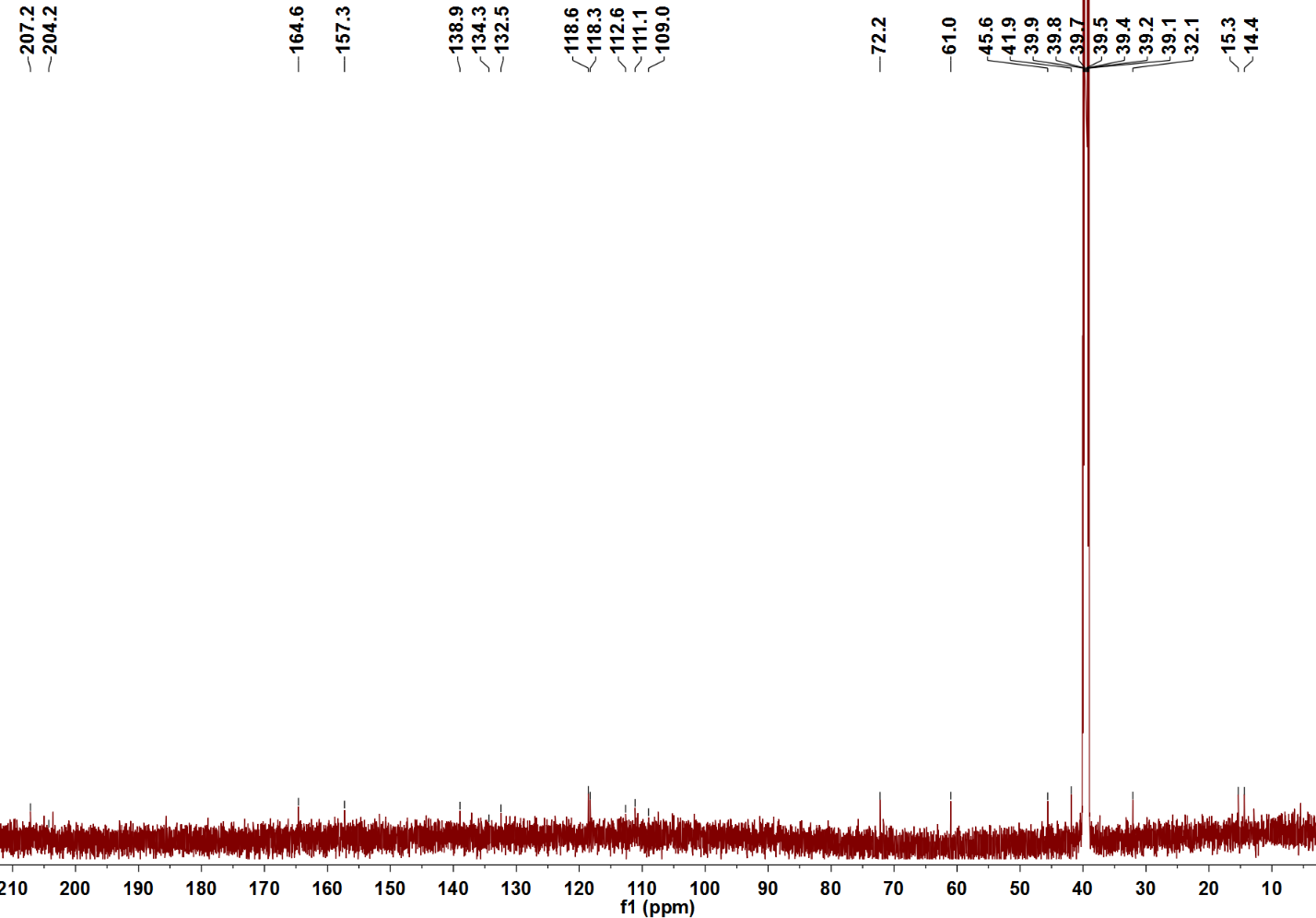


**Supplementary Fig. 77.** ^13^C NMR spectrum of 2,3-*seco*-prejadomycin (**9**) in DMSO-*d*_6_.


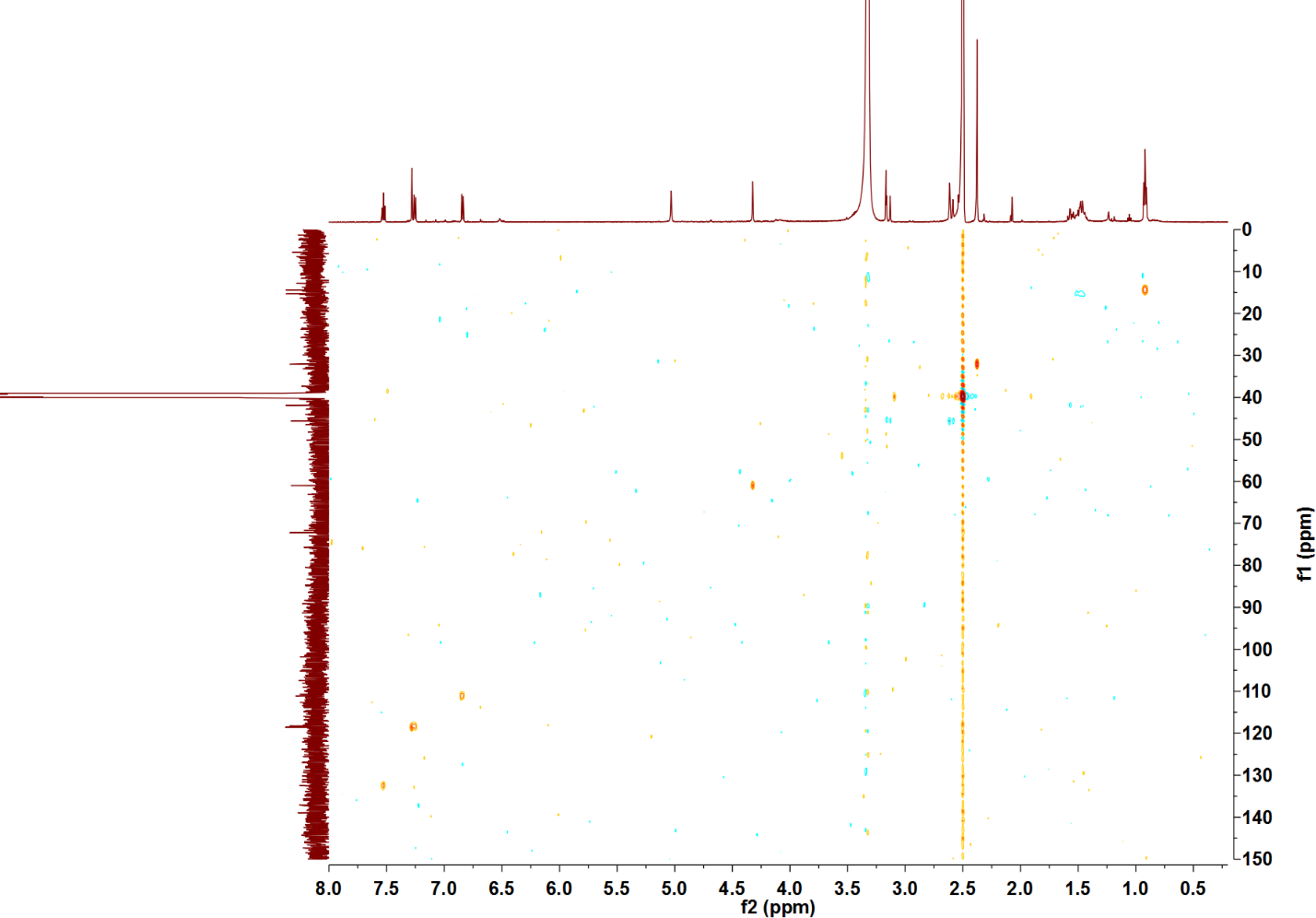


**Supplementary Fig. 78.** HSQC spectrum of 2,3-*seco*-prejadomycin (**9**) in DMSO-*d*_6_.


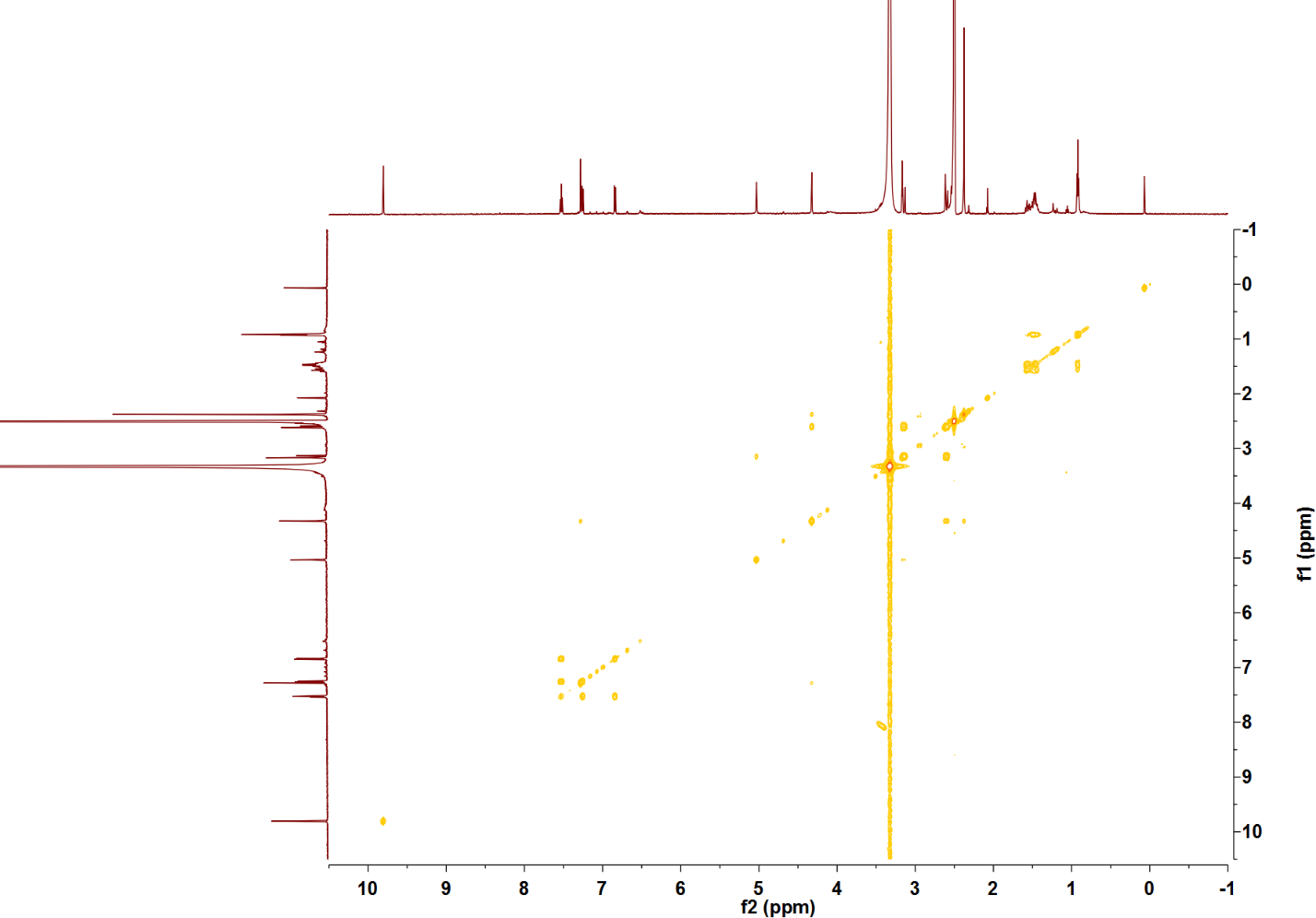


**Supplementary Fig. 79.** COSY spectrum of 2,3-*seco*-prejadomycin (**9**) in DMSO-*d*_6_.


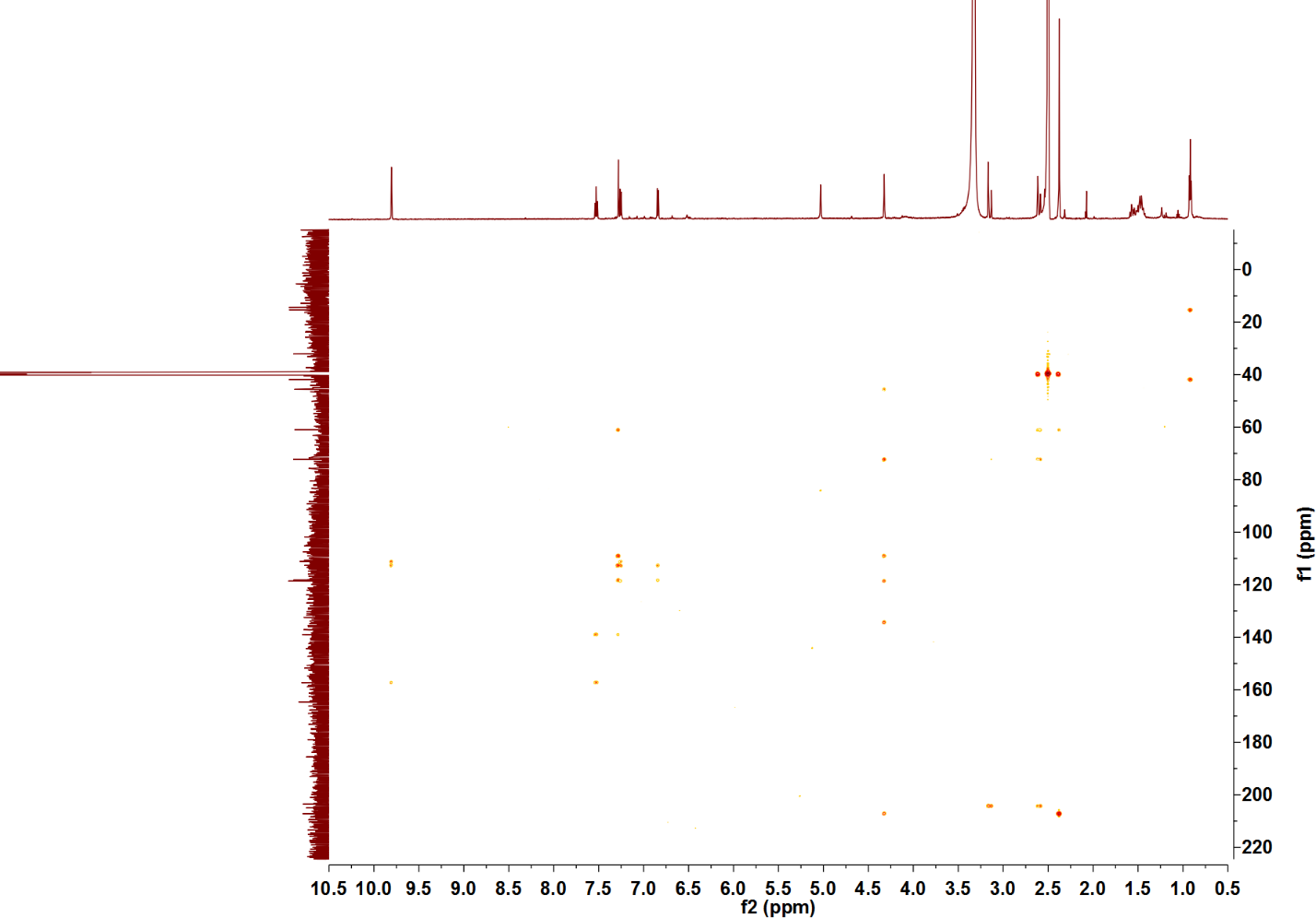


**Supplementary Fig. 80.** HMBC spectrum of 2,3-*seco*-prejadomycin (**9**) in DMSO-*d*_6_.


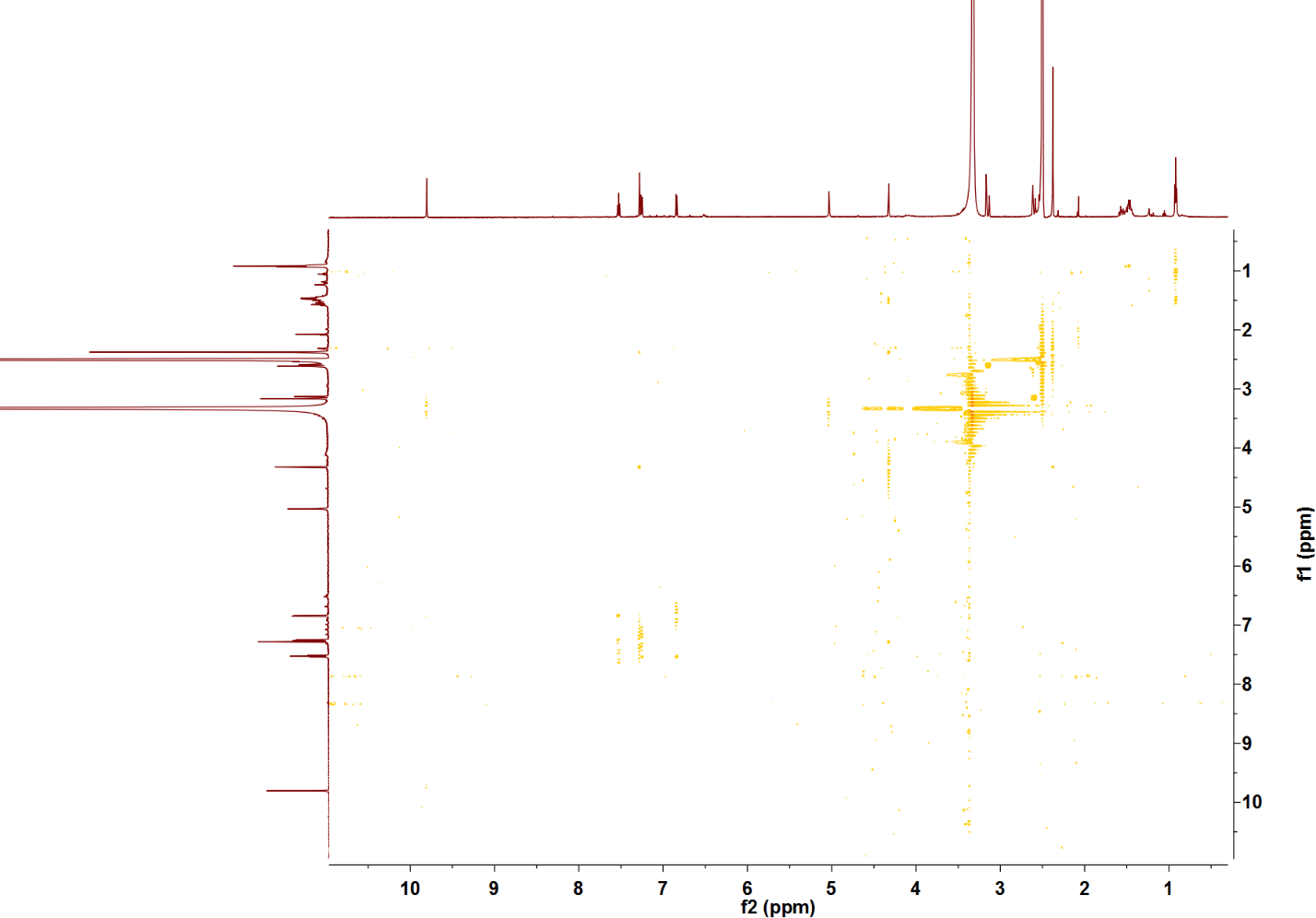


**Supplementary Fig. 81.** NOESY spectrum of 2,3-*seco*-prejadomycin (**9**) DMSO-*d*_6_.


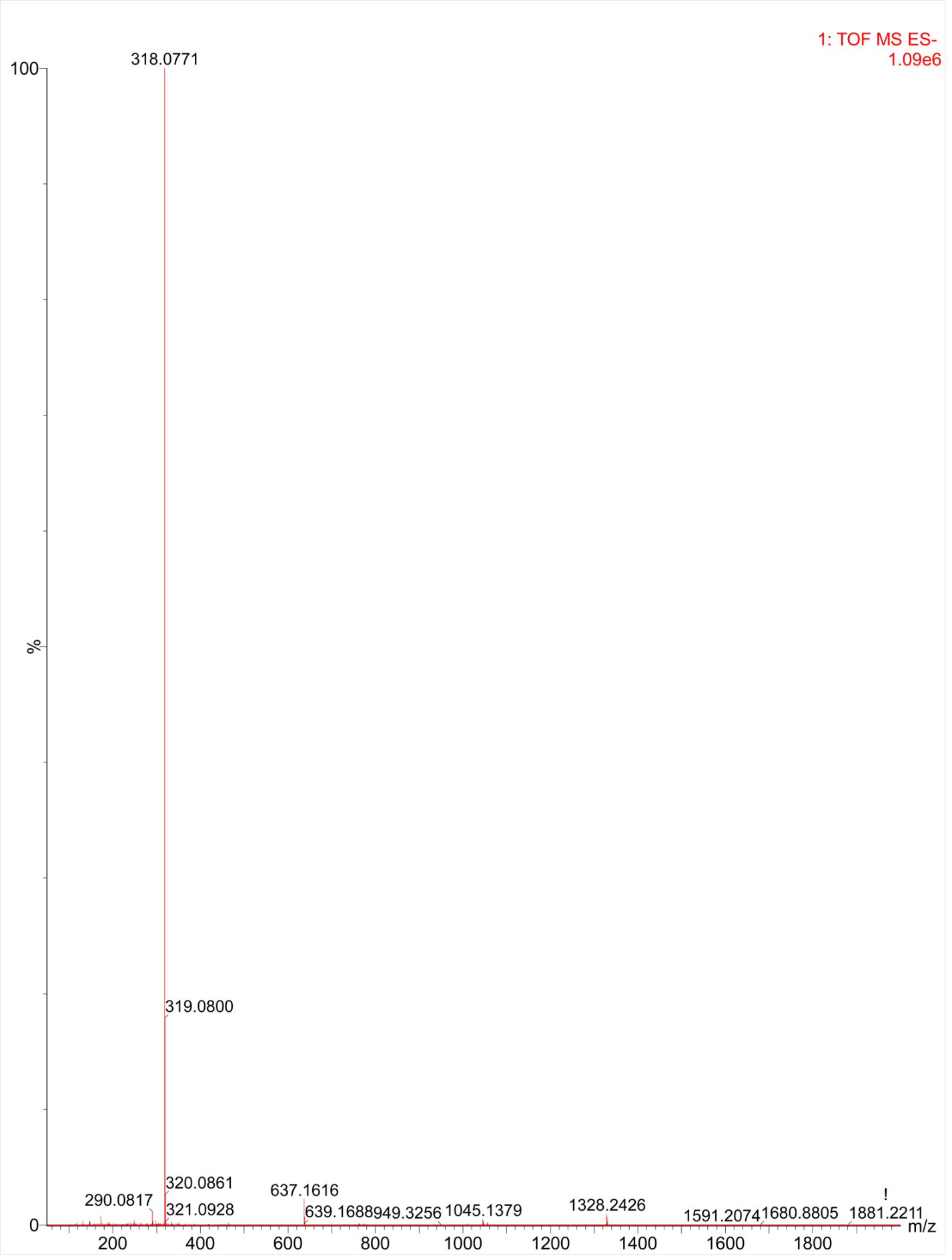


**Supplementary Fig. 82.** HRESIMS spectrum of pregilvocarcin W (**10**).


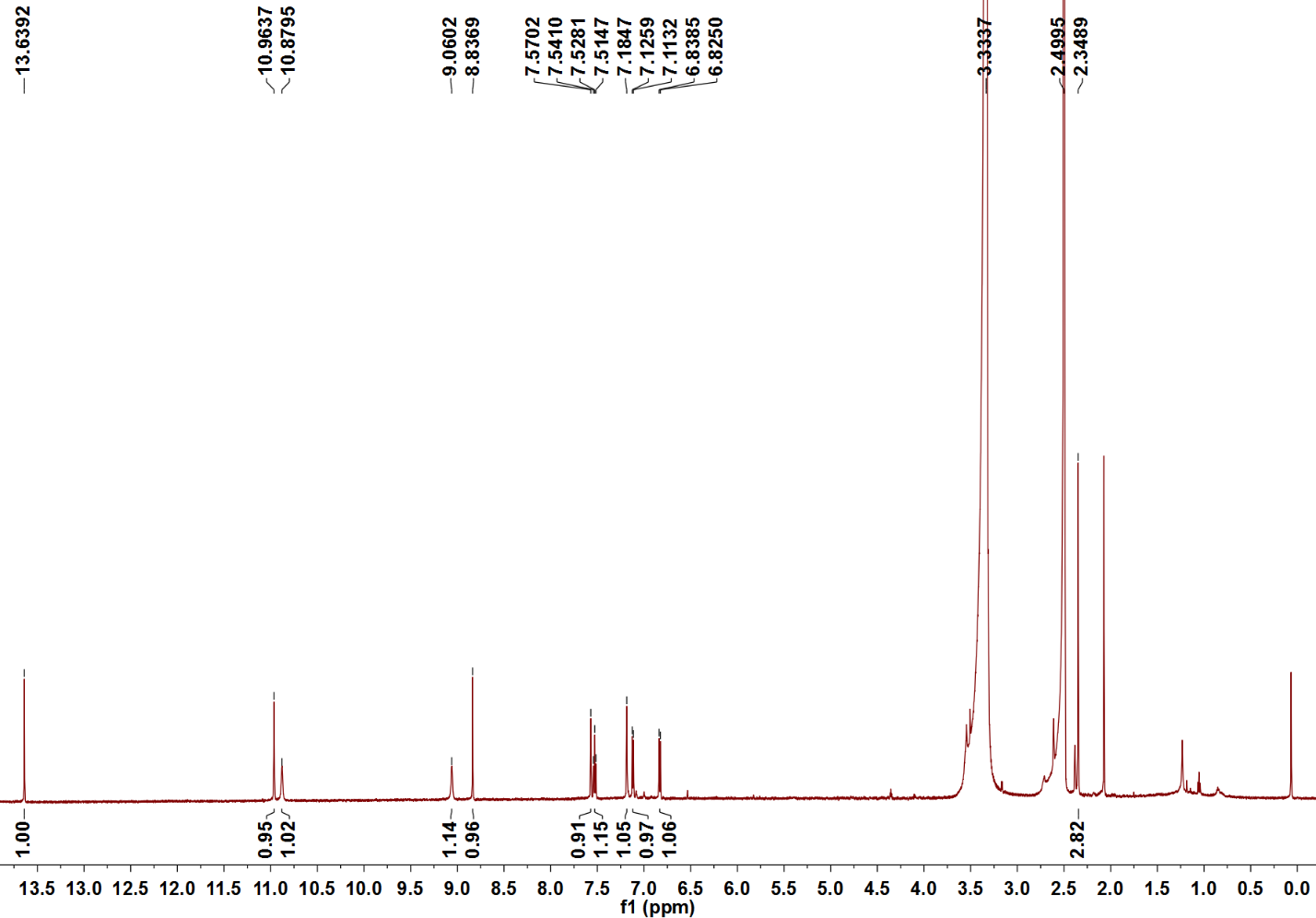


**Supplementary Fig. 83.** ^1^H NMR spectrum of pregilvocarcin W (**10**) in DMSO-*d*_6_.


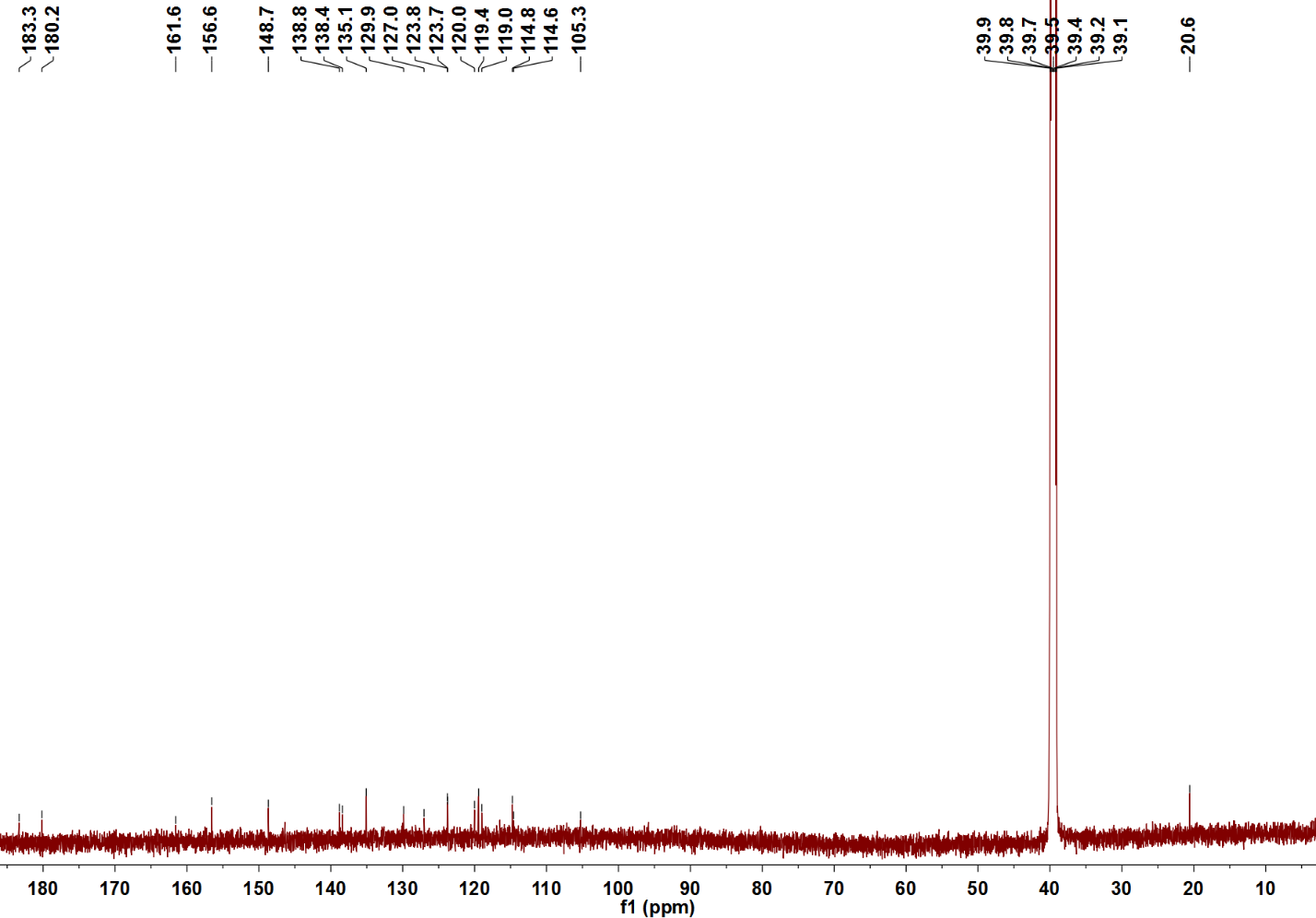


**Supplementary Fig. 84.** ^13^C NMR spectrum of pregilvocarcin W (**10**) in DMSO-*d*_6_.


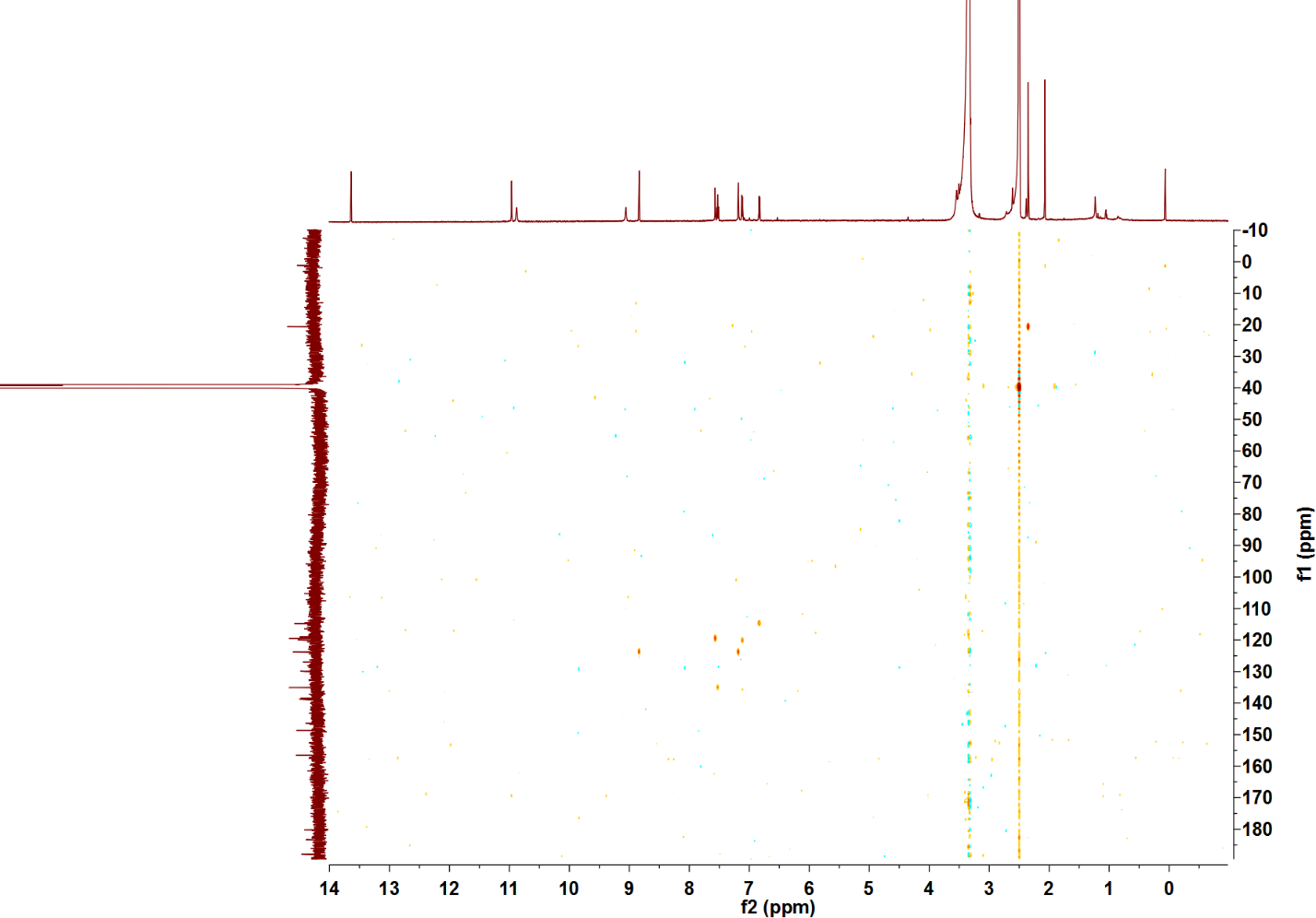


**Supplementary Fig. 85.** HSQC spectrum of pregilvocarcin W (**10**) in DMSO-*d*_6_.


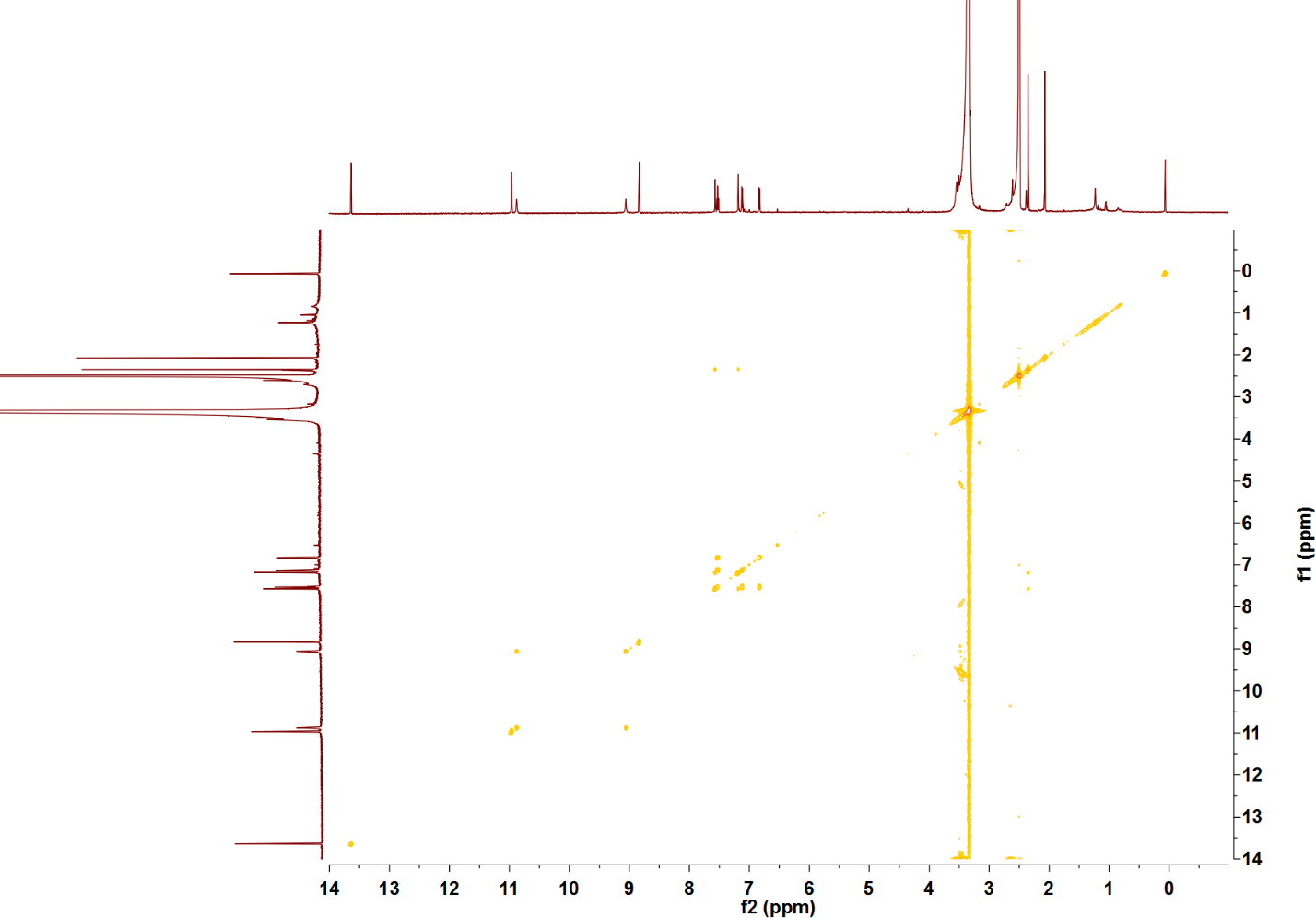


**Supplementary Fig. 86.** COSY spectrum of pregilvocarcin W (**10**) in DMSO-*d*_6_.


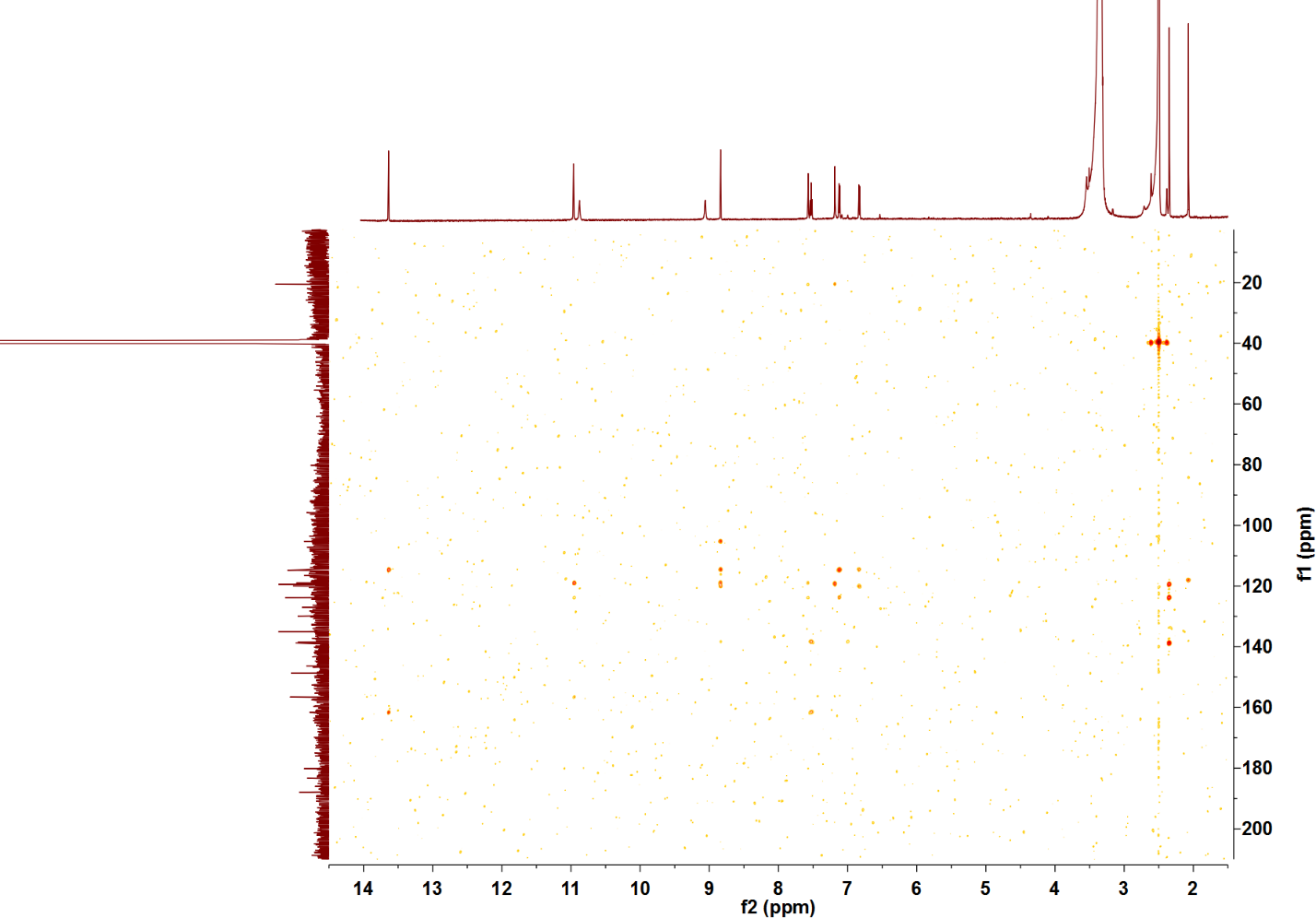


**Supplementary Fig. 87.** HMBC spectrum of pregilvocarcin W (**10**) in DMSO-*d*_6_.


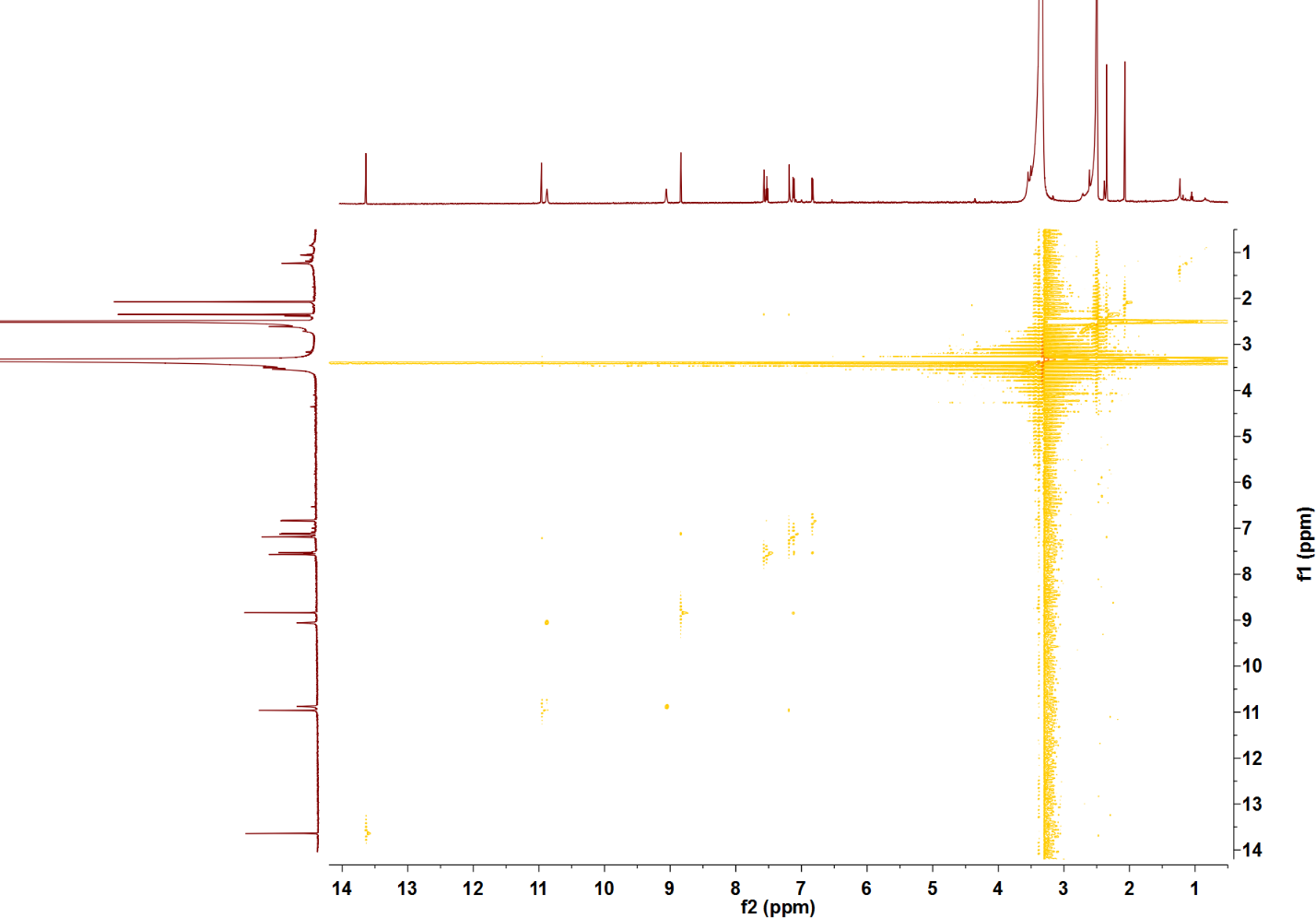


**Supplementary Fig. 88.** NOESY spectrum of pregilvocarcin W (**10**) DMSO-*d*_6_.


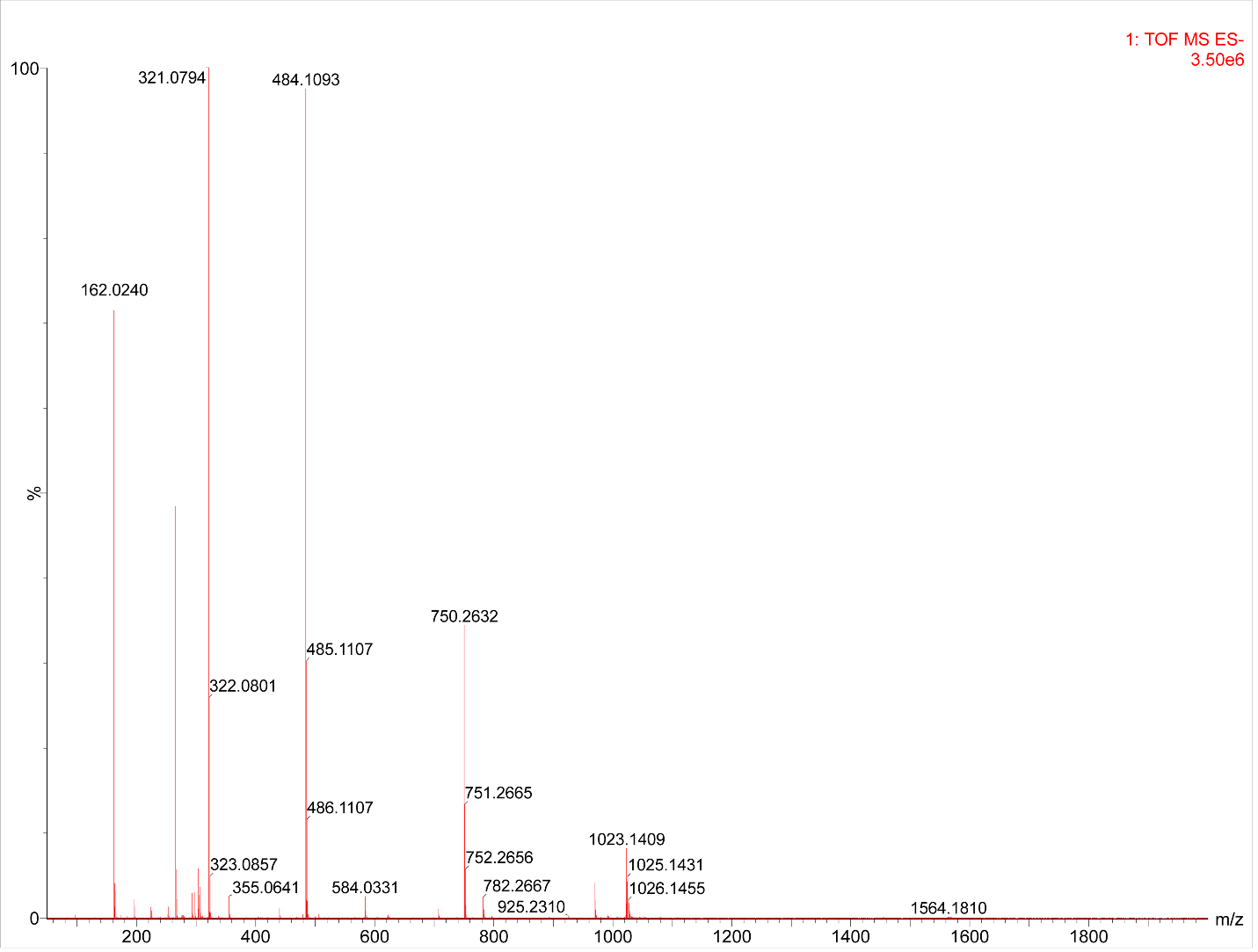


**Supplementary Fig. 89.** HRESIMS spectrum of *N*-acetylcysteinmansoquinone (**11**).


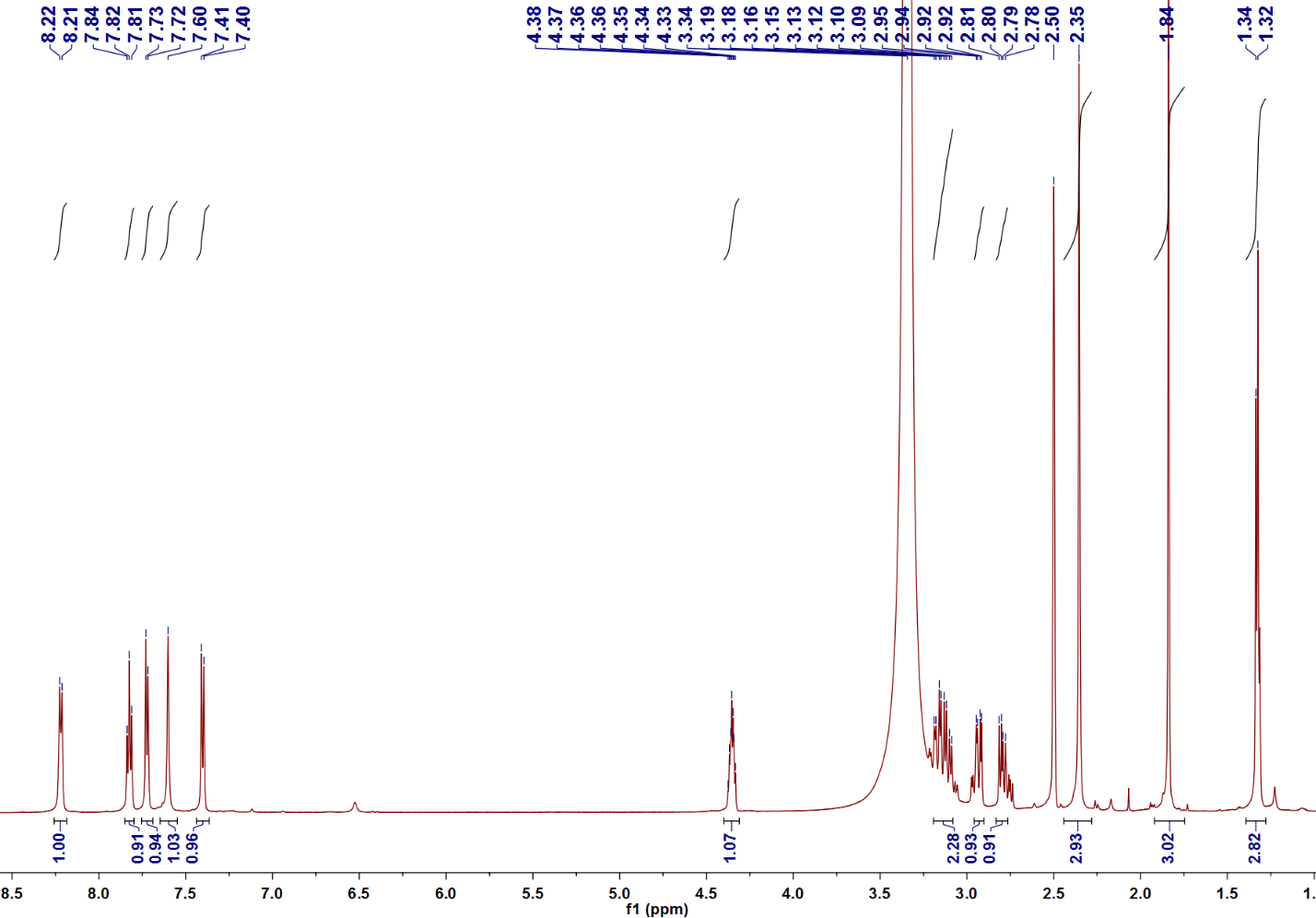


**Supplementary Fig. 90.** ^1^H NMR spectrum of *N*-acetylcysteinmansoquinone (**11**) in DMSO-*d*_6_.


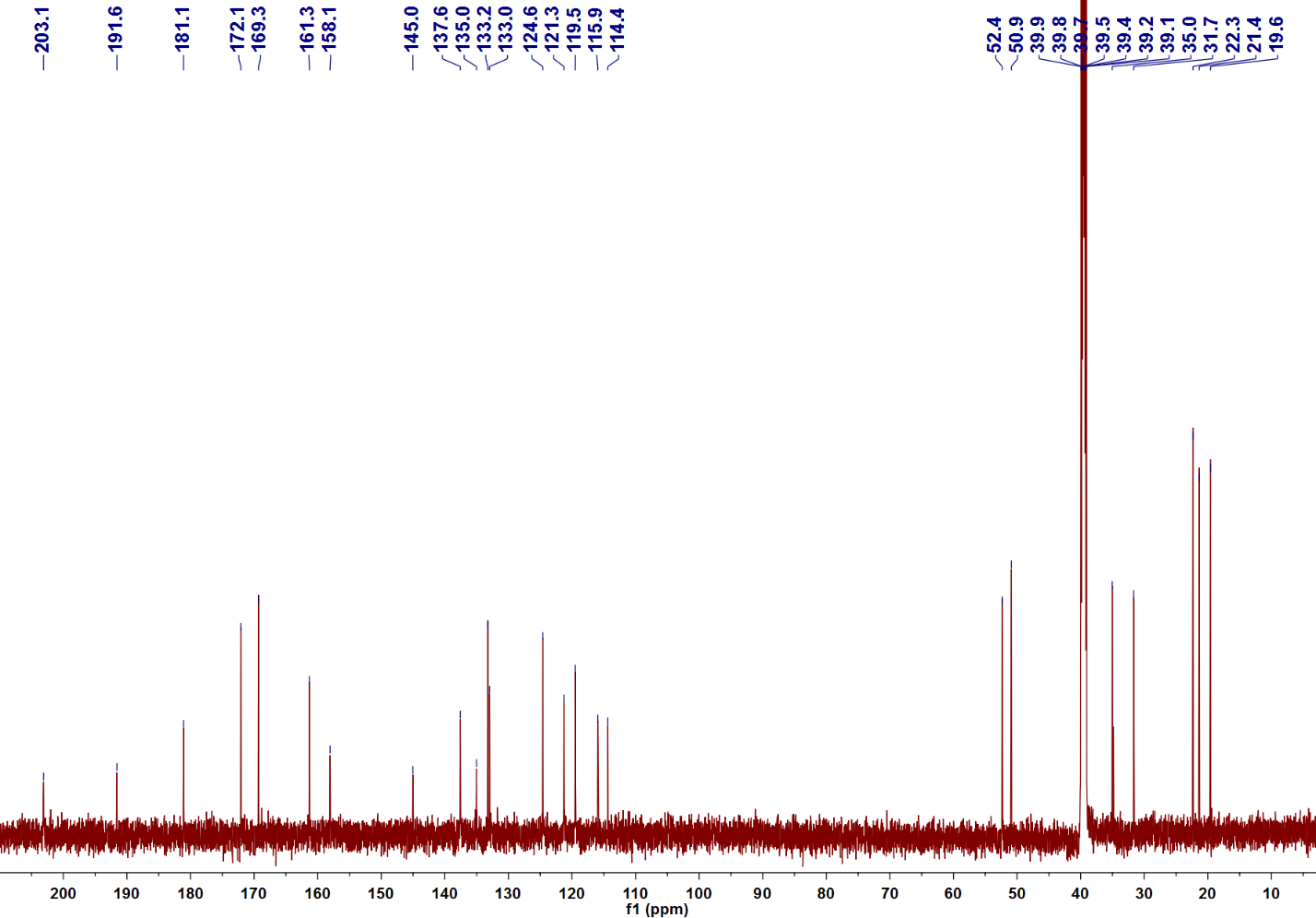


**Supplementary Fig. 91.** ^13^C NMR spectrum of *N*-acetylcysteinmansoquinone (**11**) in DMSO-*d*_6_.


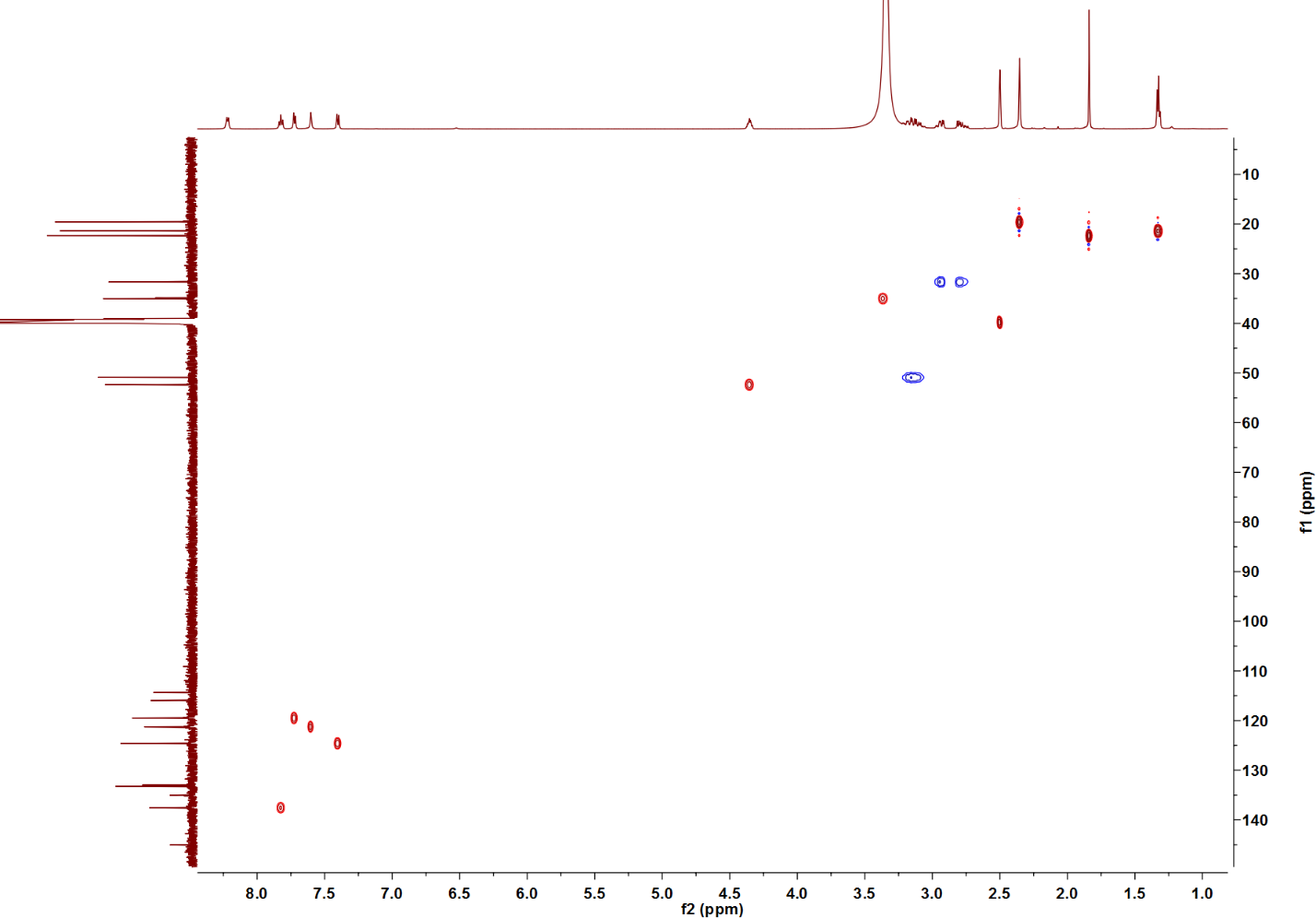


**Supplementary Fig. 92.** HSQC spectrum of *N*-acetylcysteinmansoquinone (**11**) in DMSO-*d*_6_.


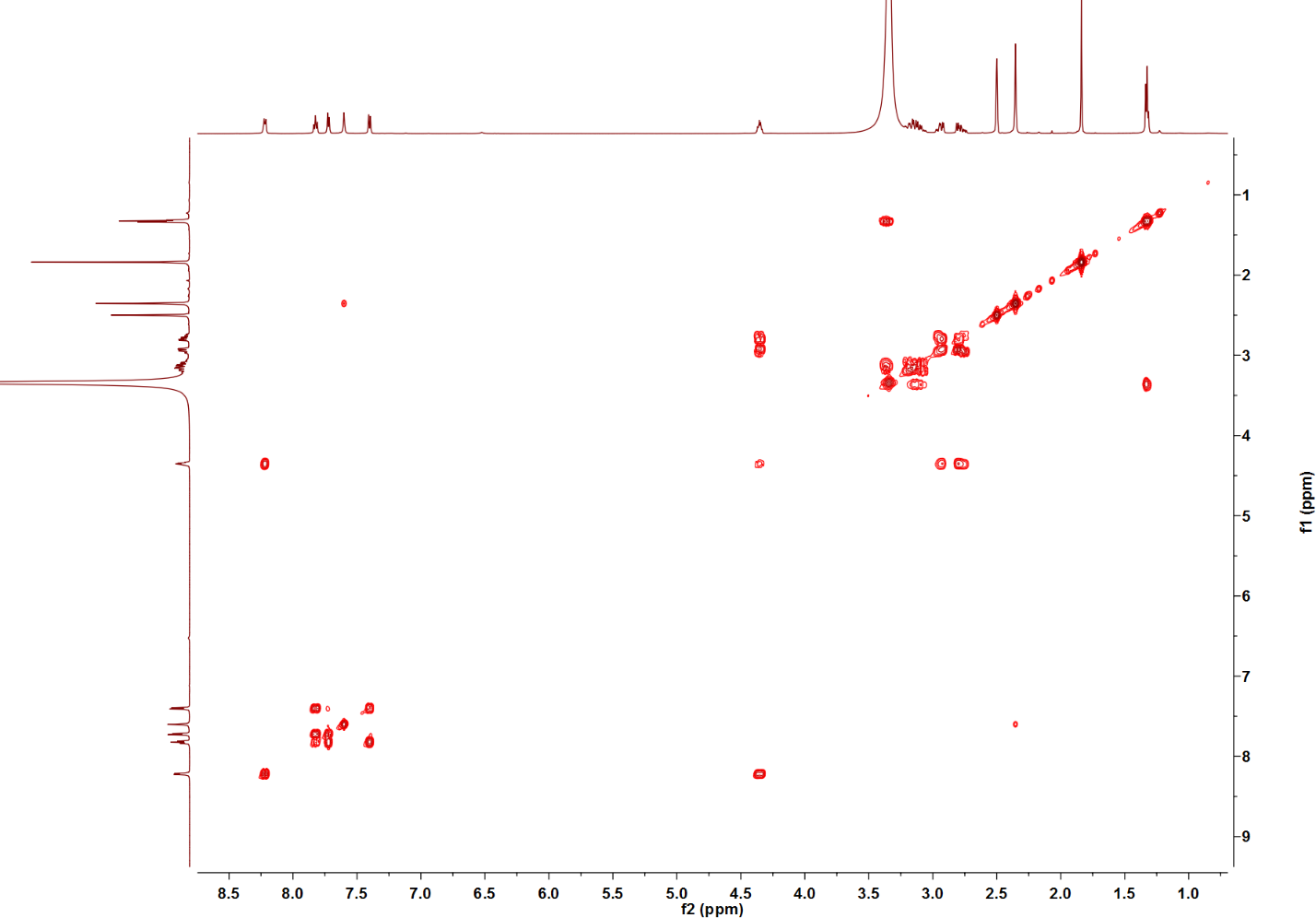


**Supplementary Fig. 93.** COSY spectrum of *N*-acetylcysteinmansoquinone (**11**) in DMSO-*d*_6_.


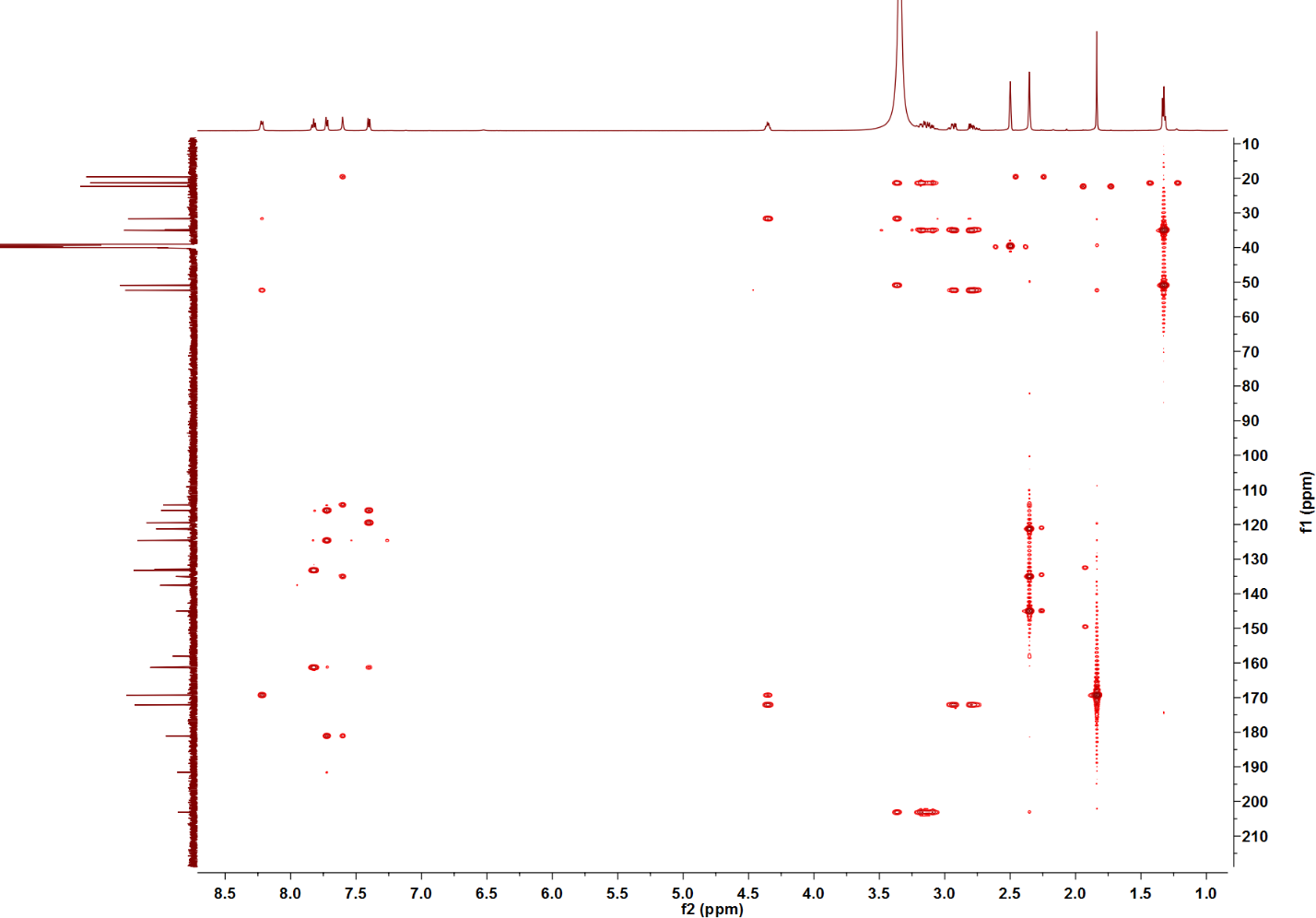


**Supplementary Fig. 94.** HMBC spectrum of *N*-acetylcysteinmansoquinone (**11**) in DMSO-*d*_6_.


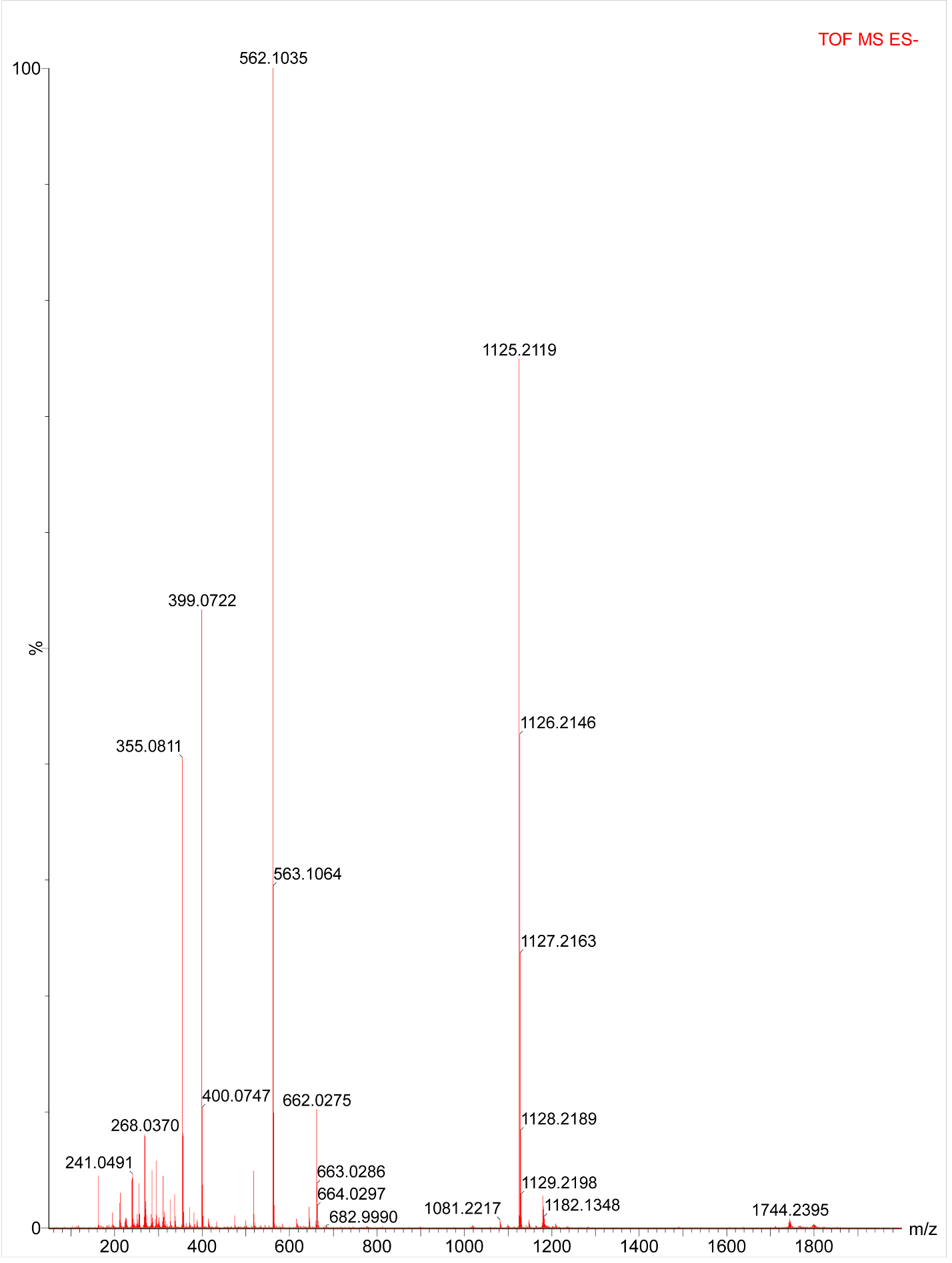


**Supplementary Fig. 95.** HRESIMS spectrum of *N*-acetylcysteingriseusin (**12**).


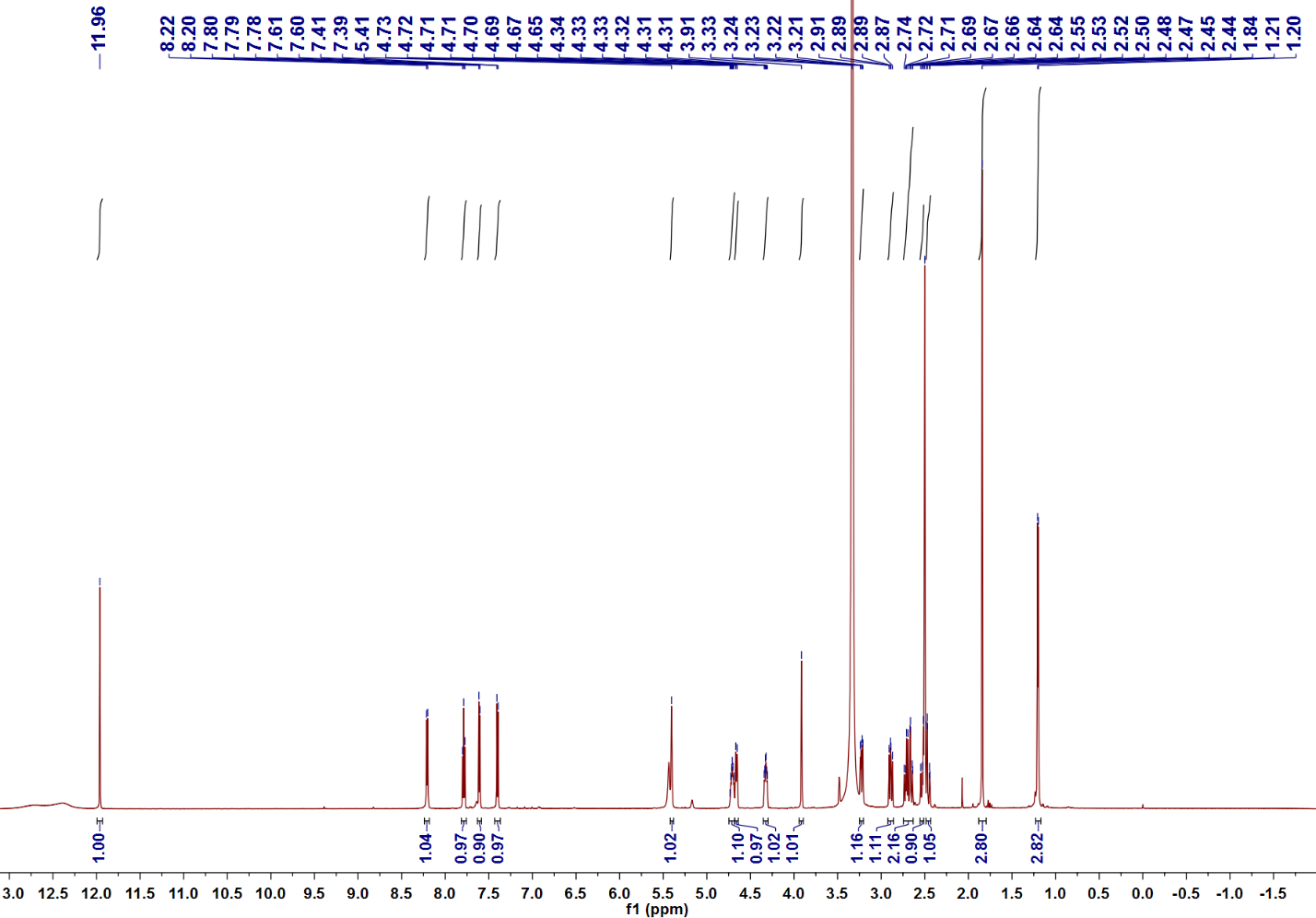


**Supplementary Fig. 96.** ^1^H NMR spectrum of *N*-acetylcysteingriseusin (**12**) in DMSO-*d*_6_.


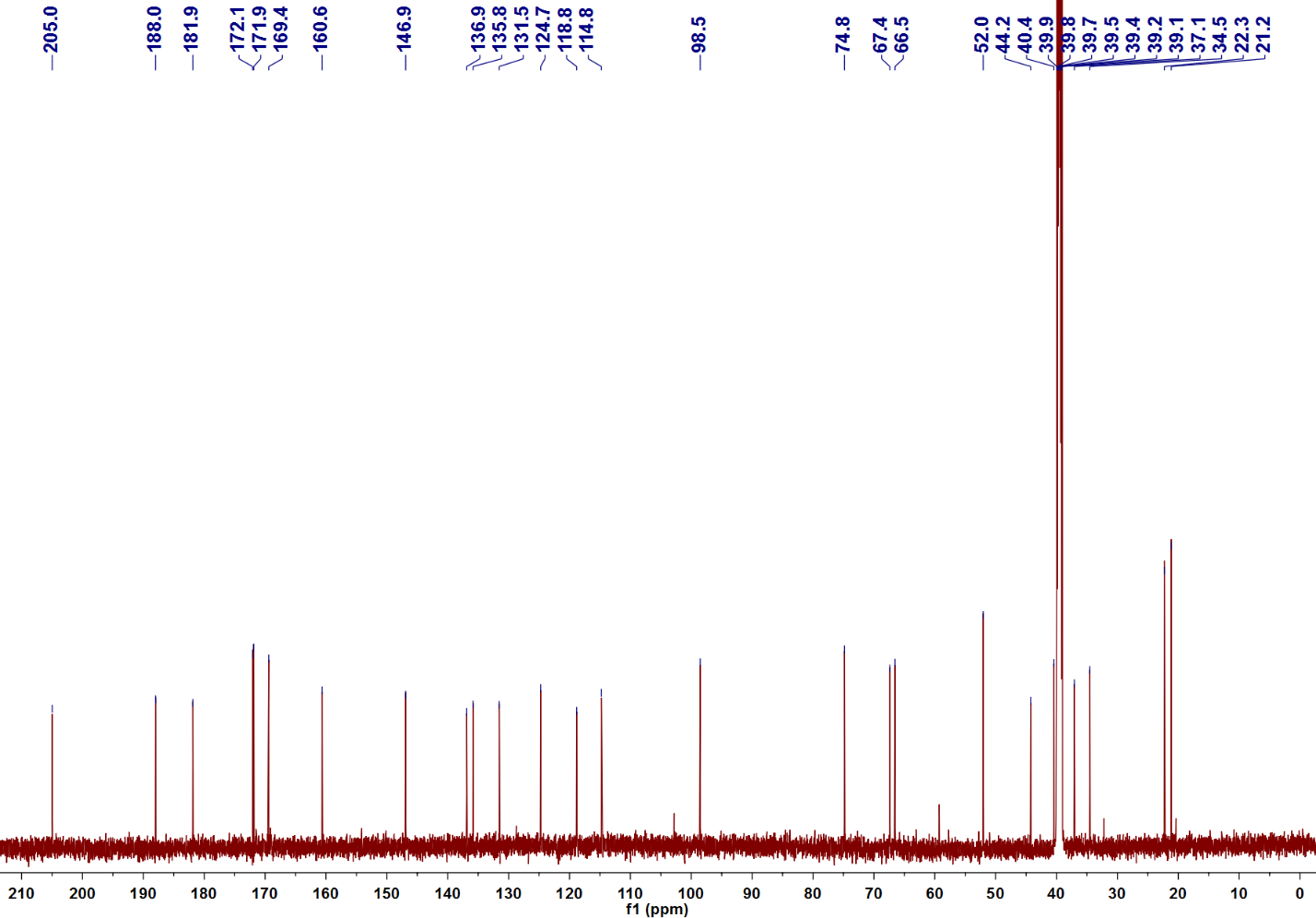


**Supplementary Fig. 97.** ^13^C NMR spectrum of *N*-acetylcysteingriseusin (**12**) in DMSO-*d*_6_.


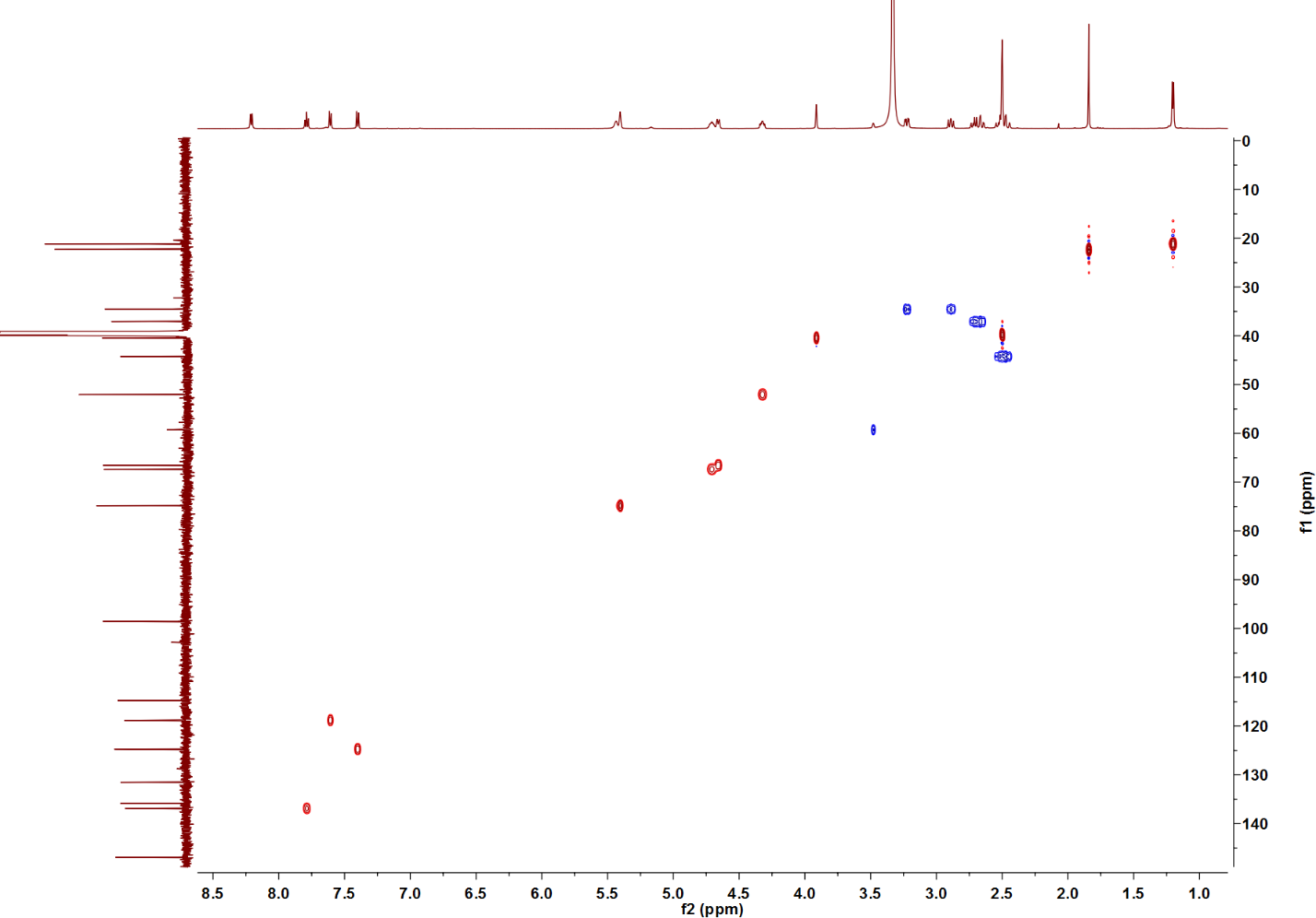


**Supplementary Fig. 98.** HSQC spectrum of *N*-acetylcysteingriseusin (**12**) in DMSO-*d*_6_.


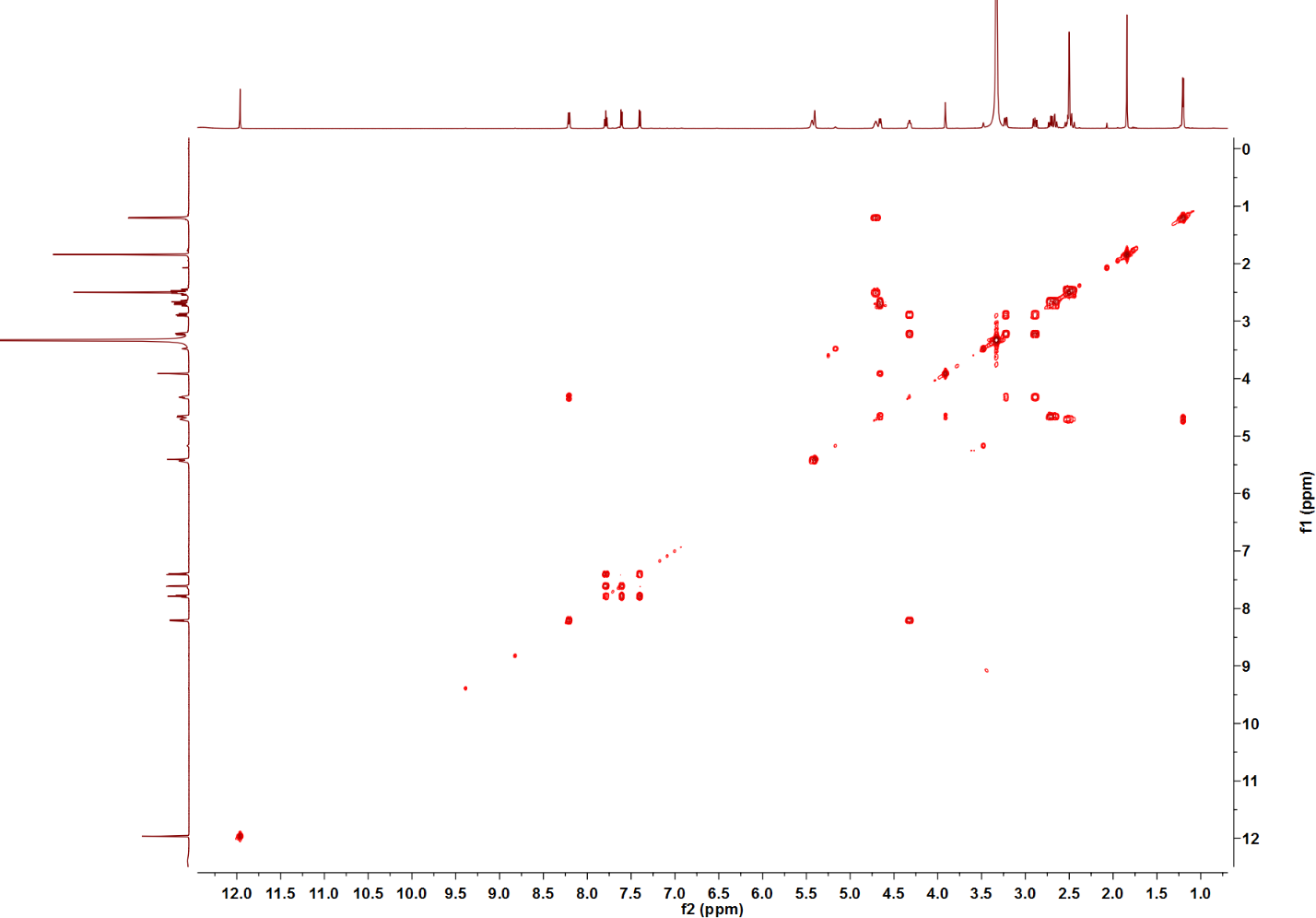


**Supplementary Fig. 99.** COSY spectrum of *N*-acetylcysteingriseusin (**12**) in DMSO-*d*_6_.


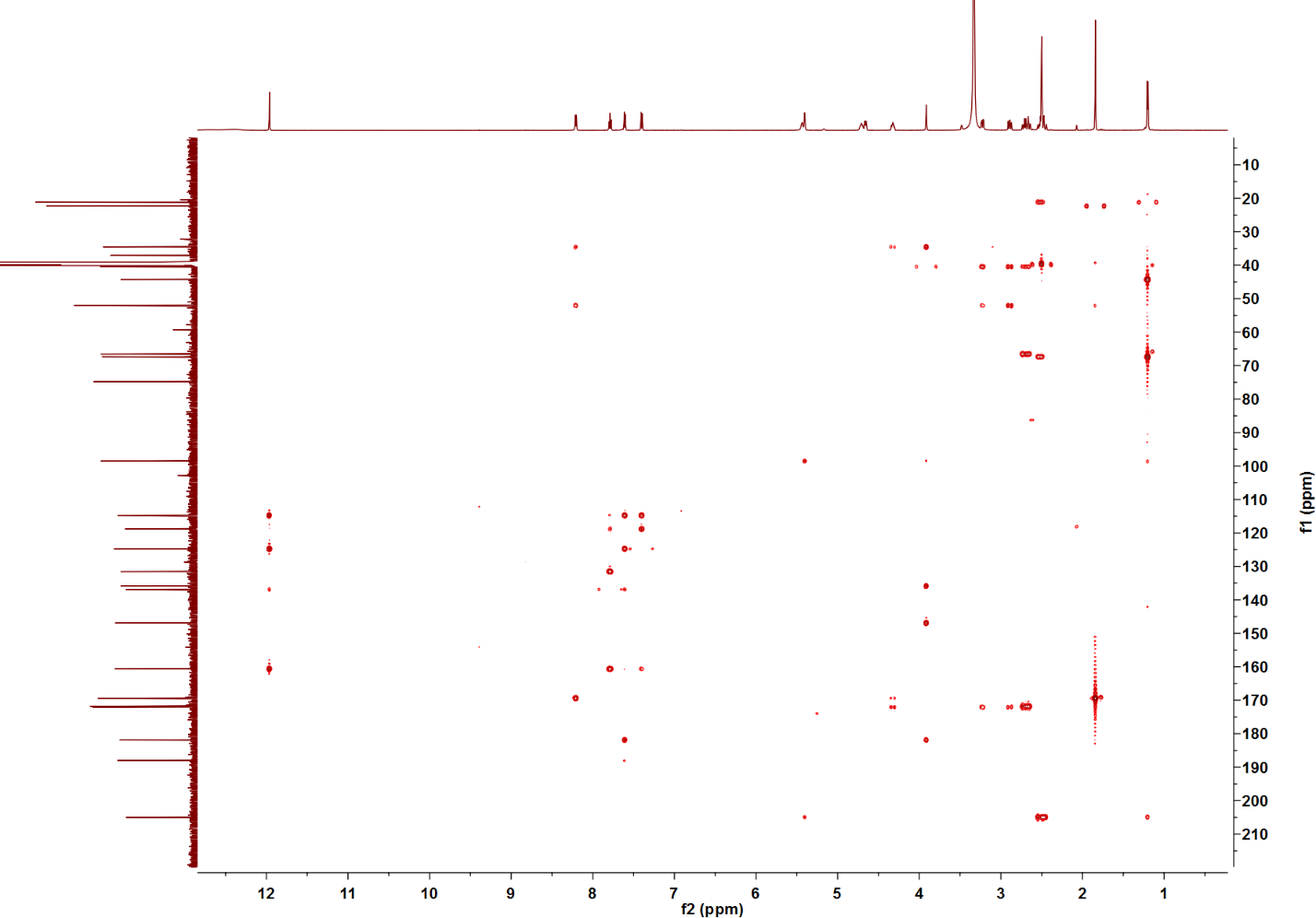


**Supplementary Fig. 100.** HMBC spectrum of *N*-acetylcysteingriseusin (**12**) in DMSO-*d*_6_.


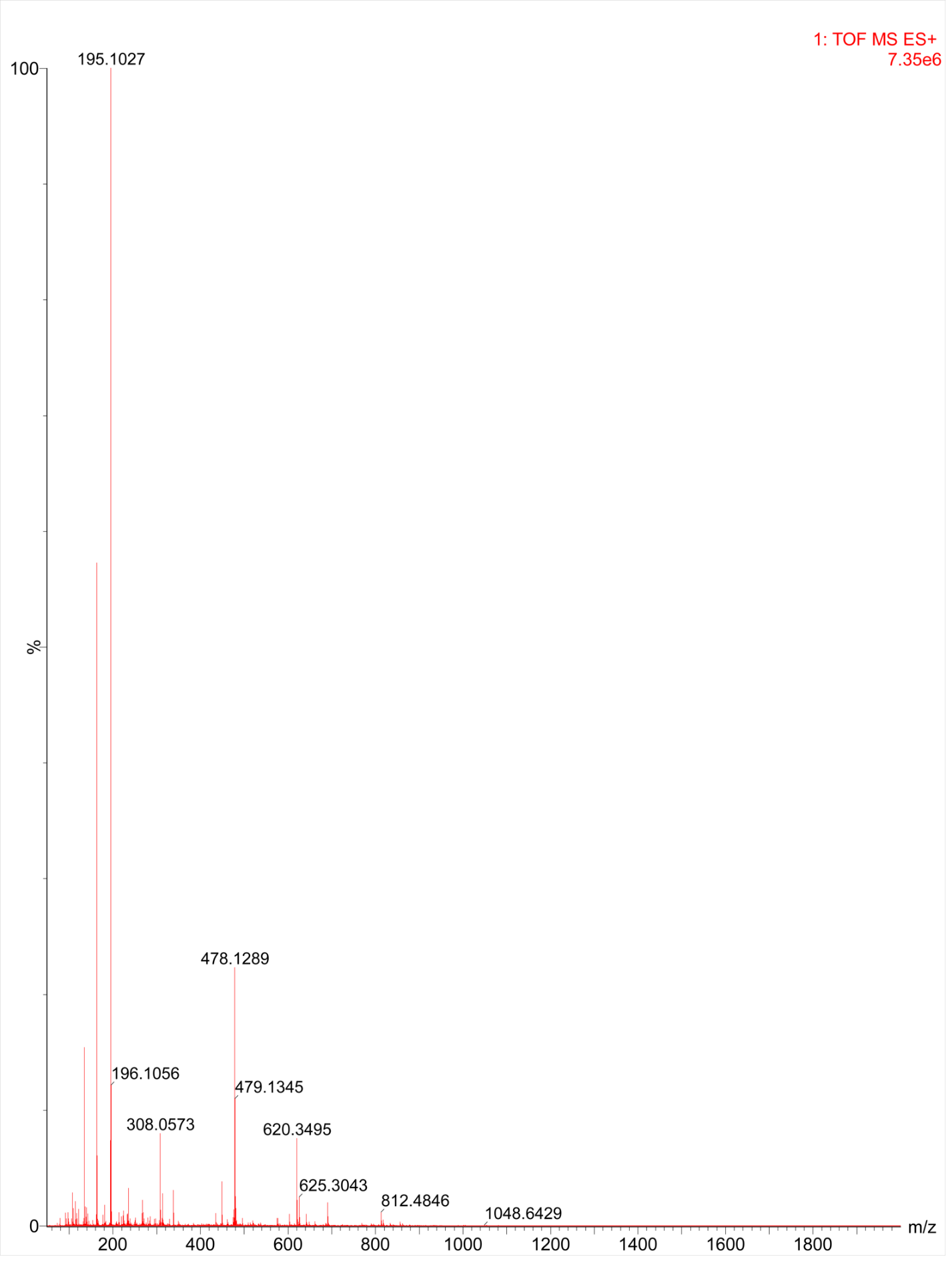


**Supplementary Fig. 101.** HRESIMS spectrum of **13**.

**Supplementary Fig. 102.** ^1^H NMR spectrum of **13** in CD_3_OD.

**Supplementary Fig. 103.** ^13^C NMR spectrum of **13** in CD_3_OD.

**Supplementary Fig. 104.** HSQC spectrum of **13** in CD_3_OD.

**Supplementary Fig. 105.** COSY spectrum of **13** in CD_3_OD.

**Supplementary Fig. 106.** HMBC spectrum of **13** in CD_3_OD.

**Supplementary Fig. 107.** RT-PCR analysis of *S. griseochromogenes* ATCC 14511 BGC #11 and *S. leeuwenhoekii* NRRL B-24963 BGC #19 heterologously expressed in *S. avermitilis* SUKA17. The selected core biosynthetic gene targets are shown in the figure. The analysis was performed at two growth time points (24 and 48 h). *hrd*B gene from *S. avermitilis* was used as internal transcription control. Each experiment was performed one time. M: 100 bp DNA ladder.

**Supplementary Fig. 108.** RT-PCR analysis of *S. griseochromogenes* ATCC 14511 BGCs #15 and #41 heterologously expressed in *S. avermitilis* SUKA17. The selected core biosynthetic gene targets are shown in the figure. For BGC #41, due to the large size of AVL59_32935 gene, two targets from start and end of the same gene were selected for RT-PCR analysis. The analysis was performed at two growth time points (24 and 48 h). *hrd*B gene from *S. avermitilis* was used as internal transcription control. Each experiment was performed one time. M: 100 bp DNA ladder.

**Supplementary Fig. 109.** RT-PCR analysis of *S. noursei* ATCC 11455 BGC #29 and *Streptomyces* sp. NRRL F-5635 BGC #10 heterologously expressed in *S. avermitilis* SUKA17. The selected core biosynthetic gene targets are shown in the figure. The analysis was performed at two growth time points (24 and 48 h). *hrd*B gene from *S. avermitilis* was used as internal transcription control. Each experiment was performed one time. M: 100 bp DNA ladder.

**Supplementary Fig. 110.** RT-PCR analysis of *Streptomyces* sp. NRRL F-6131 BGC #11 and *Streptomyces* sp. NRRL F-525 BGC #29 heterologously expressed in *S. avermitilis* SUKA17. The selected core biosynthetic gene targets are shown in the figure. The analysis was performed at two growth time points (24 and 48 h). *hrd*B gene from *S. avermitilis* was used as internal transcription control. Each experiment was performed one time. M: 100 bp DNA ladder.

**Supplementary Fig. 111.** RT-PCR analysis of *B. megaterium* QM B-1551 BGC #2 and *B. thuringiensis* YBT-020 BGC #2 heterologously expressed in *B. subtilis* JH642. The selected core biosynthetic gene targets are shown in the figure. The analysis was performed at two growth time points (24 and 48 h). *rps*E gene from *B. subtilis* was used as internal transcription control. Each experiment was performed one time. M: 100 bp DNA ladder.

Supplementary Tables

**Supplementary Table 1.** Colony numbers acquired from direct cloning of 4 high GC-content BGCs from Actinomycetes using 4 different digestion/DNA assembly combinations. Each experiment was performed in three biological replicates and data are presented as mean values +/− standard error (SEM).

|  | Holomycin (21 kb) | Actinorhodin (38 kb) | Erythromycin (62 kb) | Bafilomycin (87 kb) |
| --- | --- | --- | --- | --- |
| RE/Gibson | 95 ± 17 | 12 ± 1 | 115 ± 13 | 8 ± 4 |
| *Fn*Cas12a/ligation | 92 ± 12 | 8 ± 4 | 108 ± 31 | 34 ± 14 |
| *Fn*Cas12a/Gibson | 88 ± 7 | 21 ± 10 | 56 ± 10 | 20 ± 4 |
| *Fn*Cas12a/T4 exo + fill-in | 184 ± 87 | 146 ± 47 | 101 ± 18 | 22 ± 6 |

**Supplementary Table 2.** ^1^H NMR (600 MHz) and ^13^C NMR (150 MHz) spectroscopic data for **1** and **2**

|  | **1**^a^ | |  | **2**^b^ | |
| --- | --- | --- | --- | --- | --- |
| position | *δ*_C_, type | *δ*_H_, multi (*J* in Hz) |  | *δ*_C_, type | *δ*_H_, multi (*J* in Hz) |
| 1 | 198.3, C |  |  | 192.4, C |  |
| 2 | 63.3, C |  |  | 62.2, C |  |
| 3 | 80.2, CH | 5.28, s |  | 76.2, CH | 5.26, s |
| 3a | 162.3, C |  |  | 160.3, C |  |
| 4 | 115.2, CH | 7.33, s |  | 119.0, CH | 7.54, overlapped |
| 5 | 167.6, C |  |  | 164.2, C |  |
| 5a | 115.3, C |  |  | 117.2, C |  |
| 6 | 193.2, C |  |  | 189.8, C |  |
| 6a | 112.3, C |  |  | 112.3, C |  |
| 7 | 155.7, C |  |  | 156.3, C |  |
| 7a | 130.2, C |  |  | 134.8, C |  |
| 8 | 46.9, CH | 4.40, overlapped |  | 45.9, CH | 4.36, brs |
| 9 | 146.2, CH | 6.98, dd (1.5, 5.3) |  | 145.5, CH | 7.04, dd (1.9, 5.3) |
| 10 | 132.9, CH | 6.87, d (5.3) |  | 131.3, CH | 6.95, overlapped |
| 10a | 155.5, C |  |  | 152.9, C |  |
| 11 | 117.2, CH | 6.86, s |  | 112.4, CH | 7.21, s |
| 11a | 144.9, C |  |  | 131.3, C |  |
| 12 | 62.2, CH | 4.92, s |  | 179.8, C |  |
| 12a | 142.8, C |  |  | 131.3, C |  |
| 12b | 126.4, C |  |  | 126.5, C |  |
| 13 | 22.3, CH_3_ | 1.68, s |  | 22.1, CH_3_ | 1.57, s |
| 1' | 194.5, C |  |  | 191.2, C |  |
| 2' | 60.8, CH | 3.57, dd (2.5, 7.1) |  | 58.3, CH | 3.52, dd (2.4, 7.1) |
| 3' | 70.4, CH | 5.59, d (7.1) |  | 67.7, CH | 5.66, d (7.0) |
| 3a' | 163.9, C |  |  | 161.8, C |  |
| 4' | 120.7, CH | 7.65, s |  | 119.0, CH | 7.54, overlapped |
| 5' | 166.6, C |  |  | 164.1, C |  |
| 5a' | 118.0, C |  |  | 117.2, C |  |
| 6' | 192.7, C |  |  | 189.4, C |  |
| 6a' | 113.5, C |  |  | 112.6, C |  |
| 7' | 155.3, C |  |  | 154.0, C |  |
| 7a' | 136.9, C |  |  | 135.2, C |  |
| 8' | 52.4, CH | 4.39, overlapped |  | 50.8, CH | 4.19, brs |
| 9' | 145.7, CH | 7.16, dd (1.5, 5.4) |  | 144.2, CH | 7.07, dd (2.0, 5.6) |
| 10' | 133.7, CH | 7.02, d (5.4) |  | 132.3, CH | 6.96, overlapped |
| 10a' | 154.9, C |  |  | 152.2, C |  |
| 11' | 114.2, CH | 7.40, s |  | 112.1, CH | 7.14, s |
| 11a' | 136.8, C |  |  | 135.4, C |  |
| 12' | 181.4, C |  |  | 179.9, C |  |
| 12a' | 132.6, C |  |  | 135.4, C |  |
| 12b' | 129.0, C |  |  | 126.5, C |  |

^a^Measured in CD_3_OD; ^b^Measured in DMSO-*d*_6_.

**Supplementary Table 3.** ^1^H NMR (600 MHz) and ^13^C NMR (150 MHz) spectroscopic data for **3** and **4** in DMSO-*d*_6_ (*δ* in ppm)

|  | **3** | |  | **4** | |
| --- | --- | --- | --- | --- | --- |
| position | *δ*_C_, type | *δ*_H_, multi (*J* in Hz) |  | *δ*_C_, type | *δ*_H_, multi (*J* in Hz) |
| 1 | 196.0, C |  |  | 192.4, C |  |
| 2 | 61.2, C |  |  | 62.2, C |  |
| 3 | 77.3, CH | 5.32, s |  | 76.1, CH | 5.28, s |
| 3a | 160.5, C |  |  | 160.8, C |  |
| 4 | 113.6, CH | 7.23, s |  | 119.3, CH | 7.57, d (0.7) |
| 5 | 165.1, C |  |  | 164.1, C |  |
| 5a | 113.8, C |  |  | 116.9, C |  |
| 6 | 191.5, C |  |  | 191.1, C |  |
| 6a | 110.4, C |  |  | 111.4, C |  |
| 7 | 156.5, C |  |  | 156.1, C |  |
| 7a | 128.1, C |  |  | 134.6, C |  |
| 8 | 45.8, CH | 4.19, brs |  | 46.3, CH | 4.29, brs |
| 9 | 144.7, CH | 6.91, dd (1.9, 5.4) |  | 145.3, CH | 7.00, dd (1.7, 5.4) |
| 10 | 131.8, CH | 6.86, dd (1.0, 5.4) |  | 131.8, CH | 6.97, overlapped |
| 10a | 153.7, C |  |  | 153.7, C |  |
| 11 | 114.4, CH | 6.74, s |  | 112.4, CH | 7.22, s |
| 11a | 144.3, C |  |  | 131.6, C |  |
| 12 | 60.1, CH | 4.81, d (6.5) |  | 179.4, C |  |
| 12a | 141.8, C |  |  | 131.5, C |  |
| 12b | 124.5, C |  |  | 127.0, C |  |
| 13 | 21.6, CH_3_ | 1.57, s |  | 22.1, CH_3_ | 1.58, s |
| 1' | 189.6, C |  |  | 189.3, C |  |
| 2' | 56.5, CH | 3.53, dd (2.2, 7.1) |  | 56.3, CH | 3.59, dd (2.1, 7.1) |
| 3' | 69.4, CH | 6.56, dd (1.3, 7.1) |  | 69.2, CH | 6.61, dd (0.8, 7.1) |
| 3a' | 158.1, C |  |  | 158.3, C |  |
| 4' | 118.9, CH | 7.39, d (1.3) |  | 119.1, CH | 7.42, d (0.8) |
| 5' | 163.9, C |  |  | 164.1, C |  |
| 5a' | 117.0, C |  |  | 117.3, C |  |
| 6' | 189.5, C |  |  | 193.4, C |  |
| 6a' | 112.5, C |  |  | 112.6, C |  |
| 7' | 157.6, C |  |  | 153.9, C |  |
| 7a' | 135.1, C |  |  | 135.4, C |  |
| 8' | 50.9, CH | 4.27, brs |  | 50.7, CH | 4.20, brs |
| 9' | 144.3, CH | 7.10, dd (1.8, 5.6) |  | 144.4, CH | 7.08, dd (1.7, 6.1) |
| 10' | 132.3, CH | 7.03, d (5.6) |  | 132.3, CH | 6.96, overlapped |
| 10a' | 152.3, C |  |  | 152.1, C |  |
| 11' | 111.7, CH | 7.25, s |  | 112.1, CH | 7.15, s |
| 11a' | 135.7, C |  |  | 135.5, C |  |
| 12' | 180.0, C |  |  | 179.9, C |  |
| 12a' | 131.3, C |  |  | 134.6, C |  |
| 12b' | 126.2, C |  |  | 126.1, C |  |
| 14' | 170.9, C |  |  | 170.8, C |  |
| 15' | 20.9, CH_3_ | 2.37, s |  | 20.9, CH_3_ | 2.38, s |
| 5-OH |  | 12.42, s |  |  | 12.53, s |
| 7-OH |  | 11.38, s |  |  | 11.33, s |
| 12-OH |  | 5.66, d (6.5) |  |  |  |

**Supplementary Table 4.** ^1^H NMR (600 MHz) and ^13^C NMR (150 MHz) spectroscopic data for **5** and **6**

|  | **5**^a^ | |  | **6**^b^ | |
| --- | --- | --- | --- | --- | --- |
| position | *δ*_C_, type | *δ*_H_, multi (*J* in Hz) |  | *δ*_C_, type | *δ*_H_, multi (*J* in Hz) |
| 1 | 198.7, C |  |  | 192.4, C |  |
| 2 | 63.1, C |  |  | 62.2, C |  |
| 3 | 80.2, CH | 5.38, s |  | 76.1, CH | 5.28, s |
| 3a | 162.5, C |  |  | 160.8, C |  |
| 4 | 114.7, CH | 7.35, s |  | 119.3, CH | 7.57, d (0.9) |
| 5 | 167.7, C |  |  | 164.1, C |  |
| 5a | 114.9, C |  |  | 116.9, C |  |
| 6 | 193.1, C |  |  | 191.1, C |  |
| 6a | 112.0, C |  |  | 111.5, C |  |
| 7 | 158.2, C |  |  | 156.1, C |  |
| 7a | 129.8, C |  |  | 134.5, C |  |
| 8 | 47.1, CH | 4.29, brs |  | 46.2, CH | 4.19, brs |
| 9 | 145.5, CH | 6.96, dd (1.9, 5.4) |  | 145.3, CH | 7.01, dd (1.9, 5.4) |
| 10 | 133.3, CH | 6.89, dd (1.0, 5.4) |  | 131.8, CH | 6.97, overlapped |
| 10a | 155.2, C |  |  | 153.8, C |  |
| 11 | 116.2, CH | 6.91, s |  | 112.4, CH | 7.22, s |
| 11a | 144.3, C |  |  | 131.7, C |  |
| 12 | 62.3, CH | 4.74, s |  | 179.4, C |  |
| 12a | 143.7, C |  |  | 132.0, C |  |
| 12b | 126.8, C |  |  | 127.0, C |  |
| 13 | 22.5, CH_3_ | 1.75, s |  | 22.1, CH_3_ | 1.58, s |
| 1' | 190.3, C |  |  | 189.2, C |  |
| 2' | 58.3, CH | 3.71, dd (2.2, 7.1) |  | 56.4, CH | 3.62, dd (2.2, 7.1) |
| 3' | 70.7, CH | 6.64, dd (1.3, 7.1) |  | 69.1, CH | 6.61, dd (0.8, 7.1) |
| 3a' | 160.2, C |  |  | 158.3, C |  |
| 4' | 120.6, CH | 7.49, d (1.3) |  | 118.8, CH | 7.36, d (1.0) |
| 5' | 166.0, C |  |  | 164.0, C |  |
| 5a' | 117.7, C |  |  | 117.4, C |  |
| 6' | 193.0, C |  |  | 190.8, C |  |
| 6a' | 113.1, C |  |  | 112.6, C |  |
| 7' | 156.1, C |  |  | 153.7, C |  |
| 7a' | 136.2, C |  |  | 135.5, C |  |
| 8' | 52.1, CH | 4.46, brs |  | 50.7, CH | 4.20, brs |
| 9' | 145.9, CH | 7.27, dd (2.1, 5.6) |  | 144.4, CH | 7.08, dd (1.9, 5.5) |
| 10' | 133.7, CH | 7.14, dd (1.3, 5.6) |  | 132.3, CH | 6.96, overlapped |
| 10a' | 155.1, C |  |  | 152.3, C |  |
| 11' | 114.1, CH | 7.46, s |  | 112.1, CH | 7.16, s |
| 11a' | 136.9, C |  |  | 135.4, C |  |
| 12' | 180.2, C |  |  | 179.9, C |  |
| 12a' | 132.9, C |  |  | 134.7, C |  |
| 12b' | 128.7, C |  |  | 126.2, C |  |
| 14' | 177.4, C |  |  | 176.2, C |  |
| 15' | 35.2, CH | 3.05, m |  | 33.6, CH | 2.94, m |
| 16' | 19.6, CH_3_ | 1.49, d (7.0) |  | 18.8, CH_3_ | 1.36, d (7.0) |
| 17' | 19.7, CH_3_ | 1.50, d (7.0) |  | 18.8, CH_3_ | 1.37, d (7.0) |
| 5-OH |  | 12.64, s |  |  | 12.47, s |
| 7-OH |  | 11.65, s |  |  | 11.43, s |

^a^Measured in (CD_3_)_2_CO/CDCl_3_; ^b^Measured in DMSO-*d*_6_.

**Supplementary Table 5.** Crystal data and structure refinement for **5**

| Identification code | bipentaromycin E |
| --- | --- |
| Empirical formula | C_45_H_30_O_13_ |
| Formula weight | 778.69 |
| Temperature/K | 100.00 (2) |
| Crystal system | monoclinic |
| Space group | P2_1_ |
| a/Å | 15.1436(10) |
| b/Å | 8.7044(6) |
| c/Å | 15.6080(11) |
| α/° | 90 |
| β/° | 114.2341(12) |
| γ/° | 90 |
| Volume/Å^3^ | 1876.1(2) |
| Z | 2 |
| ρ_calc_g/cm^3^ | 1.590 |
| μ/mm^‑1^ | 2.860 |
| F(000) | 924.0 |
| Crystal size/mm^3^ | 0.276 × 0.186 × 0.062 |
| Radiation | Cu Kα (λ = 1.54178) |
| 2Θ range for data collection/° | 6.21 to 136.572 |
| Index ranges | -18 ≤ h ≤ 18, -10 ≤ k ≤ 10, -18 ≤ l ≤ 18 |
| Reflections collected | 39624 |
| Independent reflections | 6860 [R_int_ = 0.0351, R_sigma_ = 0.0242] |
| Data/restraints/parameters | 6860/97/617 |
| Goodness-of-fit on F^2^ | 1.085 |
| Final R indexes [I>=2σ (I)] | R_1_ = 0.0373, wR_2_ = 0.0977 |
| Final R indexes [all data] | R_1_ = 0.0373, wR_2_ = 0.0978 |
| Largest diff. peak/hole / e Å^-3^ | 0.55/-0.52 |
| Flack parameter | 0.007(5) |

**Supplementary Table 6. Antimicrobial activities of compounds 3−6.**

| Strains |  | MIC (μg/mL) | | | | |
| --- | --- | --- | --- | --- | --- | --- |
|  |  | **3** | **4** | **5** | **6** | Amp |
| Bacteria |  |  |  |  |  |  |
| *Acinetobacter baumannii* ATCC 19606 | G- | >64 | >64 | >64 | >64 | >64 |
| *Bacillus anthracis* str. Sterne | G+ | 4 | 4 | 4 | 4 | 0.25 |
| *Bacillus cereus* TZ417 | G+ | 8 | 8 | 8 | 8 | 32 |
| *Bacillus halodurans* C-125 | G+ | 8 | 8 | 8 | 8 | >64 |
| *Bacillus subtilis* ATCC 6633 | G+ | 32 | 32 | >64 | >64 | >64 |
| *Enterobacter cloacae* | G- | >64 | >64 | >64 | >64 | >64 |
| *Enterococcus faecium* U503 | G+ | 32 | 32 | 32 | 32 | >64 |
| *Escherichia coli* DH5a | G- | >64 | >64 | >64 | >64 | 32 |
| *Klebsiella pneumoniae* ATCC 27736 | G- | >64 | >64 | >64 | >64 | >64 |
| *Lactococcus lactis* CNRZ 481 | G+ | 16 | 16 | 32 | 32 | 0.25 |
| *Micrococcus luteus* ATCC 4698 | G+ | 16 | 16 | 16 | 16 | 0.25 |
| *Pseudomonas aeruginosa* PA01 | G- | >64 | >64 | >64 | >64 | >64 |
| *Pseudomonas fluorescens* Pf-5 | G- | 16 | 16 | 32 | 32 | >64 |
| *Pseudomonas putida* mt-2 | G- | 16 | 16 | 32 | 32 | >64 |
| *Staphylococcus aureus* USA300 | G+ | 32 | 32 | 32 | 32 | >64 |
| *Staphylococcus epidermidis* 15X154 | G+ | 4 | 4 | 32 | 32 | 2 |
| *Streptococcus mutans* ATCC 25175 | G+ | 16 | 16 | >64 | >64 | 0.25 |
|  |  |  |  |  |  |  |
| Fungi |  |  |  |  |  |  |
| *Saccharomyces cerevisiae* YSG50 |  | >64 | >64 | >64 | >64 | - |
| *Saccharomyces cerevisiae* BY4741 |  | >64 | >64 | >64 | >64 | - |
| *Aspergillus nidulans* |  | >64 | >64 | >64 | >64 | - |
| *Aspergillus niger* |  | >64 | >64 | >64 | >64 | - |
| *Aspergillus terreus* |  | >64 | >64 | >64 | >64 | - |

G+: gram positive; G-: gram negative

**Supplementary Table 7.** ^1^H NMR (600 MHz) and ^13^C NMR (150 MHz) spectroscopic data for **7** and **8** in DMSO-*d*_6_ (*δ* in ppm)

|  | **7** | |  | **8** | |
| --- | --- | --- | --- | --- | --- |
| position | *δ*_C_, type | *δ*_H_, multi (*J* in Hz) |  | *δ*_C_, type | *δ*_H_, multi (*J* in Hz) |
| 1 | 196.0, C |  |  | 196.6, C |  |
| 2 | 44.5, CH_2_ | 2.44, dd (10.7, 16.6) |  | 42.4, CH_2_ | 2.46, dd (7.9, 16.6) |
|  |  | 2.87, dd (5.9, 16.6) |  |  | 2.81, dd (6.4, 16.6) |
| 3 | 37.0, CH | 2.14, m |  | 34.0, CH | 2.40, m |
| 4 | 71.5, CH | 4.31, dd (6.8, 9.3) |  | 69.4, CH | 4.65, dd (2.9, 3.5) |
| 4a | 156.4, C |  |  | 154.7, C |  |
| 5 | 118.7, CH | 7.39, s |  | 120.1, CH | 7.27, s |
| 6 | 162.6, C |  |  | 162.5, C |  |
| 6a | 117.3, C |  |  | 117.4, C |  |
| 7 | 190.9, C |  |  | 191.0, C |  |
| 7a | 115.6, C |  |  | 115.7, C |  |
| 8 | 160.7, C |  |  | 160.7, C |  |
| 9 | 123.8, CH | 7.36, d (8.3) |  | 123.8, CH | 7.36, dd (1.1, 8.5) |
| 10 | 137.5, CH | 7.79, dd (7.4, 8.3) |  | 137.5, CH | 7.79, dd (7.4, 8.5) |
| 11 | 118.7, CH | 7.50, d (7.4) |  | 118.7, CH | 7.50, dd (1.1, 7.4) |
| 11a | 135.2, C |  |  | 135.2, C |  |
| 12 | 182.9, C |  |  | 182.8, C |  |
| 12a | 136.9, C |  |  | 136.8, C |  |
| 12b | 127.8, C |  |  | 128.0, C |  |
| 13 | 18.1, CH_3_ | 1.12, d (6.5) |  | 15.8, CH_3_ | 1.02, d (6.7) |
| 4-OH |  | 5.98, d (6.8) |  |  | 5.81, d (3.5) |
| 6-OH |  |  |  |  | 11.60, s |
| 8-OH |  |  |  |  | 12.26, s |

**Supplementary Table 8.** ^1^H NMR (600 MHz) and ^13^C NMR (150 MHz) spectroscopic data for **9** and **10** in DMSO-*d*_6_ (*δ* in ppm)

|  | **9** | |  | **10** | |
| --- | --- | --- | --- | --- | --- |
| position | *δ*_C_, type | *δ*_H_, multi (*J* in Hz) |  | *δ*_C_, type | *δ*_H_, multi (*J* in Hz) |
| 1 | 204.2, C |  |  | 156.6, C |  |
| 2 | 45.6, CH | 2.60, d (18.2) |  | 123.8, CH | 7.18, s |
|  |  | 3.15, d (18.2) |  |  |  |
| 3 | 72.2, C |  |  | 138.8, C |  |
| 4 | 61.0, CH | 4.32, s |  | 119.4, CH | 7.57, s |
| 4a | 134.2, C |  |  | 129.9, C |  |
| 5 | 118.3, CH | 7.26, d (8.1) |  | 180.2, C |  |
| 6 | 132.5, CH | 7.53, dd (7.9, 8.1) |  | 148.7, C |  |
| 6a |  |  |  | 105.3, C |  |
| 7 | 111.1, CH | 6.84, d (7.9) |  | 183.3, C |  |
| 7a |  |  |  | 114.6, C |  |
| 8 | 157.3, C |  |  | 161.6, C |  |
| 8a | 112.6, C |  |  |  |  |
| 9 | 164.6, C |  |  | 114.8, CH | 6.83, d (8.1) |
| 9a | 109.0, C |  |  |  |  |
| 10 | 118.6, C | 7.28, s |  | 135.1, CH | 7.53, dd (7.6, 8.1) |
| 10a | 138.9, C |  |  |  |  |
| 11 | 207.2, C |  |  | 120.0, CH | 7.12, d (7.6) |
| 11a |  |  |  | 138.4, C |  |
| 12 | 32.1, CH_3_ | 2.38, s |  | 123.7, CH | 8.84, s |
| 12a |  |  |  | 127.0, C |  |
| 12b |  |  |  | 119.0, C |  |
| 13 | 41.9, CH_2_ | 1.47, overlapped |  | 20.6, CH_3_ | 2.35, s |
|  |  | 1.57, m |  |  |  |
| 14 | 15.3, CH_2_ | 1.49, overlapped |  |  |  |
| 15 | 14.4, CH_3_ | 0.92, t (6.9) |  |  |  |
| 1-OH |  |  |  |  | 10.96, s |
| 4a-OH |  | 5.03, s |  |  |  |
| 6-NH_2_ |  |  |  |  | 9.06, s |
|  |  |  |  |  | 10.88, s |
| 8-OH |  | 9.81, s |  |  | 13.64, s |

**Supplementary Table 9.** ^1^H NMR (600 MHz) and ^13^C NMR (150 MHz) spectroscopic data for **11** and **12** in DMSO-*d*_6_ (*δ* in ppm)

|  | **11** | |  | **12** | |  |
| --- | --- | --- | --- | --- | --- | --- |
| position | *δ*_C_, type | *δ*_H_, multi (*J* in Hz) |  | *δ*_C_, type | *δ*_H_, multi (*J* in Hz) |  |
| 1 | 158.0, C |  |  | 171.9, C |  |  |
| 2 | 135.0, C |  |  | 37.1, CH_2_ | 2.65, dd (3.1, 15.8) |  |
|  |  |  |  |  | 2.72, dd (9.8, 15.8) |  |
| 3 | 145.0, C |  |  | 66.5, CH | 4.66, m |  |
| 4 | 121.3, CH | 7.60, s |  | 40.4, CH | 3.91, brs |  |
| 4a | 133.0, C |  |  |  |  |  |
| 5 | 119.5, CH | 7.72, d (7.4) |  | 135.8, C |  |  |
| 6 | 137.6, CH | 7.82, dd (7.4, 8.3) |  | 181.9, C |  |  |
| 7 | 124.6, CH | 7.40, d (8.3) |  | 131.5, C |  |  |
| 8 | 161.3, C |  |  | 118.8, CH | 7.61, d (7.5) |  |
| 8a | 115.9, C |  |  |  |  |  |
| 9 | 191.6, C |  |  | 136.9, CH | 7.79, dd (7.5, 8.5) |  |
| 9a | 114.4, C |  |  |  |  |  |
| 10 | 181.1, C |  |  | 124.7, CH | 7.40, d (8.5) |  |
| 10a | 133.2, C |  |  |  |  |  |
| 11 | 203.1, C |  |  | 160.6, C |  |  |
| 12 | 50.9, CH_2_ | 3.14, m |  | 114.8, C |  |  |
| 13 | 35.0, CH | 3.37, overlapped |  | 188.0, C |  |  |
| 14 | 21.4, CH_3_ | 1.33, d (6.7) |  | 146.9, C |  |  |
| 15 | 19.6, CH_3_ | 2.35, s |  | 98.5, C |  |  |
| 16 |  |  |  | 74.8, CH | 5.41, s |  |
| 17 |  |  |  | 205.0, C |  |  |
| 18 |  |  |  | 44.2, CH_2_ | 2.46, dd (2.8, 18.2) |  |
|  |  |  |  |  | 2.52, dd (11.4, 18.2) |  |
| 19 |  |  |  | 67.4, CH | 4.71, m |  |
| 20 |  |  |  | 21.2, CH_3_ | 1.20, d 5.9 |  |
| 1′ | 31.7, CH_2_ | 2.93, dd (4.9, 13.5) |  | 34.5, CH_2_ | 2.89, dd (10.2, 13.2) |  |
|  |  | 2.80, dd (8.2, 13.5) |  |  | 3.22, dd (4.4, 13.2) |  |
| 2′ | 52.4, CH | 4.36, m |  | 52.0, CH | 4.32, m |  |
| 3′ | 172.1, C |  |  | 172.1, C |  |  |
| 4′ | 169.3, C |  |  | 169.4, C |  |  |
| 5′ | 22.3, CH_3_ | 1.84, s |  | 22.3, CH_3_ | 1.84, s |  |
| 11-OH |  |  |  |  | 11.96, s |  |
| NH |  | 8.22, d (7.8) |  |  | 8.21, d (7.9) |  |

**Supplementary Table 10.** ^1^H NMR (600 MHz) and ^13^C NMR (150 MHz) spectroscopic data for **13** in CD_3_OD (*δ* in ppm)

|  | **13** | |
| --- | --- | --- |
| position | *δ*_C_, type | *δ*_H_, multi (*J* in Hz) |
| 1 | 170.2, C |  |
| 2 | 128.8, C |  |
| 3 | 138.6, CH | 7.18, dq (1.3, 11.5) |
| 4 | 126.1, CH | 6.77, dd (11.5, 15.1) |
| 5 | 144.0, CH | 6.22, d (15.1) |
| 6 | 81.1, C |  |
| 7 | 211.8, C |  |
| 8 | 24.7, CH_3_ | 2.22, s |
| 2-Me | 12.8, CH_3_ | 1.96, d (1.3) |
| 6-Me | 25.5, CH_3_ | 1.42, s |
| OMe | 52.4, CH_3_ | 3.74, s |

**Supplementary Table 11.** Comparison of CAPTURE vs. current direct cloning strategies.

| **Direct cloning method** |  |
| --- | --- |
| TAR cloning^2,3^ | **Disadvantages:**  - Use of homologous recombination for cloning (unable to clone target BGCs with repetitive sequences)  - Can be technically challenging  - Must use yeast  - BGC-specific receiver vectors must first be assembled into a circular plasmid and then digested and used for cloning  - Constructs should be shuttled into *E. coli* after cloning in yeast  - Low cloning efficiency  - Long cloning timeframe (longer than 10 days)  - Extensive colony screening |
| RecET LLHR^4^ | **Disadvantages:**  - Use of homologous recombination for cloning (unable to clone target BGCs with repetitive sequences)  - Use of long (~100 nt) oligonucleotides for receiver amplification by PCR, which results in high cost  - Unable to clone target BGCs larger than 50 kb  - Cloning targets into BAC heterologous expression vectors is challenging due to large size of DNA receivers amplified by PCR (>10 kb)  - Large target BGCs must be divided into multiple pieces and then reassembled to enable cloning^5^ |
| ExoCET^6^ | **Disadvantages:**  - Use of homologous recombination for cloning (unable to clone target BGCs with repetitive sequences)  - Use of long (~100 nt) oligonucleotides for receiver amplification by PCR, which results in high cost  - Cloning target BGCs into BAC heterologous expression vectors is challenging due to large size of DNA receivers amplified by PCR (>10 kb)  - Large target BGCs must be divided into multiple pieces and then reassembled to enable cloning^7^  - Low efficiency for large target BGCs (4-8% for 106 kb salinomycin BGC from *S. albus*) |
| CATCH^8^ | **Disadvantages:**  - Low efficiency for large target BGCs from high GC-content genomes (5% for an 83 kb target BGC from *S. coelicolor*)^9^  - Technically challenging preparation and digestion of genomic DNA in agarose gel plugs  - Use of *E. coli* commercial electrocompetent cells, which results in high cost  - Cloning target BGCs into BAC heterologous expression vectors is challenging due to large size of DNA receivers amplified by PCR (>10 kb) |
| **CAPTURE** (this work) | **Advantages:**  - BGCs can be cloned in 3-4 days  - Use of site-specific recombination enables cloning target BGCs with repetitive sequences  - Use of short (<60 nt) oligonucleotides for PCR, which results in low cost  - Use of two DNA receivers instead of one enables cloning target BGCs into vectors up to 14 kb in size without PCR difficulties  - ~100% cloning efficiency for all target BGCs in 10-113 kb range including 100+ kb target BGCs from high-GC content genomes  - Use of in-lab prepared electrocompetent *E. coli* cells, which results in low cost  - Extremely robust: 47 BGCs cloned from both Actinomycetes and Bacilli without any failure |

Supplementary References

1 Vologodskii, A. *Biophysics of DNA*. (Cambridge University Press, 2015).

2 Kouprina, N., Noskov, V. N. & Larionov, V. Selective isolation of large segments from individual microbial genomes and environmental DNA samples using transformation-associated recombination cloning in yeast. *Nature Protocols* **15**, 734-749 (2020).

3 Yamanaka, K. *et al.* Direct cloning and refactoring of a silent lipopeptide biosynthetic gene cluster yields the antibiotic taromycin A. *Proceedings of the National Academy of Sciences USA* **111**, 1957-1962 (2014).

4 Fu, J. *et al.* Full-length RecE enhances linear-linear homologous recombination and facilitates direct cloning for bioprospecting. *Nature Biotechnology* **30**, 440 (2012).

5 Yin, J. *et al.* Direct cloning and heterologous expression of the salinomycin biosynthetic gene cluster from *Streptomyces albus* DSM41398 in *Streptomyces coelicolor* A3 (2). *Scientific Reports* **5**, 1-8 (2015).

6 Wang, H. *et al.* ExoCET: exonuclease in vitro assembly combined with RecET recombination for highly efficient direct DNA cloning from complex genomes. *Nucleic Acids Research* **46**, e28-e28 (2018).

7 Song, C. *et al.* Enhanced heterologous spinosad production from a 79-kb synthetic multioperon assembly. *ACS Synthetic Biology* **8**, 137-147 (2018).

8 Jiang, W. *et al.* Cas9-Assisted Targeting of CHromosome segments CATCH enables one-step targeted cloning of large gene clusters. *Nature Communications* **6**, 1-8 (2015).

9 Jiang, W. & Zhu, T. F. Targeted isolation and cloning of 100-kb microbial genomic sequences by Cas9-assisted targeting of chromosome segments. *Nature Protocols* **11**, 960 (2016).
