# Supplementary material for: Cas12a-assisted precise targeted cloning using in vivo Cre-lox recombination
Source: Nat Commun. 2021 Feb 19;12:1171. doi: 10.1038/s41467-021-21275-4 (PMC7896053; doi:10.1038/s41467-021-21275-4)
Supplement: Supplementary file 3 — Reporting Summary [file 41467_2021_21275_MOESM3_ESM.pdf]

nature research

Corresponding author(s): Huimin Zhao

Last updated by author(s): Oct 2, 2020

Reporting Summary

Nature Research wishes to improve the reproducibility of the work that we publish. This form provides structure for consistency and transparency in reporting. For further information on Nature Research policies, see our Editorial Policies and the Editorial Policy Checklist.

Statistics

For all statistical analyses, confirm that the following items are present in the figure legend, table legend, main text, or Methods section.

n/a	Confirmed	
[ ]	[x]	The exact sample size (*n*) for each experimental group/condition, given as a discrete number and unit of measurement
[ ]	[x]	A statement on whether measurements were taken from distinct samples or whether the same sample was measured repeatedly
[x]	[ ]	The statistical test(s) used AND whether they are one- or two-sided<br>*Only common tests should be described solely by name; describe more complex techniques in the Methods section.*
[x]	[ ]	A description of all covariates tested
[x]	[ ]	A description of any assumptions or corrections, such as tests of normality and adjustment for multiple comparisons
[ ]	[x]	A full description of the statistical parameters including central tendency (e.g. means) or other basic estimates (e.g. regression coefficient) AND variation (e.g. standard deviation) or associated estimates of uncertainty (e.g. confidence intervals)
[x]	[ ]	For null hypothesis testing, the test statistic (e.g. *F*, *t*, *r*) with confidence intervals, effect sizes, degrees of freedom and *P* value noted<br>*Give P values as exact values whenever suitable.*
[x]	[ ]	For Bayesian analysis, information on the choice of priors and Markov chain Monte Carlo settings
[x]	[ ]	For hierarchical and complex designs, identification of the appropriate level for tests and full reporting of outcomes
[x]	[ ]	Estimates of effect sizes (e.g. Cohen's *d*, Pearson's *r*), indicating how they were calculated

Our web collection on statistics for biologists contains articles on many of the points above.

Software and code

Policy information about availability of computer code

Data collection	Putative uncharacterized BGCs were selected by analysis of microbial genomes using the antiSMASH (version 5.0) software.
Data analysis	BGCs DNA sequence were analyzed by SnapGene viewer software (version 5.0). NMR (nuclear magnetic resonance) data were analyzed by MestReNova (version 11.0.3). High-resolution electrospray ionization mass spectrometric (HRESIMS) data were analyzed by MassLynx (version 4.1). X-ray single crystal were visualized by Olex2 (version 1.3.0). Bipentaromycin structures were drawn using ChemBioDraw (version 14.0).

For manuscripts utilizing custom algorithms or software that are central to the research but not yet described in published literature, software must be made available to editors and reviewers. We strongly encourage code deposition in a community repository (e.g. GitHub). See the Nature Research guidelines for submitting code & software for further information.

Data

Policy information about availability of data

All manuscripts must include a data availability statement. This statement should provide the following information, where applicable:

- Accession codes, unique identifiers, or web links for publicly available datasets
- A list of figures that have associated raw data
- A description of any restrictions on data availability

Deposition number of crystallographic data for 5 is CCDC 1993850. The data supporting the findings of this study are available within the article and its Supplementary Information files and all other data are available from the corresponding authors upon reasonable request.

Field-specific reporting

Please select the one below that is the best fit for your research. If you are not sure, read the appropriate sections before making your selection.

☒ Life sciences ☐ Behavioural & social sciences ☐ Ecological, evolutionary & environmental sciences

For a reference copy of the document with all sections, see nature.com/documents/nr-reporting-summary-flat.pdf

Life sciences study design

All studies must disclose on these points even when the disclosure is negative.

Sample size	No statistical methods were used to predetermine sample size. All method development experiments were performed with at least three biological replicates. This sample sizes was chosen due to time and budget considerations. The results obtained suggest the chosen sample size was appropriate because clear distinctions can be seen with the current sample size.
Data exclusions	No data was excluded from analysis. All the experimental/analysis results are included in the manuscript or Supplementary Information.
Replication	Direct cloning of the four model BGCs was repeated with three biological replicates. For the uncharacterized BGC clonings, the clonings for three largest BGCs (>100 kb) were repeated multiple times with similar results. For other cloning experiments, due to time considerations, each experiment was only performed once. As no cloning failures were observed, we did not find it necessary to replicate these experiments. For RT-PCR experiments, due to time and budget limitations and the large number of checked BGCs, each experiment was only performed once. As internal controls were functional for each experiment, we did not find it necessary to replicate these experiments.
Randomization	No randomization was performed as none of the experiments involved allocation of samples to test groups.
Blinding	Not relevant because no group allocation was involved in this study.

Reporting for specific materials, systems and methods

We require information from authors about some types of materials, experimental systems and methods used in many studies. Here, indicate whether each material, system or method listed is relevant to your study. If you are not sure if a list item applies to your research, read the appropriate section before selecting a response.

Materials & experimental systems

n/a	Involved in the study	
☒	☐	Antibodies
☒	☐	Eukaryotic cell lines
☒	☐	Palaeontology and archaeology
☒	☐	Animals and other organisms
☒	☐	Human research participants
☒	☐	Clinical data
☒	☐	Dual use research of concern

Methods

n/a	Involved in the study	
☒	☐	ChIP-seq
☒	☐	Flow cytometry
☒	☐	MRI-based neuroimaging
