# Supplementary material for: Cas12a-assisted precise targeted cloning using in vivo Cre-lox recombination
Source: Nat Commun. 2021 Feb 19;12:1171. doi: 10.1038/s41467-021-21275-4 (PMC7896053; doi:10.1038/s41467-021-21275-4)
Supplement: Supplementary file 5 — Description of Additional Supplementary Files [file 41467_2021_21275_MOESM5_ESM.pdf]

Title: Supplementary Data 1.

Description: Oligonucleotides used in this study
